# Supplementary material for: PERFECTED enhanced recovery pathway (PERFECT-ER) versus standard acute hospital care for people after hip fracture surgery who have cognitive impairment: a feasibility cluster randomised controlled trial
Source: BMJ Open. 2022 Feb 28;12(2):e055267. doi: 10.1136/bmjopen-2021-055267 (PMC8886407; doi:10.1136/bmjopen-2021-055267)
Supplement: Supplementary data [file bmjopen-2021-055267supp001.pdf]

Supplementary Table 1: Data collection schedule:

Statement of authorship: Table created by the authors

	**Admission**	**Enrolment**	**Baseline**	**Post-operative period**			
**TIMEPOINT**	**-T2**	**-T1**	**0**	**D**	**T1[a]**	**T2**	**T3**
PRE-INTERVENTION:							
Eligibility screen							
Study information provided							
Informed consent given							
ASSESSMENTS:							
MMSE-2: SV (Patient)							
DEMQOL (Patient)							
EQ-5D-5L self-complete (Patient)							
howRwe (Patient)				b	b		
CDR (Patient)							
Patient care profile (Patient)				b	b		
Timed Up & Go (Patient)							
BADLS (Suitable Informant)							
DEMQOL-Proxy (Suitable Informant)							
EQ-5D-5L Proxy (Suitable Informant)							
EQ-5D-5L Carer self-report (Suitable Informant)							
CSRI[c] (Suitable Informant)							
Number of days in institutional care (Suitable Informant)							
howRthey (Suitable Informant)				b	b		
Patient's place of residence (Suitable Informant)			d				
CDR (Suitable Informant)							
IQCODE (Suitable Informant)							
Length of stay in index hospitalisation				e	e		
Discharge destination from index hospitalisation							
Mortality							
Hospital re-admission rates							
Hospital service use[f]							
4AT				b	b		
Charlson Co-morbidity Index (CCI)							
NHFD (England only)							g

[a] PERFECT-ER and treatment as usual continue up until discharge from study ward. Due to differences in length of stay in the study sites, T1 assessments may take place in the study site for some participants;
[b] Patients may be discharged from study ward before or after T1. Measure to be collected at whenever this point maybe ± five days;
[c] duration of retrospective period covered varies by assessment point;
[d] pre-baseline ordinary residence;
[e] If patient is still in acute hospital at thirty days this will be recorded;
[f] from hospital patient records, of service use within site of index hospitalisation
[g] extracted from NHFD post recruitment window closing
