# Supplementary material for: PERFECTED enhanced recovery pathway (PERFECT-ER) versus standard acute hospital care for people after hip fracture surgery who have cognitive impairment: a feasibility cluster randomised controlled trial
Source: BMJ Open. 2022 Feb 28;12(2):e055267. doi: 10.1136/bmjopen-2021-055267 (PMC8886407; doi:10.1136/bmjopen-2021-055267)
Supplement: Supplementary data [file bmjopen-2021-055267supp002.pdf]

Supplementary Table 2. Per-site cost of 3 months start-up and 15 months of input from PERFECT-ER SIL AND PIL

**Per site**			
**SIL**	**% of year**	**Period FTE**	**Annual FTE**
Champion ERP 1st August to 31st October 2016	0.25	0.5	0.125
First year: 1/11/2016 - 31/7/2017	0.75	0.2	0.15
Second year: 1/8/2017 - 31/1/2018	0.5	0.2	0.1
Total FTE @£70,017 per annum (2016-17 prices)[a]	**£26,594**		
**PPL**	**Hours**		
First year: 1 hour/week for 3 months	13		
First year: 1 hour/month for 9 months	9		
Second year: 1 hour/month for 6 months	6		
Total hours PPL input	28		
Total hours @£106 per hour (2016-17 prices)[b]	**£2,968**		

[a]source: Schema 14: Hospital Nurses, AfC band 6[25]
[b]source: Schema 15. Hospital-based doctors, Medical Consultant[25]
