# Supplementary material for: PERFECTED enhanced recovery pathway (PERFECT-ER) versus standard acute hospital care for people after hip fracture surgery who have cognitive impairment: a feasibility cluster randomised controlled trial
Source: BMJ Open. 2022 Feb 28;12(2):e055267. doi: 10.1136/bmjopen-2021-055267 (PMC8886407; doi:10.1136/bmjopen-2021-055267)
Supplement: Supplementary data [file bmjopen-2021-055267supp003.pdf]

Supplementary Table 3: Hospital baseline characteristics

	**Intervention**			**Control** [a,b]		
	**Median**	**Max**	**Min**	**Median**	**Max**	**Min**
Number of Beds on Ward	27.0	41.0	15.0	28.0	38.0	25.0
Number of Bed Days on Ward in last 12 months	9855.0	14965.0	5475.0	10220.0	13870.0	9038.0
Occupied Bed Rate (%) in last 12 months	93.0	99.0	90.0	96.0	100	93.0
Number of Falls on Ward in last 12 months	42.0	82.0	25.0	60.0	111.0	32.0
Number of Deaths on Ward in last 12 months	30.0	66.0	7.0	34.0	68.0	13.0
Registered/Qualified Nurses	22.0	27.5	16.2	19.8	26.8	12.0
Geriatricians	1.0	2.6	0.5	1.0	1.0	0.8
Orthopaedic Surgeons	0.3	1.0	0.0	1.5	12.0	0.0
Other Consultants	0.0	0.4	0.0	0.0	4.7	0.0
Other Registrars	0.5	1.0	0.0	1.0	5.6	0.4
Other Junior Doctors	1.5	2.5	0.0	3.0	3.0	1.0

a One hospital (Control) missing all data
b One hospital (Control) missing data for *Number of Falls on the Ward* in last 12 months.
