# Supplementary material for: PERFECTED enhanced recovery pathway (PERFECT-ER) versus standard acute hospital care for people after hip fracture surgery who have cognitive impairment: a feasibility cluster randomised controlled trial
Source: BMJ Open. 2022 Feb 28;12(2):e055267. doi: 10.1136/bmjopen-2021-055267 (PMC8886407; doi:10.1136/bmjopen-2021-055267)
Supplement: Supplementary data [file bmjopen-2021-055267supp004.pdf]

Supplementary Table 4: Available data for analysis

Time point & outcome measure	Intervention (N = 132)	Control (N = 150)
**Baseline**		
HowRThey	5 (3.8)	13 (8.7)
HowRwe	39 (29.5)	56 (37.3)
EQ-5D – Patient	40 (30.3)	63 (42.0)
EQ-5D – SI	7 (5.3)	11 (7.3)
EQ-5D – Proxy	6 (4.5)	14 (9.3)
MMSE	4 (3.0)	13 (8.7)
BADLS	5 (3.8)	9 (6.0)
4AT	5 (3.8)	18 (12.0)
CDR	5 (3.8)	13 (8.7)
**Discharge**	**Expected = 123**	**Expected = 143**
HowRthey	116 (94.3)	116 (81.1)
HowRwe	84 (68.3)	72 (50.3)
4AT	116 (94.3)	103 (72.0)
Length of Stay	121 (98.4)	142 (99.3)
PERFECTER Score	122 (99.2)	141 (98.6)
**1 Month**	**Expected = 108**	**Expected = 122**
MMSE	106 (98.1)	111 (91.0)
BADLS	104 (96.3)	112 (91.8)
EQ-5D Patient	84 (77.8)	78 (63.9)
EQ-5D SI	106 (98.1)	110 (90.2)
EQ-5D Proxy	105 (97.2)	112 (91.8)
HowRthey	102 (94.4)	110 (90.2)
**3 Months**	**Expected = 83**	**Expected = 102**
MMSE	81 (97.6)	97 (95.1)
Timed Up & Go	44 (53.0)	50 (49.0)
BADLS	81 (97.6)	96 (94.1)
HowRthey	82 (98.8)	94 (92.2)
EQ-5D Patient	61 (73.5)	69 (67.6)
EQ-5D SI	81 (97.6)	97 (95.1)
EQ-5D Proxy	82 (98.8)	98 (96.1)
**6 Months**	**Expected = 64**	**Expected = 80**
MMSE	63 (98.4)	72 (90.0)
BADLS	61 (95.3)	77 (96.3)
HowRthey	64 (100)	76 (95.0)
EQ-5D Patient	36 (56.3)	43 (53.8)
EQ-5D SI	48 (75.0)	65 (81.3)
EQ-5D Proxy	44 (68.8)	65 (81.3)
Global CDR	64 (100)	66 (82.5)

a: Estimated as negative
