# Supplementary material for: PERFECTED enhanced recovery pathway (PERFECT-ER) versus standard acute hospital care for people after hip fracture surgery who have cognitive impairment: a feasibility cluster randomised controlled trial
Source: BMJ Open. 2022 Feb 28;12(2):e055267. doi: 10.1136/bmjopen-2021-055267 (PMC8886407; doi:10.1136/bmjopen-2021-055267)
Supplement: Supplementary data [file bmjopen-2021-055267supp005.pdf]

Supplementary Table 5. Per-site costs over the study period (1/11/2016 – 31/1/2018)

Site	Estimated total numbers of potentially affected patients[a]	SIL cost per case on study ward	PPL cost per case on study ward	Total costs per potentially affected patient
**01**	190	£140	£16	£156
**03**	205	£130	£14	£144
**06**	76	£350	£39	£389
**07**	61	£436	£49	£485
**10**	225	£118	£13	£131

[a]Patients on study wards, 60≥, with confusion (AMTS≤8/4AT≥), hip fracture, surgery for hip fracture, ward stay of≥ 5 days.
