# Supplementary material for: PERFECTED enhanced recovery pathway (PERFECT-ER) versus standard acute hospital care for people after hip fracture surgery who have cognitive impairment: a feasibility cluster randomised controlled trial
Source: BMJ Open. 2022 Feb 28;12(2):e055267. doi: 10.1136/bmjopen-2021-055267 (PMC8886407; doi:10.1136/bmjopen-2021-055267)
Supplement: Supplementary data [file bmjopen-2021-055267supp006.pdf]

Supplementary Table 6: Mean costs (standard errors): Health & social care services for participant, unpaid carer (SI) costs, out-of-pocket costs, total health & social care and societal costs over prior three months, at baseline and one-, three-, and six-month follow-ups (£, 2016-17)

Cost	Intervention (n=132)			Control (n=150)			Intervention-control	
	n	Mean	SE	n	Mean	SE	Mean difference	95% CI
**Baseline**								
Health & social care (HRE)	125	3740	709	135	3196	691	544	-1697, 2784
Health & social care (SIR)	123	3458	653	130	3148	642	310	-1761, 2381
Health & social care (SIR+)	125	3544	663	135	3094	645	450	-1642, 2543
Societal (HRE)[f]	95	9661	949	100	9783	932	-122	-3131, 2886
Societal (SIR)[f]	93	9249	946	97	9823	934	-574	-3581, 2433
Societal (SIR+)[f]	95	9299	886	100	9635	867	-336	-3140, 2469
**1 month**								
Intervn.+Health & social care (HRE)	89	12859	531	99	11636	509	1223	-441, 2886
Intervn.+Health & social care (SIR)	89	13890	980	95	11489	974	2401	-726, 5527
Intervn.+Health & social care (SIR+)	89	13894	945	99	11574	922	2320	-667, 5306
Intervn.+Societal (HRE)[f]	75	14191	526	80	13988	511	203	-1456, 1862
Intervn.+Societal (SIR)[f]	75	15032	1023	76	14123	1023	908	-2364, 4180
Intervn.+Societal (SIR+)[f]	75	15036	1023	80	14141	1000	895	-2341, 4131
**3 months**								
Intervn.+Health & social care (HRE)	75	9193	1721	88	5946	1684	3247	-2200, 8695
Intervn.+Health & social care (SIR)	75	8315	1258	87	4310	1226	4004*	30, 7979
Intervn.+Health & social care (SIR+)	75	8325	1274	88	4621	1236	3704	-311, 7719
Intervn.+Societal (HRE)[f]	64	12794	1909	71	10748	1846	2047	-3961, 8054
Intervn.+Societal (SIR)[f]	64	11983	1341	70	8923	1297	3060	-1161, 7281
Intervn.+Societal (SIR+)[f]	64	11995	1293	71	9243	1243	2752	-1305, 6808
**6 months**								
Intervn.+Health & social care (HRE)	57	6807	1402	64	5146	1413	1661	-2842, 6164
Intervn.+Health & social care (SIR)	57	6827	999	64	4308	965	2519	-624, 5661
Intervn.+Health & social care (SIR+)	57	6839	1004	64	4308	971	2531	-629, 5692
Intervn.+Societal (HRE)[f]	52	11511	1462	54	12478	1476	-967	-5666, 3733
Intervn.+Societal (SIR)[f]	52	11514	1506	54	11483	1536	31	-4836, 4897
Intervn.+Societal (SIR+)[f]	52	11528	1511	54	11483	1541	44	-4839, 4928

Note: NHS CC=NHS continuing care; HRE=health records extraction; SIR=Suitable Informant-reported; SIR+= corresponding hospital costs data from HRE used when costs were missing from the SIR dataset; Intervn.=Intervention costs

a Funded by NHS or Social Services

b Provided by NHS or Social Services

c expenditure by self or family on equipment purchases

d expenditure by self or family on travel to appointments

e unpaid carers' time in care and support to participant

f societal costs include: participant's health and social care costs; unpaid carers' time in care and support to participant; expenditure by self or family on travel to appointments, equipment purchases
