# Supplementary material for: PERFECTED enhanced recovery pathway (PERFECT-ER) versus standard acute hospital care for people after hip fracture surgery who have cognitive impairment: a feasibility cluster randomised controlled trial
Source: BMJ Open. 2022 Feb 28;12(2):e055267. doi: 10.1136/bmjopen-2021-055267 (PMC8886407; doi:10.1136/bmjopen-2021-055267)
Supplement: Supplementary data [file bmjopen-2021-055267supp007.pdf]

Supplementary Table 7. Mean six-month costs (excluding or including intervention costs) over the study period (£, 2016-16 prices). Sample: cases where total costs were available across follow-up assessments

**Costs**	**Intervention**			**Control**			**Intervention - Control**	
	**n**	**Mean**	**SE**	**n**	**Mean**	**SE**	**Mean difference**	**95% CI**
Health & social care (HRE)	47	25 414	2 227	56	21 164	2 142	4 250	-2 739, 11 239
Health & social care (SIR)	47	26 304	1 741	53	18 930	1 639	7 373*	1 964, 12 782
Health & social care (SIR+)	47	26 342	1 731	56	19 231	1 586	7 111*	1 800, 12 422
Societal (HRE)[a]	39	35 837	3 118	38	38 067	3 227	-2 230	-12 578, 8 118
Societal (SIR)[a]	39	36 478	3 104	36	35 104	3 325	1 374	-9 115, 118 63
Societal (SIR+)[a]	39	36 524	3 235	38	35 067	3 358	1 456	-9 295, 12 208
Intervn.+Health & social care (HRE)	47	25 677	2 251	56	21 164	2 172	4 513	-2 563, 11 588
Intervn.+Health & social care (SIR)	47	26 567	1 744	53	18 930	1 642	7 636*	2 217, 13 055
Intervn.+Health & social care (SIR+)	47	26 605	1 734	56	19 231	1 589	7 374*	2 053, 12 695
Intervn.+Societal (HRE)[a]	39	36 080	3 142	38	38 067	3 253	-1 987	-12 416, 8 442
Intervn.+Societal (SIR)[a]	39	36 721	3 127	36	35 104	3 350	1 618	-8 951, 12 186
Intervn.+Societal (SIR+)[a]	39	36 767	3 256	38	35 067	3 381	1 700	-9 124, 12 523

Note: NHS CC=NHS continuing care; HRE=health records extraction; SIR=Suitable Informant-reported; SIR+= corresponding hospital costs data from HRE used when costs were missing from the SIR dataset; Intervn.=Intervention costs

* $p<0.05$

a. societal costs include: participant’s health and social care costs; unpaid carers’ time in care and support to participant; expenditure by self or family on travel to appointments, equipment purchases
