# Supplementary material for: PERFECTED enhanced recovery pathway (PERFECT-ER) versus standard acute hospital care for people after hip fracture surgery who have cognitive impairment: a feasibility cluster randomised controlled trial
Source: BMJ Open. 2022 Feb 28;12(2):e055267. doi: 10.1136/bmjopen-2021-055267 (PMC8886407; doi:10.1136/bmjopen-2021-055267)
Supplement: Supplementary data [file bmjopen-2021-055267supp008.pdf]

Supplementary Table 8. Intra-class correlations of 6-month total health and social care and societal costs (£,2016-17) and QALY over 6 months. Sample: cases where costs or outcomes data were available at all study period time points

	Intervention (n=132) (N=5)				Control (n=150) (N=6)			
	**n**	**N**	**Mean**	**95% CI**	**n**	**N**	**Mean**	**95% CI**
Costs								
Health & social care (HRE)[d]	47	5	-0.045	-0.148 to 0.057	56	6	0.117	-0.152 to 0.386
Health & social care (SIR)[d]	47	5	-0.051	-0.147 to 0.045	53	6	0.034	-0.165 to 0.232
Health & social care (SIR+)[d]	47	5	-0.050	-0.147 to 0.048	56	6	0.028	-0.154 to 0.210
Societal (HRE)[g]	39	5	-0.041	-0.194 to 0.112	38	5	0.190	-0.189 to 0.569
Societal (SIR)[g]	39	5	-0.057	-0.194 to 0.079	36	5	0.214	-0.201 to 0.628
Societal (SIR+)[g]	39	5	-0.055	-0.194 to 0.084	38	5	0.240	-0.169 to 0.649
Intervention + Health & social care (HRE)[d]	47	5	-0.039	-0.149 to 0.071	56	6	0.117	-0.152 to 0.386
Intervention + Health & social care (SIR)[d]	47	5	-0.044	-0.148 to 0.059	53	6	0.033	-0.165 to 0.232
Intervention + Health & social care (SIR+)[d]	47	5	-0.043	-0.148 to 0.061	56	6	0.028	-0.154 to 0.210
Intervention +Societal (HRE)[g]	39	5	-0.033	-0.195 to 0.128	38	5	0.190	-0.189 to 0.569
Intervention +Societal (SIR)[g]	39	5	-0.049	-0.194 to 0.096	36	5	0.214	-0.201 to 0.628
Intervention +Societal (SIR+)[g]	39	5	-0.047	-0.194 to 0.101	38	5	0.240	-0.169 to 0.649
QALY								
Participant 6-month QALY (EQ-5D-5L)	30	5	0.268	-0.173 to 0.710	31	4	0.263	-0.236 to 0.762
Participant 6-month QALY (EQ-5D-5L-Proxy)	42	5	0.068	-0.181 to 0.316	62	6	0.110	-0.136 to 0.355
Participant 6-month QALY (DEMQOL-U)	34	5	0.236	-0.190 to 0.662	34	5	-0.001	-0.255 to 0.253
Participant 6-month QALY (DEMQOL-PROXY)	60	5	0.004	-0.121 to 0.129	67	6	0.037	-0.125 to 0.198
SI 6-month QALY (EQ-5D-5L)	48	5	0.255	-0.109 to 0.619	63	6	-0.040	-0.135 to 0.055

Note: HRE=health records extraction; SIR=Suitable Informant-reported; SIR+= hospital costs data from HRE used when these costs were missing from SIR dataset; SI=suitable informant; n=number of observations; N=number of clusters
