# Supplementary material for: PERFECTED enhanced recovery pathway (PERFECT-ER) versus standard acute hospital care for people after hip fracture surgery who have cognitive impairment: a feasibility cluster randomised controlled trial
Source: BMJ Open. 2022 Feb 28;12(2):e055267. doi: 10.1136/bmjopen-2021-055267 (PMC8886407; doi:10.1136/bmjopen-2021-055267)
Supplement: Supplementary data [file bmjopen-2021-055267supp009.pdf]

Supplementary Table 9. Agreement between hospital records-extracted and self-report hospital service use and costs.

Item	Period	Mean Difference (SD) (HRE – SIR)	ρ_c (95% CI)	95% limits of agreement	Exact (none) % (N)	Exact (some) % (N)	Under % (N)	Over % (N)
**A&E visits**	Time 0	-0.339 (2.945)	0.099 (0.061, 0.136)	-6.110, 5.433	77 (198)	9 (23)	4 (10)	10 (26)
	Time 1	-0.015 (0.304)	0.452 (0.343, 0.561)	-0.611, 0.581	90 (186)	3 (7)	2 (5)	4 (8)
	Time 2	-0.124 (0.908)	0.308 (0.218, 0.397)	-1.903, 1.655	78 (132)	8 (14)	5 (8)	9 (15)
	Time 3	-0.143 (0.817)	0.367 (0.249, 0.485)	-1.744, 1.458	75 (95)	15 (19)	2 (2)	8 (10)
**Admissions**	Time 0	0.100 (0.630)	0.620 (0.462, 0.777)	-1.134, 1.334	38 (23)	27 (16)	22 (13)	13 (8)
	Time 1	0.108 (0.350)	0.454 (0.350, 0.557)	-0.577, 0.794	-	90 (75)	10 (8)	-
	Time 2	0.061 (0.493)	0.617 (0.523, 0.711)	-0.905, 1.028	69 (112)	9 (14)	14 (23)	9 (14)
	Time 3	0.033 (0.284)	0.813 (0.753, 0.873)	-0.525, 0.590	83 (100)	8 (10)	6 (7)	3 (3)
**Inpatient days**	Time 0	0.508 (5.513)	0.449 (0.359, 0.540)	-10.298, 11.313	84 (103)	8 (10)	6 (7)	2 (3)
	Time 1	0.000 (8.028)	0.544 (0.445, 0.643)	-15.735, 15.735	-	41 (81)	15 (29)	44 (86)
	Time 2	1.093 (11.281)	0.460 (0.342, 0.579)	-21.017, 23.203	66 (107)	2 (3)	15 (24)	17 (27)
	Time 3	1.293 (9.211)	0.197 (0.082, 0.311)	-16.759, 19.346	87 (100)	1 (1)	9 (10)	3 (4)
**Day hospital**	Time 0	0.031 (0.902)	0.037 (-0.075, 0.149)	-1.736, 1.799	94 (238)	-	5 (12)	2 (4)
	Time 1	0.025 (0.221)	-	-0.408, 0.457	99 (161)	-	1 (2)	-
	Time 2	0.006 (0.132)	0.724 (0.670, 0.777)	-0.254, 0.265	98 (169)	-	1 (2)	1 (1)
	Time 3	0.056 (0.319)	0.428 (0.369, 0.487)	-0.569, 0.681	97 (121)	-	3 (4)	-
**Outpatient**	Time 0	0.008 (1.069)	0.537 (0.448 to 0.625)	-2.087 to 2.103	67 (164)	11 (28)	11 (26)	11 (28)
	Time 1	-0.015 (0.272)	0.417 (0.303 to 0.530)	-0.548 to 0.519	93 (188)	3 (6)	1 (3)	3 (6)
	Time 2	-0.047 (0.554)	0.529 (0.420 to 0.637)	-1.134 to 1.039	77 (130)	11 (18)	4 (6)	9 (15)
	Time 3	0.016 (0.589)	0.764 (0.691 to 0.836)	-1.138 to 1.171	72 (88)	10 (12)	8 (10)	10 (12)
**Hospital costs**	Time 0	177.437 (1654.363)	0.660 (0.597 to 0.723)	-3 065 to 3 420	50 (130)	5 (12)	24 (62)	21 (55)
	Time 1	-420.340 (3 355.633)	0.379 (0.262 to 0.496)	-6 997 to 6 157	-	27 (55)	17 (34)	56 (112)
	Time 2	1 336.827 (4 773.868)	0.295 (0.182 to 0.409)	-8 020 to 10 693	45 (78)	2 (3)	33 (57)	21 (36)
	Time 3	342.110 (3 151.993)	0.261 (0.136 to 0.385)	-5 836 to 6 520	52 (66)	3 (4)	24 (31)	21 (27)

Notes: HRE=extraction from hospital records; SIR=Suitable Informant report; Time 0=3 months prior to baseline assessment; Time 1=1 month post-fracture; Time 2=2 months prior to 3 months post-fracture; Time 3=3 months prior to 6 months post-fracture; ρ_c=Lin's concordance correlation coefficient; Exact(none)=zero use/costs in both sources; Exact (some)=the same frequency or cost in both sources; Under=under-reporting (lower frequency/cost in SIR than HRE); Over=over-reporting (higher frequency/use in SIR than HRE).
