# Supplementary material for: PERFECTED enhanced recovery pathway (PERFECT-ER) versus standard acute hospital care for people after hip fracture surgery who have cognitive impairment: a feasibility cluster randomised controlled trial
Source: BMJ Open. 2022 Feb 28;12(2):e055267. doi: 10.1136/bmjopen-2021-055267 (PMC8886407; doi:10.1136/bmjopen-2021-055267)
Supplement: Author's manuscript [file bmjopen-2021-055267.draft_revisions.pdf]

BMJ Open is committed to open peer review. As part of this commitment we make the peer review history of every article we publish publicly available.

When an article is published we post the peer reviewers' comments and the authors' responses online. We also post the versions of the paper that were used during peer review. These are the versions that the peer review comments apply to.

The versions of the paper that follow are the versions that were submitted during the peer review process. They are not the versions of record or the final published versions. They should not be cited or distributed as the published version of this manuscript.

BMJ Open is an open access journal and the full, final, typeset and author-corrected version of record of the manuscript is available on our site with no access controls, subscription charges or pay-per-view fees (http://bmjopen.bmj.com).

If you have any questions on BMJ Open's open peer review process please email info.bmjopen@bmj.com

BMJ Open

PERFECTED enhanced recovery pathway (PERFECT-ER) versus standard acute hospital care for people after hip fracture surgery who have cognitive impairment: a feasibility cluster randomised controlled trial

Journal:	*BMJ Open*
Manuscript ID	bmjopen-2021-055267
Article Type:	Original research
Date Submitted by the Author:	12-Jul-2021
Complete List of Authors:	cross, jane; University of East Anglia, Faculty of Medicine and Health Sciences<br>Hamond, Simon; University of East Anglia, Norwich Medical School<br>Shepstone, Lee; University of East Anglia, Norwich Medical School<br>Poland, Fiona; University of East Anglia, School of Health Sciences<br>Henderson, Catherine; London Business School Department of Economics, Care Policy<br>Backhouse, Tamara; University of East Anglia, Norwich Medical School<br>Penhale, Bridget; University of East Anglia, School of Health Sciences<br>Donell, Simon; Norfolk and Norwich University Hospital<br>Knapp, Martin; Norfolk and Norwich University Hospital<br>Lewins, Douglas; University of East Anglia, Norwich Medical School<br>MacLullich, Alasdair; University of Edinburgh Western General Hospital, Geriatric Medicine Unit<br>Patel, Martyn; Norfolk and Norwich University Hospitals NHS Foundation Trust<br>Sahota, Opinder; Nottingham University Hospitals NHS Trust, HCOP<br>Smith, Toby; University of East Anglia<br>Waring, Justin ; University of Birmingham<br>Howard, R; King's College London<br>Ballard, Clive ; University of Exeter, College of Medicine and Health<br>Fox, Chris; University of East Anglia, Department of Psychological Sciences
Keywords:	GERIATRIC MEDICINE, Rehabilitation medicine < INTERNAL MEDICINE, Hip < ORTHOPAEDIC & TRAUMA SURGERY, Delirium & cognitive disorders < PSYCHIATRY, REHABILITATION MEDICINE

SCHOLARONE™
Manuscripts

I, the Submitting Author has the right to grant and does grant on behalf of all authors of the Work (as defined in the below author licence), an exclusive licence and/or a non-exclusive licence for contributions from authors who are: i) UK Crown employees; ii) where BMJ has agreed a CC-BY licence shall apply, and/or iii) in accordance with the terms applicable for US Federal Government officers or employees acting as part of their official duties; on a worldwide, perpetual, irrevocable, royalty-free basis to BMJ Publishing Group Ltd ("BMJ") its licensees and where the relevant Journal is co-owned by BMJ to the co-owners of the Journal, to publish the Work in this journal and any other BMJ products and to exploit all rights, as set out in our licence.

The Submitting Author accepts and understands that any supply made under these terms is made by BMJ to the Submitting Author unless you are acting as an employee on behalf of your employer or a postgraduate student of an affiliated institution which is paying any applicable article publishing charge ("APC") for Open Access articles. Where the Submitting Author wishes to make the Work available on an Open Access basis (and intends to pay the relevant APC), the terms of reuse of such Open Access shall be governed by a Creative Commons licence – details of these licences and which Creative Commons licence will apply to this Work are set out in our licence referred to above.

Other than as permitted in any relevant BMJ Author's Self Archiving Policies, I confirm this Work has not been accepted for publication elsewhere, is not being considered for publication elsewhere and does not duplicate material already published. I confirm all authors consent to publication of this Work and authorise the granting of this licence.

For peer review only

BMJ OPEN

TITLE PAGE

Title:

PERFECTED enhanced recovery pathway (PERFECT-ER) versus standard acute hospital care for people after hip fracture surgery who have cognitive impairment: a feasibility cluster randomised controlled trial

Authors and Affiliations:

Jane L Cross[1] Simon P Hammond[2] Lee Shepstone[3] Fiona Poland[1] Catherine Henderson[4] Tamara Backhouse[1] Bridget Penhale[1] Simon Donell[5] Martin Knapp[4] Douglas Lewins[6] Alasdair MacLullich[7] Martyn Patel[5] Opinder Sahota[8] Toby O Smith[1] Justin Waring[9] Robert Howard[10] Clive Ballard[11] Chris Fox[3*]

1 School of Health Sciences, University of East Anglia, Norwich Research Park, Norwich, UK
2 School of Education & Lifelong Learning, University of East Anglia, Norwich Research Park, UK
3 Norwich Medical School, University of East Anglia, Norwich Research Park, Norwich, UK
4 Care Policy and Evaluation Centre (CPEC), London School of Economics, London, UK
5 Norfolk and Norwich University Hospitals NHS Foundation Trust, Norwich, UK
6 Thornfield House, Gosberton, Spalding, UK

For peer review only - http://bmjopen.bmj.com/site/about/guidelines.xhtml

7 Geriatric Medicine, University of Edinburgh, Edinburgh, UK

8 Nottingham University Hospitals NHS Trust, Queens Medical Centre Campus, Nottingham, UK

9 Health Services Management Centre, University of Birmingham, Birmingham, UK

10 King's College London, Uk

11 College of Medicine and Health, University of Exeter, UK

Corresponding author contact details:

Prof Chris Fox

Email: Chris.Fox@uea.ac.uk

Key words: Cognitive impairment, Dementia, hip fracture, Enhanced Recovery Pathway, acute hospital, cluster randomised controlled trial

Manuscript 3801 words

Abstract 377 words

Tables 4

Figures 1

Supplementary tables 4

For peer review only

For peer review only - http://bmjopen.bmj.com/site/about/guidelines.xhtml

ABSTRACT

Objectives: Assess the feasibility of a cluster RCT with economic analysis to measure the clinical and cost-effectiveness of an Enhanced Recovery Pathway for people with hip fracture and Cognitive Impairment (CI).

Setting: Eleven acute hospitals sampled accross three different UK regions.

Participants: 284 participants (208 female: 69 male). Inclusion criteria: aged >60 years, confirmed proximal hip fracture requiring surgical fixation and CI; pre-operative AMTS ≤8 in England or a 4AT score ≥1 in Scotland; minimum of five days in study ward; a "suitable informant" able to provide proxy measures, recruited within seven days of hip fracture surgery. Exclusion critera: did not undergo hip surgery; not expected to survive beyond four weeks; already enrolled in a clinical trial.

Intervention: PERFECT-ER, an enhanced recovery pathway with 15 quality targets supported by a checklist and manual, a Service Improvement Lead (SIL) a Process Lead (PPL) and implemented using a Plan Do Act model.

Primary and Secondary outcome measures: Feasibility outcomes: recruitment and attrition, intervention acceptability and fidelity, completion of participant reported outcome measures, preliminary estimates of potential effectiveness using mortality, EQ-5D-5L, economic and clinical outcome scores.

Results: 284 participants were consented and recruited (132 PEFECT-ER Intervention). Mean recruitment rates were the same in intervention and control sites, (range: 1.2 and 2.7 participants per month). At three months a relatively small effect (one quarter of a standard deviation) on health-related quality of life of the patient measured with EQ-5D-5L by proxy in the intervention group.

Conclusion: This trial design was feasible with modifications to recruitment. Mechanisms for delivering consistency in the PERFECT-ER intervention and participant retention need to

For peer review only - http://bmjopen.bmj.com/site/about/guidelines.xhtml

be addressed. However, an RCT may not be the optimal research design to evaluate this perioperative intervention due to the complexity of caring for people with cognitive impairment after hip fracture.

Trial registration: PERFECTED CRCT ISRCTN99336264

Strengths and Limitations

- This feasibility RCT tested the PERFECT-ER intervention across a number of different UK NHS organisations and provides valuable evidence that the PERFECT-ER intervention and trial design can be delivered in different settings, providing assurance for a future trial which would require a substantially larger number of trials sites.
- The study builds on previous work which attempts to clarify the complex associations between hospitalisation, pre-admission cognitive impairment, post-admission cognitive impairment, functional decline and mortality.
- Only a small proportion of people of non-white ethnicity were recruited (patients and suitable informants) it therefore remains unclear how successful the approach, consent and retention of trial participants would be for those from wider cultural, ethnic or social backgrounds.
- The duration and type of cognitive impairment i.e. established dementia versus temporary delirium, was not controlled within the eligibility criteria but this may be an important factor which should be considered in any future RCT where a more suitably powered secondary analyses may be possible to explore the impact of the PERFECT-ER intervention based on a participant's presenting cognitive impairment.

For peer review only - http://bmjopen.bmj.com/site/about/guidelines.xhtml

INTRODUCTION

Hip fracture is associated with advancing frailty and has substantial impact on the health, well-being and independence of older people and their families (1, 2) Acute hip fracture care costs an estimated £1.1 billion per annum in the UK (3). In the 12 months after fracture, patients are at increased risk of cognitive and functional decline, admission to long-term care institutions and higher mortality (4). People with cognitive impairment (CI) are amongst the most vulnerable in acute hospital settings (5), with lower short-term survival and 24% mortality during admission (4). They are susceptible to suboptimal and inconsistent care standards that contribute to cognitive deterioration, increase risk of post-operative complications, prolong length of stay and cause loss of independence (6).

In older adults with hip fracture, approximately 19% have dementia and up to 42% some degree of CI that may not meet criteria for a dementia diagnosis (7). People with hip fracture and CI are frequently cared for in environments which deliver excellent hip fracture care but are less skilled managing people with CI (8, 9). Hospital care of patients with CI remains an ongoing area of concern (5) with systemic failures in the care of older people repeatedly identified (10). Hospital staff may lack the knowledge and skills necessary to identify and assess CI, leading to under-identification which negatively affects access to rehabilitation services, supported discharge planning, person-centred care plans and involvement of families and carers (11-14).

This study assessed the feasibility of a cluster design randomised controlled trial (RCT) to measure the clinical and cost-effectiveness of an Enhanced Recovery Pathway versus

For peer review only - http://bmjopen.bmj.com/site/about/guidelines.xhtml

standard care in acute hospitals for people after hip fracture surgery who demonstrate CI. Feasibility objectives included recruitment, retention, outcome selection, sample size estimation and acceptability of intervention training and delivery in NHS services.

METHODS

This paper has been prepared in accordance with the CONSORT Extension for Pilot and Feasibility Studies (15) reporting guideline. The study methods are summarised below and previously reported in detail (16).

Public and Patient Involvement

Patients and the pubic were involved from the conception of this study, through the review and funding process, the study, analysis and writing the findings. They were part of the steering, oversight and data monitoring groups.

Design and setting

A multi-centre, feasibility, cluster RCT was undertaken. In line with MRC guidance for complex interventions, an integrated process evaluation was conducted (17); this is currently under review.

Randomisation

Randomisation was stratified by geographical area, with one intervention and one control hospital in UK region. Eleven NHS hospitals were randomised to deliver experimental

For peer review only - http://bmjopen.bmj.com/site/about/guidelines.xhtml

(PERFECT-ER) or control interventions. Recruitment was between November 2016 to February 2018.

Participants

Inclusion criteria

Participants were included if:

- Confirmed proximal hip fracture requiring surgery.
- Aged 60 years or over at the time of surgery.
- Pre-operative AMTS ≤8 in England (including those with zero because of an inability to answer questions) or a 4AT score ≥1 in Scotland.
- Minimum of five days in the study ward.
- Patient had a “suitable informant” (e.g. relative, unpaid or paid carer, care home manager) with a minimum of once a month face-to-face or telephone contact with the patient and able to provide proxy measures where required.
- Both patient and suitable informant to be recruited into the trial within seven days of the hip fracture surgery.

Exclusion criteria

Participants were excluded if:

- Did not undergo hip surgery.
- Patient not expected to survive beyond four weeks.
- Patient already enrolled in a clinical trial of an investigational medicinal product.

Sample Size

The target sample was 400 patient participants (200 per arm) from 10 centres (40 patient participants per site), based on the degree of precision for the estimated intra-class correlation

For peer review only - http://bmjopen.bmj.com/site/about/guidelines.xhtml

coefficients (ICC). This was expected to provide a standard error for the ICC of between 0.033 and 0.041, for a true ICC value of between 0.05 and 0.10 for any endpoint. A priori, it was expected that four participants would be recruited per site, per month, over 10 months recruitment period.

Participant recruitment and consent

A three-step recruitment process was implemented, guided by previous phases of the PERFECTED programme, previous studies (18, 19) and input from clinical and academic collaborators:

1. Research nurses identified all new hip fracture admissions and screened for pre-recruitment eligibility in collaboration with clinical staff.
2. Patients (and where possible their potential suitable informant) were approached by the research nurse who provided study information as soon as clinically appropriate. Mental capacity was assessed by the research nurse, according to the appropriate legislative frameworks. In those lacking capacity to consent, consultee agreement from a relative or professional caregiver was sought, following the requirements of UK capacity legislation (20, 21).
3. The research nurse approached the patient and suitable informant to obtain informed consent.

Intervention

Experimental intervention: PERFECT-ER

For peer review only - http://bmjopen.bmj.com/site/about/guidelines.xhtml

The PERFECT-ER is a multi-component intervention, implemented using service improvement principles, comprising:

- The PERFECT-ER checklist and manual.
- A Service Improve Lead (SIL) and PERFECTED Process Lead (PPL).
- A model for change (Plan-Do-Study-Act) (22).

The checklist has 15 organisational items, and 68 individual patient items grouped into three stages (Admission and Pre-Operative; Post-Operative and Rehabilitation; and Discharge), reflecting the patient journey through acute care settings. It was designed to identify areas of strength, and potential for improvement in practice, and overarches current hip fracture guidance. A comprehensive handbook explaining how to implement and use the intervention (the PERFECT-ER manual) was provided.

In the three months prior to recruitment commencing, the intervention was implemented in intervention sites by the SIL working 0.50 FTE, following the handbook and adherence assessed. When sites commenced recruitment, SIL resource was reduced to 0.2 FTE for the study period. A senior clinician (PPL) assisted the SILs for an hour a week to implement PERFECT-ER then an hour per month during recruitment.

Comparator group

The control group received treatment as usual. What this consisted of was recorded to determine local practice.

For peer review only - http://bmjopen.bmj.com/site/about/guidelines.xhtml

Outcomes

Data were collected from medical records of participating hospitals, the National Hip Fracture Database (NHFD) (23) and participants and suitable informants (summarised in **Supplementary Table 1)**. Study feasibility outcome measures included: recruitment and attrition, intervention acceptability and fidelity, completion of participant reported outcome measures. The delivery of the intervention was monitored by auditing the patients notes against the PERFECT-ER checklist. Five patients per site were audited at the beginning of each implementation cycle and at the end of the trial: at three months pre-trial, 1.5 months pre-trial, trial baseline, four months, seven months, 10 months, 13 months, and 15 months. Clinical outcomes: mortality rate at 30 and 120 days; hospital admissions (number, length of stay and time to first admission); falls and mortality during previous six months; and the number of medications. Economic measures: Bristol Activities of Daily Living Scalre (BADLS), quality-adjusted life years (QALY) of the participant (1) computed from DEMQOL-U and DEMQOL-PROXY-U) and (2) computed from EQ-5D-5L completed by participants and again by proxy, QALY of the suitable informant (unpaid carer), use of health, social and unpaid care collected via the Client Services Receipt Inventory (CSRI) (24) and hospital service use abstracted from hospital records. Costs of the intervention were assembled from time inputs of personnel providing PERFECT-ER, including time spent championing the ERP in study set up. Costs of inputs per site were calculated by dividing the costs of each role by the number of potentially affected patients on each study ward over the intervention period. Unit costs for other services were from published sources (25-28).

Statistical Analysis

Clinical outcome analysis

For peer review only - http://bmjopen.bmj.com/site/about/guidelines.xhtml

The data analyses summarise study process information including recruitment, participant ‘flow’ and retention, sample characteristics and completeness of baseline and follow-up outcome measures. To assess fidelity of the intervention the mean ‘PERFECT-ER’ score of enacted checklist items was determined.

For each outcome measure, at each follow-up point, an intra-class correlation coefficient (ICC) was calculated together with a 95% confidence interval based on Searle’s method (29). These were calculated to assist the choice of primary outcome measure and inform potential sample size calculations for a definitive trial.

A precise estimate of intervention efficacy was not a primary objective of the data analyses. However, all efficacy outcome measures were modelled using a general linear model including the baseline value of the outcome (where available) and the treatment arm. Generalised Estimating Equations were used to account for ‘clustering’ created by the hospital level randomisation, thus accounting for the lack of independence of patient-level data within individual hospitals. The estimates of between arm difference are provided with 95% confidence intervals (CI). The relationship between the individual ‘PERFECT-ER’ score and outcomes were considered and a Pearson correlation coefficient calculated to assess the strength of the linear relationship. The difference in mean ‘PERFECT-ER’ score between those known to have died during the study and those known to have survived was also calculated.

Economic analysis

The economic evaluation took an NHS and Personal Social Services (social care) perspective and a societal perspective, incorporating costs of unpaid care and out-of-pocket expenses (for equipment, adaptations, travel to healthcare appointments).

For peer review only - http://bmjopen.bmj.com/site/about/guidelines.xhtml

We examined the ICC of quality-adjusted life year (QALY) and total costs at six-month follow-up, with Searle's confidence intervals (using the arithmetic mean cluster size for unbalanced data) derived from one-way analysis of variance (29).

- Incremental cost per 3.5 unit change in BADLs score of the participant (30)
- Incremental cost per QALY of the participant, (1) computed from DEMQOL-U; and (2) computed from EQ-5D-5L, completed by participants and again by proxy.

We computed utilities (to subsequently calculate QALYs) using societal weights (DEMQOL-U from the DEMQOL; DEMQOL-Proxy-U from the DEMQOL-Proxy; and EQ-5D-5L (31, 32). QALYs over the intervention period were derived using the trapezoid method to approximate the area under the quality of life curve, with linear interpolation between time-points.

We examined the extent to which hospital services use data from hospital records gave the same estimates as data collected by SIL-report. We examined the level of agreement on frequency of service use (counts) and total hospital costs between the two sources as estimated by Lin's concordance correlation coefficient (33). We also examined agreement between sources using the 95% limits of agreement approach (34), which calculates means and standard deviations of paired differences and the confidence interval for the difference, conditional on those differences being normally distributed and independent of the measures' magnitudes (35). Research nurses recorded the time taken to complete sections of the PERFECT-ER case report forms, covering multiple instruments/questions. To calculate a time-per-question estimate, the time taken to complete the CSRI, hospital use and medications review questions

For peer review only - http://bmjopen.bmj.com/site/about/guidelines.xhtml

was divided by the number of items in the respective sections. Time taken to complete the measures was calculated by multiplying the total number of questions by the time-per-question.

RESULTS

Participant recruitment and retention

Figure 1 illustrates patient flow. Recruitment rate by centre is presented in **Table 1**. Hospital characteristics at baseline are described in **Supplementary Table 2** which shows sites in both intervention and control groups are broadly similar. 282 participants, 132 from intervention sites and 150 from control, were recruited. There were 151 months of site recruitment, 70 in intervention and 81 in control sites. Average recruitment rates did not differ between intervention and control sites, ranging from 1.2 to 2.7 participants/month. Mean recruitment rate was 1.87 per site/month. This contrasts with the expected four per site/month. The demographic characteristics of the 282 study participants and suitable informant characteristics are shown in **Table 2.**

Figure 1 here

Table 1: Recruitment Rates by Centre

Group	Site	Start Date	Months	Recruited	Rate / Month
**Intervention**			70	132	1.9
	01	December 2016	14	26	1.9
	03	November 2016	15	34	2.3
	06	November 2016	15	30	2.0
	07	February 2017	12	19	1.6
	10	December 2016	14	23	1.6
**Control**			81	150	1.9
	02	November 2016	15	24	1.6
	04	November 2016	15	18	1.2
	05	November 2016	15	23	1.5

For peer review only - http://bmjopen.bmj.com/site/about/guidelines.xhtml

	08	November 2016	15	35	2.3
	09	November 2016	15	40	2.7
	50	July 2017	6	10	1.7
**Total**			151	282	1.87

Table 2: Participant and suitable informant baseline characteristics

**Participant Characteristic**	**Intervention (N = 132)**	**Control (N = 150)**	**Total (N = 282)**
Consent:			
Providing Own Consent	23 (17.6%)	38 (25.9%)	61 (21.9%)
Consultee / Legal Rep Consent	109 (82.4%)	112 (74.1%)	221 (78.1%)
Age (Mean (SD))	85.5 (7.4)	86.4 (7.9)	86.0 (7.6)
Missing	2	3	5
Gender:			
Male	37 (28.0%)	32 (22.1%)	69 (24.9%)
Female	95 (72.0%)	113 (77.9%)	208 (75.1%)
Missing	0	5	5
Ethnicity:			
Asian	1 (0.8%)	5 (3.4%)	6 (2.2%)
Black	1 (0.8%)	0	1 (0.4%)
White	106 (80.9%)	118 (80.8%)	224 (80.9%)
Unable to Respond	23 (17.6%)	23 (15.8%)	46 (16.6%)
Missing	1	4	5
Status:			
Married / Partner	40 (30.5%)	48 (32.7%)	88 (31.7%)
Divorced	7 (5.3%)	8 (5.4%)	15 (5.4%)
Single	6 (4.6%)	4 (2.7%)	10 (3.6%)
Widowed	54 (41.2%)	60 (40.8%)	114 (41.0%)
Unable to respond	24 (18.3%)	27 (18.4%)	51 (18.3%)
Missing	1	3	4
Employment Status:			
Employed	3 (2.3%)	3 (2.1%)	6 (2.2%)
Unemployed	3 (2.3%)	3 (2.1%)	6 (2.2%)
Retired	98 (74.8%)	107 (73.3%)	205 (74.0%)
Unable to respond	27 (20.6%)	33 (22.6%)	60 (21.7%)
Missing	1	4	5

**Suitable Informant Characteristic**	**Intervention (N = 132)**	**Control (N = 150)**	**Total (N = 282)**
Contact:			
Face-to-face	121 (91.7%)	129 (90.8%)	250 (91.2%)
Phone call	8 (6.1%)	11 (7.7%)	19 (6.9%)
Postal	3 (2.3%)	2 (1.4%)	5 (1.8%)
Missing	0	8	8
Relationship:			
Spouse	26 (19.8%)	26 (18.3%)	52 (19.0%)
Other Family Member	98 (74.8%)	110 (77.5%)	208 (76.2%)

For peer review only - http://bmjopen.bmj.com/site/about/guidelines.xhtml

For peer review only

Non-Family Member	4 (3.1%)	4 (2.8%)	8 (2.9%)
Paid Carer	3 (2.3%)	2 (1.4%)	5 (1.8%)
Missing	1	8	9
Age (Mean (SD))	60.7 (13.1)	62.2 (12.6)	61.5 (12.9)
Missing	4	10	14
Gender:			
Male	46 (34.8%)	63 (44.4%)	109 (39.8%)
Female	86 (65.2%)	79 (55.6%)	165 (60.2%)
Missing	0	8	8
Ethnicity:			
Asian	1 (0.8%)	7 (4.9%)	8 (2.9%)
Black	2 (1.5%)	0	2 (0.7%)
White	129 (97.7%)	135 (95.1%)	264 (96.4%)
Missing	0	8	8
Status:			
Married / Partner	98 (77.2%)	109 (77.3%)	207 (77.2%)
Divorced	11 (8.7%)	8 (5.7%)	19 (7.1%)
Single	15 (11.8%)	16 (11.3%)	31 (11.6%)
Widowed	3 (2.4%)	8 (5.7%)	11 (4.1%)
Missing	5	9	14
Employment Status:			
Employed	63 (48.1%)	54 (38.0%)	117 (42.9%)
Unemployed	11 (8.4%)	21 (14.8%)	32 (11.7%)
	57 (43.5%)	67 (47.2%)	124 (45.4%)
Retired	1	8	9
Missing			

Overall, the attrition rate was 50.7% (143/282). For the PERFECT-ER intervention attrition was 48.5% (64/132) and for control 52.7% (79/150).

Intervention Delivery

Although implementation was standardised across sites overall compliance with the intervention fluctuated over time and between sites. This is explored fully in the process evaluation (under review).

Missing Data

For peer review only - http://bmjopen.bmj.com/site/about/guidelines.xhtml

For peer review only

The degree of missing data varied across measures and across time-points. For example, baseline data collection consistently demonstrated high missingness for all outcomes (**Supplementary Table 3**). In contract, at discharge onwards, there were low missingness with the exception of the HowRwe at discharge EQ-5D Patient at one, three and six months, and the Timed Up and Go at three months. The EQ-5D for the suitable informant and proxy both demonstrated high missingness at six months in the intervention group (**Supplementary Table 3**).

Economic Outcome Selection

For economic data collection, there was relatively low occurrence of missing data for all health utilisation variables in primary care (6% to 8%) and hospital care, including both suitable informant-reported and hospital records-extracted use of emergency department, inpatient and outpatient services (4%-13%). Of a maximum of 23 medications reported, three to four costs were missing per case across the time points. More data were missing for suitable informant-reported unpaid care and lost working time. This was primarily because research nurses did not indicate whether the suitable informant was an unpaid or paid carer in 25% of cases at baseline and 17%, 15% and 13% of cases at one, three- and six-months follow-up respectively. Where the suitable informant was identified as an unpaid carer, rates of missingness in the unpaid carer questions were between 2% and 8% at the first three time points and 2% to 11% at six-month follow-up.

Clinical Outcome Feasibility

For peer review only - http://bmjopen.bmj.com/site/about/guidelines.xhtml

For peer review only

The baseline characteristics and outcomes are presented in **Tables 3** and **4**.

Table 3: Estimates of outcome

Time point & outcome measure	Intervention (N = 132) Mean (SD)	Control (N = 150) Mean (SD)	Adjusted difference [a]	95% Confidence Interval	p-value
**Baseline**					
HowRThey	4.96 (2.87)	4.55 (3.20)			
HowRwe	8.76 (2.38)	9.11 (2.23)			
EQ-5D – Patient	0.24 (0.37)	0.32 (0.36)			
EQ-5D – SI	0.80 (0.24)	0.85 (0.23)			
EQ-5D – Proxy	-0.01 (0.23)	0.15 (0.33)			
MMSE	12.2 (8.0)	10.8 (8.8)			
BADLS	24.3 (14.0)	21.0 (14.7)			
4AT	4.02 (3.33)	4.80 (4.02)			
CDR	1.63 (0.98)	1.41 (0.95)			
**Discharge**					
4AT	3.1 (2.7)	3.9 (3.4)	-0.45	(-1.23, 0.33)	0.255
HowRThey	3.3 (2.8)	2.5 (2.8)	0.52	(-0.65, 1.69)	0.387
HowRwe	8.9 (2.5)	9.1 (2.4)	-0.35	(-1.15, 0.44)	0.387
Length of stay	18.8 (10.2)	16.6 (12.0)	2.15	(-0.70, 5.01)	0.139
PERFECTER	0.75 (0.11)	0.74 (0.17)	0.059	(-0.10, 0.21)	0.450
**1 Month**					
BADLS	25.0 (12.5)	24.8 (13.6)	-1.50	(-4.56, 1.57)	0.338
EQ-5D SI	0.8 (0.2)	0.9 (0.2)	-0.029	(-0.066, 0.007)	0.113
EQ-5D by Proxy	0.2 (0.3)	0.3 (0.3)	0.028	(-0.042, 0.099)	0.434
EQ-5D Patient	0.6 (0.3)	0.5 (0.4)	0.074	(-0.078, 0.225)	0.341
HowRThey	4.8 (2.6)	4.0 (2.8)	0.601	(-0.040, 1.241)	0.066
MMSE	13.9 (8.0)	13.0 (7.9)	0.29	(-1.04, 1.62)	0.669
**3 Months**					
BADLS	24.6 (13.6)	22.4 (13.4)	-0.46	(-4.35, 3.42)	0.815
EQ-5D SI	0.8 (0.2)	0.9 (0.2)	-0.017	(-0.073, 0.039)	0.556
EQ-5D Proxy	0.3 (0.3)	0.3 (0.3)	0.071	(0.018, 0.124)	0.009
EQ-5D Patient	0.6 (0.3)	0.6 (0.4)	0.024	(-0.052, 0.101)	0.533
HowRThey	4.3 (2.5)	3.4 (2.9)	0.47	(-0.53, 1.47)	0.359
MMSE	13.6 (8.6)	12.5 (8.9)	0.75	(-0.77, 2.27)	0.333
Timed Up & Go	47.3 (33.3)	48.7 (28.1)	-1.54	(-15.38, 12.30)	0.827
**6 Months**					
BADLS	26.4 (14.2)	21.6 (12.0)	1.97	(-1.31, 5.25)	0.239
CDR Score (SI)	1.9 (1.1)	1.7 (1.0)	-0.015	(-0.160, 0.131)	0.845
EQ-5D SI	0.8 (0.2)	0.9 (0.2)	-0.016	(-0.096, 0.063)	0.688
EQ-5D by Proxy	0.4 (0.3)	0.3 (0.4)	0.099	(0.001, 0.198)	0.047
EQ-5D Patient	0.7 (0.3)	0.7 (0.3)	0.057	(-0.104, 0.218)	0.489
HowRThey	4.1 (2.7)	3.3 (2.7)	0.38	(-0.49, 1.25)	0.394
MMSE	13.1 (9.3)	12.2 (8.9)	0.69	(-1.14, 2.53)	0.457

For peer review only

Table 4: Mortality and discharge destination outcomes

Mortality	Intervention (N = 132)(%)	Control (N = 150)(%)	Total (N = 282)(%)
Death in hospital [a]	4 (4.0)	7 (5.7)	11 (4.9)
Death within 30 days of surgery [b]	8 (6.1)	9 (6.1)	17 (6.1)
Death within 6 months of surgery [b]	28 (21.4)	24 (16.2)	52 (18.4)
Total Deaths	30 (22.7)	27 (18.0)	57 (20.2)

NHFD Discharge Destination [c]	Intervention (N = 132)(%)	Control (N = 150)(%)	Total (N = 282)(%)
Died	4 (4.0)	7 (5.7)	11 (4.9)
Nursing Care	19 (19.0)	16 (13.0)	35 (15.7)
Other	3 (3.0)	1 (0.8)	4 (1.8)
Own Home/Sheltered Housing	36 (36.0)	58 (47.2)	94 (42.2)
Rehabilitation Unit (NHS funded care home bed)	0	8 (6.5)	8 (3.6)
Rehabilitation Unit (Hospital bed in another trust)	12 (12.0)	8 (6.5)	20 (9.0)
Residential Care	21 (21.0)	25 (20.3)	46 (20.6)
Unknown	5 (5.0)	0	5 (2.2)
Missing	32 (24.2)	27 (18.0)	59 (20.9)

a: From NHFD data, not available for 59 Scottish participants, 32 intervention and 27 control.
b: 3 patients (1 Intervention, 2 Control) included in 'total deaths' had missing surgery dates. These have not been included in the 'Death within 30 days of surgery' or the 'Death within 6 months of surgery' totals.
C: From NHFD data, not available for 59 Scottish participants, 32 intervention and 27 control.

For peer review only

Mortality

Over the duration of the trial, 57 participants (20%) died. A slightly higher rate was observed in the intervention group than in the control group, (23% versus 18%). Death in hospital was determined from National Hip Fracture Database (NHFD) data and only available for participants in England, thus excluding 59 Scottish participants. Eleven participants (5% of those with NHFD data) died in hospital with more in the control group (6% versus 4%). There were 17 (6%) patients who died within 30 days of surgery and 52 (18.4%) within six months.

Discharge destination

Place of discharge from hospital was identified from the NHFD data, thus unavailable for 59 Scottish participants. The largest proportion of participants returned to their own home or moved into sheltered housing (42%). This destination was more likely in the control group (47%) than the intervention group (36%).

Quality of life

No differences were seen in health-related quality of life (HRQOL) between the control group and intervention group at discharge or one-month follow-up. At three months, a potential beneficial effect of the intervention over control was evidenced for patient HRQOL based upon the EQ-5D-5L by proxy: those in the intervention group had a mean EQ-5D utility score 0.071 higher than control (95% CI: (0.018 to 0.124), p=0.009), a relatively small effect of around one quarter of a standard deviation. A difference of 0.099, in favour of the intervention group, was also seen at the six months follow-up (95% CI: (0.001 to 0.198), p=0.047).

Economic Outcome Feasibility

Intervention costs across the five study wards ranged from £131 to £485 per patient over the study period. There were no significant differences in total costs between groups at any time point except in total health and social care (HSC) costs (including intervention costs) at 3 months using suitsble informant reported data (£4004, 95% CI: £30 to £7979, p=0.049). Total costs (including intervention costs) at each time-point are summarised in **Supplementary Table 4**.

For peer review only - http://bmjopen.bmj.com/site/about/guidelines.xhtml

Where data on HSC were available, the groups did not differ on any outcome measure. Where societal costs data were available, the groups differed in terms of QALYs based on self-rated EQ-5D-5L, the intervention group experiencing a relative gain of 0.121 (95% CI: 0.035 to 0.207, p=0.007) however only 12 intervention cases and 13 control were available for analysis. Suitable informant QALY (self-rated EQ-5D-5L) did not differ between groups.

In terms of HSC costs, where six-month outcome data were available, costs calculated from suitable informant-reported hospital service costs data were higher in intervention than control. A similar pattern was seen in HSC costs if including intervention costs. The groups did not differ on total societal costs, including or excluding intervention costs.

Concordance between HRE and SIR sources on frequency of hospital service use and costs was generally weak, Lin coefficients ranging between between □□= 0.099 and □□= 0.813 for service use across time points. Concordance on hospital costs was high at the baseline (□□ =0.660) but was □□=0.379 at one-month and □□<0.3 at three and six months. Limits of agreement showed that the two measures yielded estimates within £3400 of each other at baseline, £7000 at one-month and similar at six-months, but at three months the limits of agreement were much wider (-£8020, £10693).

Sample Size Calculation

ICCs were estimated, with 95% CIs to inform a sample size calculation. The highest value was estimated for the PERFECT-ER score, 0.748, indicating a substantial degree of between-hospital variation compared to variation between-individuals within hospitals. This is not surprising given the intervention aimed to standardise practice within intervention hospitals

For peer review only

thereby inflating the ICC. At follow-up time points, the ICCs typically ranged between 0.05 and 0.1. At six months, estimates for the MMSE and EQ-5D by proxy were negative and, since a negative value is theoretically not possible and results from estimation error, these were interpreted as being a ‘small’, positive value, near to zero.

DISCUSSION

The findings indicate that modifications are necessary to the trial design for a viable definitive trial. Whilst this study successfully demonstrated the ability to open and recruit from a variety of different UK sites, the recruitment rate was lower than anticipated. There was a lot of missing data for some measures, therefore steps to improve retention of participants at follow-up time-points is warranted, and a sufficiently large inflation of the sample size is required to compensate for missingness. Mortality has been suggested as an appropriate primary outcome. Economic data collection proved burdensome and future studies should reduce the complexity of this task. Consideration of these findings would therefore be warranted when planning a future definitive trial to answer this important research question.

We hypothesise short-term mortality (30-days) may be reduced by the PERFECT-ER intervention due to the cumulative effect of increased good practices across the range of care domains. This builds on previous work (10, 36-38) which recognises complex associations between hospitalisation, pre-admission cognitive impairment, post-admission cognitive impairment, functional decline and mortality. Through this, we would recommend mortality be a proposed primary outcome if a future definitive trial is undertaken.

For peer review only - http://bmjopen.bmj.com/site/about/guidelines.xhtml

Point ICERs for QALY and HSC costs ranged from negative figures (DEMQOL-U, DEMQOL-PROXY, BADLS) resulting from between-group differences favouring the control to very large estimates (EQ-5D-5L and EQ-5D-5L Proxy QALY), far exceeding the £20,000 - £30,000/QALY threshold set by NICE for considering adoption of health technologies (39). Point ICERs for participant QALY and societal costs were all negative, reflecting QALY gains and lower costs in the intervention group. The same pattern was reflected in the SI QALY results. Most ICER had unbounded 95% CIs. Where outcomes were higher in the intervention group and 95% CIs for the ICER could be calculated (EQ-5D-5L QALY), these indicated that we can be confident that the intervention is cost-effective at a willingness to pay well over the NICE threshold (at approximately £122,100) in the case of HSC costs.

For pe

Complex interventions that focus on staff quality improvement and associated implementation methods such as Plan Do Study Act methods (22) present challenges for investigation using RCTs (40). The management and care of people with dementia and CI with hip fracture is complex. This is an example of a 'wicked problem', defined as a complex, messy and stubborn challenge which continually evolve and has, at its core, many reasons for being, with no single solution which can be applied in all circumstances. Ultimately 'wicked problems' are those which cannot be reduced to a set of fixable problems and are often impossible to 'solve' because of incomplete, competing and changing requirements and where the solutions needed are "better or worse" rather than "right or wrong" (41-43). Whilst pragmatic RCTs which offer tailoring and flexibility in experimental interventions are one approach to testing management strategies for such healthcare challenges, other research methodologies such as case study may also provide important insights. Further careful consideration of which methodological approach may be most

For peer review only - http://bmjopen.bmj.com/site/about/guidelines.xhtml

appropriate to answer this research question may therefore be warranted before automatically embarking on a clinical trial pathway.

CONCLUSION

This study has demonstrated that PERFECT-ER can be implemented and widely accepted across a number of different health services in the UK's NHS. We have shown it is feasible, with modifications, to undertake a definitive trial and economic evaluation using the developed and refined recruitment and consenting practices. However, care of people with CI and hip fracture poses a 'wicked problem' and further definitive research using a RCT approach should be deliberated against other methods of evaluation.

DECLARATIONS

Ethical Approval: Ethical approval for the trial was given by Camden and Kings Research Ethics Committee (reference number: 16/LO/0621) and Scotland Research Ethics Committee A (reference number: 16/SS/0086).

Trial registration number: ISRCTN 99336264.

Funding: This work was supported by NIHR Programme Grants for Applied Research (PGfAR) Programme grant number [ref: DTC-RP-PG-0311-12004].

For peer review only - http://bmjopen.bmj.com/site/about/guidelines.xhtml

FIGURE AND TABLE LEGENDS

Figure 1: Patient Flow Diagram

Table 1: Recruitment rates by Centre

Table 2: Participant and suitable informant baseline characteristics

Table 3: Estimates of outcome

Table 4: Mortality and discharge destination outcomes

Supplementary Table 1: Data collection schedule

Supplementary Table 2: Hospital baseline characteristics

Supplementary Table 3: Available data for analysis

Supplementary Table 4: Mean costs (standard errors): Health & social care services for participant, unpaid carer (SI) costs, out-of-pocket costs, total health & social care and societal costs over prior three months, at baseline and one-, three-, and six-month follow-ups (£, 2016-17).

For peer review only

For peer review only - http://bmjopen.bmj.com/site/about/guidelines.xhtml

Contributorship statement

JLC, SPH, LS, FP, CH, TB, BP, SD, MK, AM, MP, OS, JW, RH, CB, and CF made substantial contributions to the conception or design of the work;

· SPH, TB led on the acquisition of the data

· SPH, LS, CF and JC led the statistical analysis and interpretation of data for the work;

· JC led the drafting of the paper. All authors were involved in revising it critically for important intellectual content;

· All authors reviewed the paper and gave their final approval of the version to be published;

· All authors give their agreement to be accountable for all aspects of the work in ensuring that questions related to the accuracy or integrity of any part of the work are appropriately investigated and resolved.

Data sharing statement

The trial was completed prior to 1st of January 2019.

References

1. Ravindrarajah R, Hazra NC, Charlton J, Jackson SHD, Dregan A, Gulliford MC. Incidence and mortality of fractures by frailty level over 80 years of age: cohort study using UK electronic health records. BMJ Open. 2018;8(1):e018836.
2. Griffin XL, Parsons N, Achten J, Fernandez M, Costa ML. Recovery of health-related quality of life in a United Kingdom hip fracture population. The Warwick Hip Trauma Evaluation--a prospective cohort study. Bone Joint J. 2015;97-B(3):372-82.
3. Leal J, Gray AM, Prieto-Alhambra D, Arden NK, Cooper C, Javaid MK, et al. Impact of hip fracture on hospital care costs: a population-based study. Osteoporos Int. 2016;27(2):549-58.
4. Sampson EL, Leurent B, Blanchard MR, Jones L, King M. Survival of people with dementia after unplanned acute hospital admission: a prospective cohort study. Int J Geriatr Psychiatry. 2013;28(10):1015-22.
5. Boaden A. Fix Dementia Care Hospitals: Alzheimer's Society 2016 [Available from: https://www.alzheimers.org.uk/sites/default/files/migrate/downloads/fix_dementia_care_-_hospitals.pdf.
6. Fogg C, Meredith P, Bridges J, Gould GP, Griffiths P. The relationship between cognitive impairment, mortality and discharge characteristics in a large cohort of older adults with unscheduled admissions to an acute hospital: a retrospective observational study. Age Ageing. 2017;46(5):794-801.

For peer review only - http://bmjopen.bmj.com/site/about/guidelines.xhtml

7. Seitz DP, Adunuri N, Gill SS, Rochon PA. Prevalence of dementia and cognitive impairment among older adults with hip fractures. J Am Med Dir Assoc. 2011;12(8):556-64.
8. Myers JS, Grigsby J, Teel CS, Kramer AM. Nurses' assessment of rehabilitation potential and prediction of functional status at discharge from inpatient rehabilitation. Int J Rehabil Res. 2009;32(3):264-6.
9. Featherstone K, Northcott A, Bridges J, Harrison Denning K, Harden J, Bale S, et al. Summary Results: An evidence based investigation examining the care people living with dementia receive following an acute hospital admission.: London: Stories of Dementia; 2018 [Available from: http://www.storiesofdementia.com/2018/04/research-report.html.
10. Royal College of Psychiatrists, National Audit of Dementia (Care in General Hospitals) Spotlight Audit: Assessment of delirium in hospital for people with dementia 2018 [Available from: https://www.hqip.org.uk/wp-content/uploads/2018/08/NAD-Delirium-Spotlight-report-Aug-2018.pdf.
11. Gupta A, Havelock W. A new future for hip fracture care--orthogeriatrician lead in an 'Acute' Hip Unit. Clin Med (Lond). 2014;14(6):591-6.
12. Riemen AH, Hutchison JD. The multidisciplinary management of hip fractures in older patients. Orthop Trauma. 2016;30(2):117-22.
13. National Collaborating Centre for Mental Health (UK), Dementia: A NICE-SCIE Guideline on Supporting People With Dementia and Their Carers in Health and Social Care. British Psychological Society. 2007 [Available from: https://www.scie.org.uk/publications/misc/dementia/dementia-fullguideline.pdf?res=true.
14. Gill N, Hammond S, Cross J, Smith T, Lambert N, Fox C. Optimising care for patients with cognitive impairment and dementia following hip fracture. Z Gerontol Geriatr. 2017;50(Suppl 2):39-43.
15. Schulz KF, Altman DG, Moher D. CONSORT 2010 statement: updated guidelines for reporting parallel group randomized trials. Ann Intern Med. 2010;152(11):726-32.
16. Hammond SP, Cross JL, Shepstone L, Backhouse T, Henderson C, Poland F, et al. PERFECTED enhanced recovery (PERFECT-ER) care versus standard acute care for patients admitted to acute settings with hip fracture identified as experiencing confusion: study protocol for a feasibility cluster randomized controlled trial. Trials. 2017;18(1):583-.
17. Moore GF, Audrey S, Barker M, Bond L, Bonell C, Hardeman W, et al. Process evaluation of complex interventions: Medical Research Council guidance. BMJ. 2015;350:h1258.
18. Harwood RH, Goldberg SE, Whittamore KH, Russell C, Gladman JR, Jones RG, et al. Evaluation of a Medical and Mental Health Unit compared with standard care for older people whose emergency admission to an acute general hospital is complicated by concurrent 'confusion': a controlled clinical trial. Acronym: TEAM: Trial of an Elderly Acute care Medical and mental health unit. Trials. 2011;12:123.
19. Young J, Cheater F, Collinson M, Fletcher M, Forster A, Godfrey M, et al. Prevention of delirium (POD) for older people in hospital: study protocol for a randomised controlled feasibility trial. Trials. 2015;16:340.
20. Mental Capacity Act 2005. London: HMSO; 2005.
21. Adults with Incapacity (Scotland) Act 2000. In: Parliment S, editor.: HMSO; 2000.
22. NHS Improvement. *Plan, do, study, act (PDSA) cycles and the model for improvement* 2018 [Available from: https://improvement.nhs.uk/documents/2142/plan-do-study-act.pdf
23. https://www.nhfd.co.uk/ [Internet]. [cited 30 6 2021].
24. Beecham JK, Knapp MRJ. Costing psychiatric interventions. In: Thornicroft G, Brewin C, Wing JK, editors. Measuring Mental Health Needs. 2nd ed. London: Gaskell; 2001. p. 220-4.

For peer review only - http://bmjopen.bmj.com/site/about/guidelines.xhtml

25. Curtis L, Burns A. Unit Costs of Health and Social Care 2017. Canterbury: Personal Social Services Research Unit, University of Kent; 2017.
26. NHS Improvement NHS trusts and NHS foundation trusts. [Internet]. 2017. Available from: https://improvement.nhs.uk/resources/reference-costs/.
27. https://www.england.nhs.uk/pay-syst/national-tariff/NHS Improvement and NHS England. National Tariff: currencies and prices. [Internet]. 2017/18 [cited 1 7 2021]. Available from: https://improvement.nhs.uk/documents/1044/2017-18_and_2018-19_National_Tariff_Payment_System.pdf.
28. NHS Digital. Prescription cost analysis England 2017 [Internet]. 2018. Available from: https://digital.nhs.uk/data-and-information/publications/statistical/prescription-cost-analysis/prescription-cost-analysis-england-2017.
29. Ukoumunne OC. A comparison of confidence interval methods for the intraclass correlation coefficient in cluster randomized trials. Statistics in Medicine. 2002;21(24):3757-74.
30. Howard R, Phillips P, Johnson T, O'Brien J, Sheehan B, Lindesay J, et al. Determining the minimum clinically important differences for outcomes in the DOMINO trial. International Journal of Geriatric Psychiatry. 2011;26(8):812-7.
31. Rowen D, Mulhern B, Banerjee S, Hout B, Young TA, Knapp M, et al. Estimating preference-based single index measures for dementia using DEMQOL and DEMQOL-Proxy. Value Health. 2012;15(2):346-56.
32. van Hout B, Janssen MF, Feng YS, Kohlmann T, Busschbach J, Golicki D, et al. Interim Scoring for the EQ-5D-5L: Mapping the EQ-5D-5L to EQ-5D-3L Value Sets. Value in Health. 2012;15(5):708-15.
33. Lin LI. A concordance correlation coefficient to evaluate reproducibility. Biometrics. 1989;45(1):255-68.
34. Bland JM, Altman DG. Statistical Methods for Assessing Agreement between Two Methods of Clinical Measurement. Lancet. 1986;1(8476):307-10.
35. Steichen TJ, Cox NJ. sg84: Concordance correlation coefficient. Stata Technical Bulletin Reprints. 1998;8:137-43.
36. Dementia Action Alliance 2018 [Available from: https://www.dementiaaction.org.uk/dementiafriendlyhospitalscharter.
37. Health Foundation Spotlight on Dementia Care 2011 [Available from: https://www.health.org.uk/sites/default/files/SpotlightOnDementiaCare_fullversion.pdf.
38. Department of Health Living well with dementia: A National Dementia Strategy 2009 [Available from: https://assets.publishing.service.gov.uk/government/uploads/system/uploads/attachment_data/file/168220/dh_094051.pdf.
39. NICE Guide to the methods of technology appraisal 2013 [Available from: https://www.nice.org.uk/process/pmg9/resources/guide-to-the-methods-of-technology-appraisal-2013-pdf-2007975843781.
40. Paparini S, Green J, Papoutsi C, Murdoch J, Petticrew M, Greenhalgh T, et al. Case study research for better evaluations of complex interventions: rationale and challenges. BMC Medicine. 2020;18(1):301.
41. Buchanan R. Wicked Problems in Design Thinking. Design Issues. 1992;8(2):5-21.
42. Horst WJR, Webber MM. Dilemmas in a General Theory of Planning. Policy Sciences. 1973;4(2):155-69.
43. Krippendorff K. The Semantic Turn: A New Foundation for Design (1st ed.). . Boca Raton: CRC Press; 2005. 368 p.

For peer review only - http://bmjopen.bmj.com/site/about/guidelines.xhtml

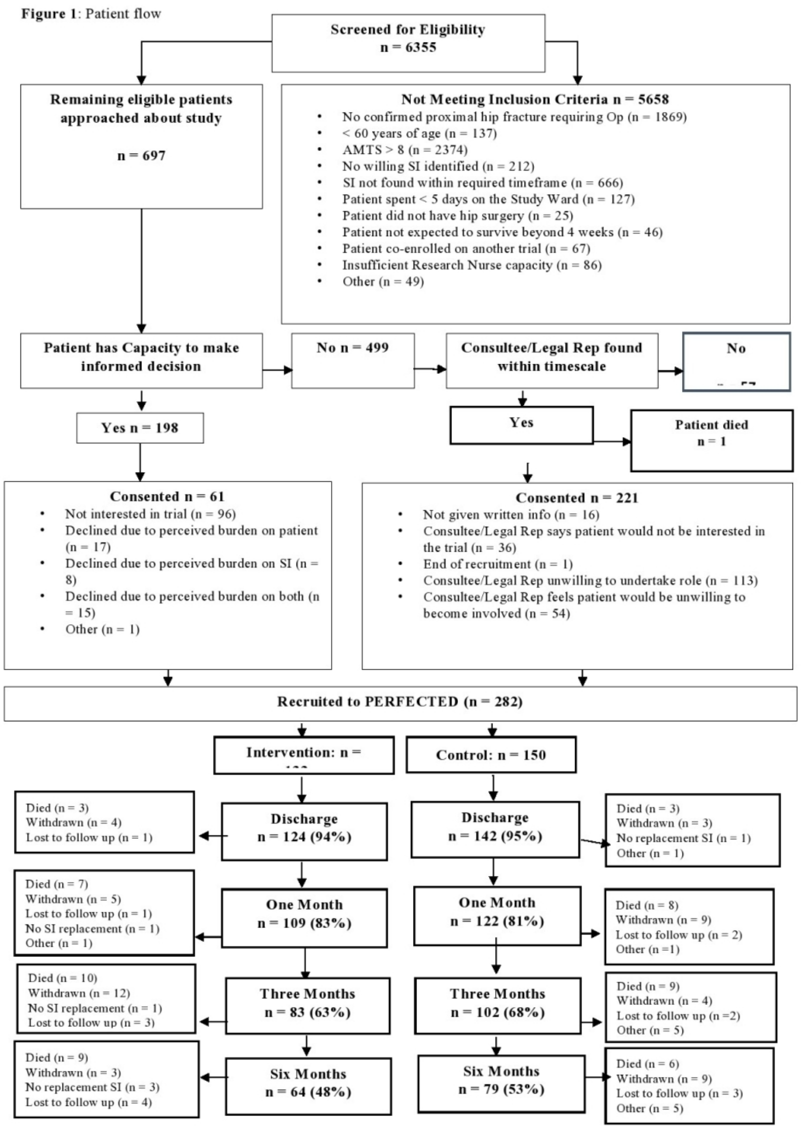

Figure 1 Patient Flow Diagram Statement of authorship: Figure created by the authors

104x148mm (300 x 300 DPI)

Supplementary Table 1: Data collection schedule:

Statement of authorship: Table created by the authors

	**Admission**	**Enrolment**	**Baseline**	**Post-operative period**			
**TIMEPOINT**	**-T2**	**-T1**	**0**	**D**	**T1[a]**	**T2**	**T3**
PRE-INTERVENTION:							
Eligibility screen	■						
Study information provided	■						
Informed consent given		■					
ASSESSMENTS:							
MMSE-2: SV (Patient)			■		■	■	■
DEMQOL (Patient)			■		■	■	■
EQ-5D-5L self-complete (Patient)			■		■	■	■
howRwe (Patient)			■	■ b	■ b		
CDR (Patient)			■				■
Patient care profile (Patient)				■ b	■ b		
Timed Up & Go (Patient)						■	
BADLS (Suitable Informant)			■		■	■	■
DEMQOL-Proxy (Suitable Informant)			■		■	■	■
EQ-5D-5L Proxy (Suitable Informant)			■		■	■	■
EQ-5D-5L Carer self-report (Suitable Informant)			■		■	■	■
CSRI[c] (Suitable Informant)			■		■	■	■
Number of days in institutional care (Suitable Informant)					■	■	■
howRthey (Suitable Informant)			■	■ b	■ b	■	■
Patient's place of residence (Suitable Informant)			■ d		■	■	■
CDR (Suitable Informant)			■				■
IQCODE (Suitable Informant)			■				
Length of stay in index hospitalisation				■ e	■ e	■	■
Discharge destination from index hospitalisation					■	■	■
Mortality					■	■	■
Hospital re-admission rates							■
Hospital service use[f]			■		■	■	■
4AT			■	■ b	■ b		
Charlson Co-morbidity Index (CCI)			■				
NHFD (England only)							■ g

[a] PERFECT-ER and treatment as usual continue up until discharge from study ward. Due to differences in length of stay in the study sites, T1 assessments may take place in the study site for some participants;
[b] Patients may be discharged from study ward before or after T1. Measure to be collected at whenever this point maybe ± five days;
[c] duration of retrospective period covered varies by assessment point;
[d] pre-baseline ordinary residence;
[e] If patient is still in acute hospital at thirty days this will be recorded;
[f] from hospital patient records, of service use within site of index hospitalisation
[g] extracted from NHFD post recruitment window closing

Supplementary Table 2: Hospital baseline characteristics

Statement of authorship: Table created by the authors

	**Intervention**			**Control** [a,b]		
	**Median**	**Max**	**Min**	**Median**	**Max**	**Min**
Number of Beds on Ward	27.0	41.0	15.0	28.0	38.0	25.0
Number of Bed Days on Ward in last 12 months	9855.0	14965.0	5475.0	10220.0	13870.0	9038.0
Occupied Bed Rate (%) in last 12 months	93.0	99.0	90.0	96.0	100	93.0
Number of Falls on Ward in last 12 months	42.0	82.0	25.0	60.0	111.0	32.0
Number of Deaths on Ward in last 12 months	30.0	66.0	7.0	34.0	68.0	13.0
Registered/Qualified Nurses	22.0	27.5	16.2	19.8	26.8	12.0
Geriatricians	1.0	2.6	0.5	1.0	1.0	0.8
Orthopaedic Surgeons	0.3	1.0	0.0	1.5	12.0	0.0
Other Consultants	0.0	0.4	0.0	0.0	4.7	0.0
Other Registrars	0.5	1.0	0.0	1.0	5.6	0.4
Other Junior Doctors	1.5	2.5	0.0	3.0	3.0	1.0

a One hospital (Control) missing all data
b One hospital (Control) missing data for *Number of Falls on the Ward* in last 12 months.

For peer review only

Supplementary Table 3: Available data for analysis

Statement of authorship: Table created by the authors

Time point & outcome measure	Intervention (N = 132)	Control (N = 150)
**Baseline**		
HowRThey	5 (3.8)	13 (8.7)
HowRwe	39 (29.5)	56 (37.3)
EQ-5D – Patient	40 (30.3)	63 (42.0)
EQ-5D – SI	7 (5.3)	11 (7.3)
EQ-5D – Proxy	6 (4.5)	14 (9.3)
MMSE	4 (3.0)	13 (8.7)
BADLS	5 (3.8)	9 (6.0)
4AT	5 (3.8)	18 (12.0)
CDR	5 (3.8)	13 (8.7)
**Discharge**	**Expected = 123**	**Expected = 143**
HowRthey	116 (94.3)	116 (81.1)
HowRwe	84 (68.3)	72 (50.3)
4AT	116 (94.3)	103 (72.0)
Length of Stay	121 (98.4)	142 (99.3)
PERFECTER Score	122 (99.2)	141 (98.6)
**1 Month**	**Expected = 108**	**Expected = 122**
MMSE	106 (98.1)	111 (91.0)
BADLS	104 (96.3)	112 (91.8)
EQ-5D Patient	84 (77.8)	78 (63.9)
EQ-5D SI	106 (98.1)	110 (90.2)
EQ-5D Proxy	105 (97.2)	112 (91.8)
HowRthey	102 (94.4)	110 (90.2)
**3 Months**	**Expected = 83**	**Expected = 102**
MMSE	81 (97.6)	97 (95.1)
Timed Up & Go	44 (53.0)	50 (49.0)
BADLS	81 (97.6)	96 (94.1)
HowRthey	82 (98.8)	94 (92.2)
EQ-5D Patient	61 (73.5)	69 (67.6)
EQ-5D SI	81 (97.6)	97 (95.1)
EQ-5D Proxy	82 (98.8)	98 (96.1)
**6 Months**	**Expected = 64**	**Expected = 80**
MMSE	63 (98.4)	72 (90.0)
BADLS	61 (95.3)	77 (96.3)
HowRthey	64 (100)	76 (95.0)
EQ-5D Patient	36 (56.3)	43 (53.8)
EQ-5D SI	48 (75.0)	65 (81.3)
EQ-5D Proxy	44 (68.8)	65 (81.3)
Global CDR	64 (100)	66 (82.5)

a: Estimated as negative

For peer review only

Supplementary Table 4: Mean costs (standard errors): Health & social care services for participant, unpaid carer (SI) costs, out-of-pocket costs, total health & social care and societal costs over prior three months, at baseline and one-, three-, and six-month follow-ups (£, 2016-17)

Statement of authorship: Table created by the authors

Cost	Intervention (n=132)			Control (n=150)			Intervention-control	
	**n**	**Mean**	**SE**	**n**	**Mean**	**SE**	**Mean difference**	**95% CI**
**Baseline**								
Health & social care (HRE)	125	3740	709	135	3196	691	544	-1697, 2784
Health & social care (SIR)	123	3458	653	130	3148	642	310	-1761, 2381
Health & social care (SIR+)	125	3544	663	135	3094	645	450	-1642, 2543
Societal (HRE)[f]	95	9661	949	100	9783	932	-122	-3131, 2886
Societal (SIR)[f]	93	9249	946	97	9823	934	-574	-3581, 2433
Societal (SIR+)[f]	95	9299	886	100	9635	867	-336	-3140, 2469
**1 month**								
Intervn.+Health & social care (HRE)	89	12859	531	99	11636	509	1223	-441, 2886
Intervn.+Health & social care (SIR)	89	13890	980	95	11489	974	2401	-726, 5527
Intervn.+Health & social care (SIR+)	89	13894	945	99	11574	922	2320	-667, 5306
Intervn.+Societal (HRE)[f]	75	14191	526	80	13988	511	203	-1456, 1862
Intervn.+Societal (SIR)[f]	75	15032	1023	76	14123	1023	908	-2364, 4180
Intervn.+Societal (SIR+)[f]	75	15036	1023	80	14141	1000	895	-2341, 4131
**3 months**								
Intervn.+Health & social care (HRE)	75	9193	1721	88	5946	1684	3247	-2200, 8695
Intervn.+Health & social care (SIR)	75	8315	1258	87	4310	1226	4004*	30, 7979
Intervn.+Health & social care (SIR+)	75	8325	1274	88	4621	1236	3704	-311, 7719
Intervn.+Societal (HRE)[f]	64	12794	1909	71	10748	1846	2047	-3961, 8054
Intervn.+Societal (SIR)[f]	64	11983	1341	70	8923	1297	3060	-1161, 7281
Intervn.+Societal (SIR+)[f]	64	11995	1293	71	9243	1243	2752	-1305, 6808
**6 months**								
Intervn.+Health & social care (HRE)	57	6807	1402	64	5146	1413	1661	-2842, 6164
Intervn.+Health & social care (SIR)	57	6827	999	64	4308	965	2519	-624, 5661
Intervn.+Health & social care (SIR+)	57	6839	1004	64	4308	971	2531	-629, 5692
Intervn.+Societal (HRE)[f]	52	11511	1462	54	12478	1476	-967	-5666, 3733
Intervn.+Societal (SIR)[f]	52	11514	1506	54	11483	1536	31	-4836, 4897
Intervn.+Societal (SIR+)[f]	52	11528	1511	54	11483	1541	44	-4839, 4928

Note: NHS CC=NHS continuing care; HRE=health records extraction; SIR=Suitable Informant-reported; SIR+= corresponding hospital costs data from HRE used when costs were missing from the SIR dataset; Intervn.=Intervention costs

a Funded by NHS or Social Services

b Provided by NHS or Social Services

c expenditure by self or family on equipment purchases

d expenditure by self or family on travel to appointments

e unpaid carers' time in care and support to participant

f societal costs include: participant's health and social care costs; unpaid carers' time in care and support to participant; expenditure by self or family on travel to appointments, equipment purchases

For peer review only

CONSORT 2010 checklist of information to include when reporting a pilot or feasibility trial*

Section/Topic	Item No	Checklist item	Reported on page No
**Title and abstract**			
	1a	Identification as a pilot or feasibility randomised trial in the title	1
	1b	Structured summary of pilot trial design, methods, results, and conclusions (for specific guidance see CONSORT abstract extension for pilot trials)	3-4
**Introduction**			
Background and objectives	2a	Scientific background and explanation of rationale for future definitive trial, and reasons for randomised pilot trial	4-5
	2b	Specific objectives or research questions for pilot trial	6
**Methods**			
Trial design	3a	Description of pilot trial design (such as parallel, factorial) including allocation ratio	7
	3b	Important changes to methods after pilot trial commencement (such as eligibility criteria), with reasons	NA
Participants	4a	Eligibility criteria for participants	7-8
	4b	Settings and locations where the data were collected	7
	4c	How participants were identified and consented	8-9
Interventions	5	The interventions for each group with sufficient details to allow replication, including how and when they were actually administered	9-10
Outcomes	6a	Completely defined prespecified assessments or measurements to address each pilot trial objective specified in 2b, including how and when they were assessed	10-11
	6b	Any changes to pilot trial assessments or measurements after the pilot trial commenced, with reasons	NA
	6c	If applicable, prespecified criteria used to judge whether, or how, to proceed with future definitive trial	NA
Sample size	7a	Rationale for numbers in the pilot trial	8
	7b	When applicable, explanation of any interim analyses and stopping guidelines	NA
Randomisation:			
Sequence generation	8a	Method used to generate the random allocation sequence	7
	8b	Type of randomisation(s); details of any restriction (such as blocking and block size)	7
Allocation concealment mechanism	9	Mechanism used to implement the random allocation sequence (such as sequentially numbered containers), describing any steps taken to conceal the sequence until interventions were assigned	7

For peer review only

Implementation	10	Who generated the random allocation sequence, who enrolled participants, and who assigned participants to interventions	6-7
Blinding	11a	If done, who was blinded after assignment to interventions (for example, participants, care providers, those assessing outcomes) and how	NA
	11b	If relevant, description of the similarity of interventions	
Statistical methods	12	Methods used to address each pilot trial objective whether qualitative or quantitative	11-12
**Results**			
Participant flow (a diagram is strongly recommended)	13a	For each group, the numbers of participants who were approached and/or assessed for eligibility, randomly assigned, received intended treatment, and were assessed for each objective	13-14,
	13b	For each group, losses and exclusions after randomisation, together with reasons	13
Recruitment	14a	Dates defining the periods of recruitment and follow-up	7, 14, 17,18
	14b	Why the pilot trial ended or was stopped	
Baseline data	15	A table showing baseline demographic and clinical characteristics for each group	14/15
Numbers analysed	16	For each objective, number of participants (denominator) included in each analysis. If relevant, these numbers should be by randomised group	17/18
Outcomes and estimation	17	For each objective, results including expressions of uncertainty (such as 95% confidence interval) for any estimates. If relevant, these results should be by randomised group	17/18
Ancillary analyses	18	Results of any other analyses performed that could be used to inform the future definitive trial	17-20
Harms	19	All important harms or unintended effects in each group (for specific guidance see CONSORT for harms)	NA
	19a	If relevant, other important unintended consequences	NA
**Discussion**			
Limitations	20	Pilot trial limitations, addressing sources of potential bias and remaining uncertainty about feasibility	21
Generalisability	21	Generalisability (applicability) of pilot trial methods and findings to future definitive trial and other studies	21
Interpretation	22	Interpretation consistent with pilot trial objectives and findings, balancing potential benefits and harms, and considering other relevant evidence	23
	22a	Implications for progression from pilot to future definitive trial, including any proposed amendments	23
**Other information**			
Registration	23	Registration number for pilot trial and name of trial registry	23
Protocol	24	Where the pilot trial protocol can be accessed, if available	**Trial registration:** ISR CTN, 99336264 . Registered on 5 September 2016.

For peer review only

			PERFECTED enhanced recovery (PERFECT-ER) care versus standard acute care for patients admitted to acute settings with hip fracture identified as experiencing confusion: study protocol for a feasibility cluster randomized controlled trial | Trials | Full Text (biomedcentral.com)
Funding	25	Sources of funding and other support (such as supply of drugs), role of funders	24
	26	Ethical approval or approval by research review committee, confirmed with reference number	23

Citation: Eldridge SM, Chan CL, Campbell MJ, Bond CM, Hopewell S, Thabane L, et al. CONSORT 2010 statement: extension to randomised pilot and feasibility trials. BMJ. 2016;355.
*We strongly recommend reading this statement in conjunction with the CONSORT 2010, extension to randomised pilot and feasibility trials, Explanation and Elaboration for important clarifications on all the items. If relevant, we also recommend reading CONSORT extensions for cluster randomised trials, non-inferiority and equivalence trials, non-pharmacological treatments, herbal interventions, and pragmatic trials. Additional extensions are forthcoming: for those and for up to date references relevant to this checklist, see www.consort-statement.org.

For peer review only

Short title: Care of patients experiencing hip fracture & confusion: PERFECTED CRCT

Long title: Enhancing recovery of patients admitted to acute settings with hip fracture who are identified as experiencing confusion: a multi-centre, cluster-randomised controlled, feasibility trial of the PERFECTED Enhanced Recovery (PERFECT-ER) care versus standard acute care

Version	2.0
Date	17.06.2016
Sponsor	University of East Anglia (UEA)
Trial registration	ISRCTN
REC Reference	16/LO/0621 (England)<br>16/55/0086 (Scotland)

Authorisation: Chief Investigator

Name	George Christopher Fox
Role	Professor of Clinical Psychiatry
Signature	
Date	

Authorisation: NCTU Director

Name	Ann Marie Swart
Role	Professor of Medicine and Epidemiology
Signature	
Date	

For peer review only - http://bmjopen.bmj.com/site/about/guidelines.xhtml

Authorisation: Sponsor Representative

Name	Yvonne Kirkham
Role	Project Officer
Signature	
Date	

Authorisation: Senior Operations Staff

Name	Erika Sims
Role	Senior Clinical Trial Operations Manager
Signature	
Date	

Authorisation: Statistician

Name	Lee Shepstone
Role	Professor of Medical Statistics
Signature	
Date	

For peer review only

For peer review only - http://bmjopen.bmj.com/site/about/guidelines.xhtml

Table of Contents

1. Administrative information........1
1.1 Compliance........1
1.2 Sponsor........2
1.3 Structured trial summary........3
1.4 Roles and responsibilities........6
1.4.1 Protocol contributors........6
1.4.2 Role of trial sponsor and funders........6
1.4.3 Trial Team........6
1.4.4 Trial Management Group........7
1.4.5 Programme Steering Committee........7
1.4.6 Data Monitoring and Ethics Committee (DMEC)........7
1.4.7 Other Trial Oversight Groups (Programme Advisory Group)........8
2 Trial Diagram........9
3 Abbreviations........10
4 Introduction........10
4.1 Background and Rationale........10
4.1.2 Enhanced Recovery Pathway (ERP)........11
4.1.3 The PERFECTED Research programme........11
4.3 Objectives........11
4.4 Trial Design........12
5 Methods........14
5.1 Site Selection........14
5.1.1 Study Setting........14
5.1.2.1 Principal Investigator's (PI) Qualifications and Agreements........15
5.1.2.2 Resourcing at site........15
5.2 Site approval and activation........16
5.3 Participants........16
5.3.1 Eligibility Criteria........16
5.3.2 Participant selection........16
5.3.2.1 Patient participant inclusion and exclusion criteria (trial)........17
5.3.2.2 Suitable informant participant inclusion and exclusion criteria (trial)........17
5.3.3.1 Recruiting patients assessed not to have capacity (England)........19

For peer review only - http://bmjopen.bmj.com/site/about/guidelines.xhtml

5.3.3.2 Involving patients who [illegible] may lose capacity (England) 20
5.3.3.3 Recruiting patients assessed not to have capacity (Scotland) 21
5.3.3.4 Recruiting patients who may lose capacity (Scotland) 22
5.3.4 Patient and suitable informant recruitment diagram 23
5.3.5 Withdrawal of patient participant and/or suitable informant 24
5.3.6 Early stopping of follow-up 25
5.3.7 Patient dies during trial 25
5.3.8 Loss to Follow-up 26
5.3.9 Participant moves 26
5.3.9.1 Co-enrolment Guidance 26
5.3.9.2 Screening procedures and pre-consent 26
5.5 Interventions 27
5.5.1 Active Arm 27
5.5.1.1 TIDieR Framework 29
5.5.2 Control arm 30
5.5.3 Adherence monitoring 30
5.6 Outcomes 30
5.7 Assessment Schedule 33
5.8 Sample Size 34
5.9 Recruitment and Retention 35
5.9.1 Recruitment 35
5.9.2 Retention 35
5.10 Assignment of Intervention 36
5.10.1 Allocation 36
5.10.2 Blinding 36
5.11 Data Collection, Management and Analysis 36
5.11.1 Data Collection Methods 36
5.11.2 Data management 37
5.11.2.1 Physical storage of identifiable and anonymised information 38
5.11.3 Non-Adherence and Non-Retention 38
5.11.4 Statistical Methods 39
5.11.4.1 Statistical Analysis Plan 39
5.11.5 Economic evaluations 39
5.11.6 Additional Analyses – Subgroup 41

For peer review only

For peer review only - http://bmjopen.bmj.com/site/about/guidelines.xhtml

5.11.7 Additional Analyses – Adjusted ... 41

5.11.8 Analysis Population and Missing Data ... 41

5.12 Data Monitoring ... 41

5.12.1 Data Monitoring and Ethics Committee (DMEC) ... 41

5.12.2 Interim analyses ... 41

5.12.3 Data monitoring for harm ... 41

6 Safety reporting procedures ... 43

6.1 PERFECTED Operationalised Definitions ... 43

6.1.1 Adverse events (non-reportable) ... 43

6.1.2 Expected serious adverse events – standard reporting ... 43

7 Quality assurance and control ... 44

7.1 Risk Assessment ... 44

7.2 Central Monitoring at NCTU ... 44

7.3 On-site Monitoring ... 44

7.3.1 Direct access to participant records ... 44

7.4 Trial oversight ... 44

7.4.1 Trial Management Team ... 45

7.4.2 Trial Management Group ... 45

7.4.3 Programme Steering Committee ... 45

7.4.4 Independent Data Monitoring Committee ... 45

7.4.5 Trial Sponsor ... 46

8. Ethics and Dissemination ... 47

8.1 Research Ethics Approval ... 47

8.2 Competent Authority Approvals ... 47

8.3 Other Approvals ... 47

8.4. Protocol Amendments ... 48

8.5 Declaration of Interests ... 48

8.6. Confidentiality ... 48

8.7 Indemnity ... 48

8.8 Finance ... 49

8.9 Archiving ... 49

8.10 Access to Data ... 49

8.11 Publication policy ... 50

8.11.1 Trial results ... 50

For peer review only

For peer review only - http://bmjopen.bmj.com/site/about/guidelines.xhtml

8.11.2 Authorship ... 50
8.11.3 Reproducible research ... 50
9. Ancillary Studies ... 51
9.1 Patient and suitable informant; recruitment, data collection, consent and withdrawal for qualitative study ... 51
9.1.1. Inclusion and exclusion criteria for patient participants in qualitative study ... 52
9.1.2 Inclusion and exclusion criteria for suitable informants in qualitative study ... 53
9.2 Supporting Public Patient Involvement (PPI) members in research activities ... 53
9.2.1Potential issues associated with PPI members undertaking research activities ... 54
9.3. Patient and Suitable Informant recruitment diagram ... 56
9.4 NHS professionals: recruitment, data collection, consent and withdrawal ... 56
9.4.1 Inclusion and exclusion criteria for NHS professionals in qualitative study ... 57
9.5 Qualitative procedure schedule ... 58
9. Qualitative data storage and transcription ... 58
10. Protocol Amendments ... 59
11. References ... 60

For peer review only

For peer review only - http://bmjopen.bmj.com/site/about/guidelines.xhtml

1. Administrative information

This document was constructed using the Norwich Clinical Trials Unit (NCTU) Protocol template Version 2.0. It describes the PERFECTED trial sponsored by the University of East Anglia (UEA) and co-ordinated by NCTU.

It provides information about procedures for participating wards entering patients into the trial, and provides sufficient detail to enable: an understanding of the background, rationale, objectives, trial population, intervention, methods, statistical analyses, ethical considerations, dissemination plans and administration of the trial; replication of key aspects of trial methods and conduct; and appraisal of the trial's scientific and ethical rigour from the time of ethics approval through to dissemination of the results. The protocol should not be used as an aide-memoire or guide for the treatment of other patients. Every care has been taken in drafting this protocol, but corrections or amendments may be necessary. These will be circulated to registered investigators in the trial. Sites entering participants for the first time should confirm they have the correct version through a member of the trial team at NCTU.

NCTU supports the commitment that its trials adhere to the SPIRIT guidelines. The protocol template is based on the Standard Protocol Items: Recommendations for Interventional Trials (SPIRIT) 2012 Statement for protocols of clinical trials (Chan et al, 2013a). The SPIRIT Statement Explanation and Elaboration document (Chan et al 2013b) can be referred to, or a member of NCTU Protocol Review Committee can be contacted for further details about specific items.

1.1 Compliance

The trial will be conducted in compliance with the approved protocol, the Declaration of Helsinki (2008), the principles of Good Clinical Practice (GCP), the UK Data Protection Act, and the National Health Service (NHS) Research Governance Framework for Health and Social Care (RGF). Agreements that include detailed roles and responsibilities will be in place between participating sites and NCTU.

Participating sites will inform NCTU as soon as they are aware of a possible serious breach of compliance, so that NCTU can fulfil its requirement to report the breach if necessary to the trial Sponsor. For the purposes of this regulation a 'serious breach' is one that is likely to affect to a significant degree:

For peer review only - http://bmjopen.bmj.com/site/about/guidelines.xhtml

- The safety or physical or mental integrity of the subjects in the trial, or
- The scientific value of the trial.

1.2 Sponsor

UEA is the trial sponsor and has delegated responsibility for the overall management of the trial to Professor Fox (Chief Investigator) and NCTU. Queries relating to sponsorship of this trial should be addressed to the NCTU Director or via the trial team.

For peer review only

For peer review only - http://bmjopen.bmj.com/site/about/guidelines.xhtml

1.3 Structured trial summary

Primary Registry and Trial Identifying Number	Name of primary registry, and the unique ID number assigned by the primary registry to this trial.
Date of Registration in Primary Registry	Date when trial was officially registered in the primary registry.
Secondary Identifying Numbers	Universal Trial Number (UTN) 1111-1180-9350<br>Identifiers assigned by the sponsor: R19858<br>Funder reference: DTC-RP-PG-0311-12004
Source of Monetary or Material Support	National Institute for Health Research (NIHR) Programme Grants for Applied Research (PGfAR) is providing funding for research costs for the project duration to cover trial set up, trial conduct, analysis and report writing.
Sponsor	University of East Anglia
Contact for Public Queries	perfected@uea.ac.uk
Contact for Scientific Queries	Chief Investigator, Prof Chris Fox, Norwich Medical School, University of East Anglia, Norwich Research Park, Norwich, Norfolk, NR4 7TJ. United Kingdom.<br>Email: Chris.Fox@uea.ac.uk<br>Telephone (+44) 01603 593583.<br><br>Trial Manager, Dr Simon P Hammond, Norwich Medical School, University of East Anglia, Norwich Research Park, Norwich, Norfolk, NR4 7TJ. United Kingdom.<br>Email: S.Hammond@uea.ac.uk<br>Telephone: (+44) 01603 591460.
Public Title	Care of patients experiencing hip fracture & confusion: PERFECTED CRCT
Scientific Title	Enhancing recovery of patients admitted to acute settings with hip fracture who are identified as experiencing confusion: a multi-centre, cluster-randomised controlled, feasibility trial of the PERFECTED Enhanced Recovery (PERFECT-ER) care versus standard acute care.
Countries of Recruitment	England and Scotland
Health Condition(s) or Problem(s) Studied	Dementia, cognitive impairments and confused patients admitted to acute hospital settings with a hip fracture.
Intervention	The PERFECTED Enhanced Recovery (PERFECT-ER) is a complex intervention. It is a multi-component initiative which aims to improve hospital care for people identified as confused (including but not exclusive to people living with dementia) admitted to an acute hospital ward with a confirmed proximal hip fracture.

For peer review only - http://bmjopen.bmj.com/site/about/guidelines.xhtml

	Active arm: will consist of 5 hospital wards where the PERFECT-ER is being implemented by NHS professionals.<br>Control arm: will consist of 5 hospital wards delivering care as per usual for that setting.
Key Inclusion and Exclusion Criteria for trial	Inclusion criteria:<br>1) Patient must have had a confirmed proximal hip fracture requiring an operation and be aged 60 or older at time of operation;<br>2) Patient has a pre-operative Abbreviated Mental Test Score of 8 or below;<br>3) Patient must have a 'suitable informant' who has a minimum of once a week face-to-face contact and is able, and consents, to provide information on proxy measures (e.g. relative, unpaid or paid carer, care home manager);<br>4) Patient and a suitable informant must be recruited into the trial within 5 days of the hip fracture operation;<br>5) Patient must spend a minimum of 5 days on the study ward.<br><br>Exclusion criteria:<br>1) Decision taken not to have hip surgery;<br>2) Patient not expected to survive beyond 4 weeks.
Study Type	This interventional study is an open trial which will use a cluster randomised controlled design. Ten hospitals will be assigned either active or control status (five hospitals per arm). The ten hospitals will be drawn from five geographical regions. Each region will have two hospitals, one active and control. Intervention assignment will be via a simple randomisation process.
Date of First Enrolment	Anticipated date: 1st November 2016
Target Sample Size	400
Measures	Assessment timeframes are taken from the point after the patient's return from surgery; Baseline (0-5 days post-op, Time 1 (1 month post-op, ± 5 days), Time 2 (3 months post-op, ± 5 days) and Time 3 (6 months post-op, ± 5 days). All measures will be taken at these points unless indicated differently below:<br><br>Patient measures:<br>• Mini-Mental State Examination (MMSE)<br>• Dementia Quality of Life (DEMQOL) self-report version<br>• EuroQol (EQ-5D-5L)<br>• howRwe (Baseline and on discharge from study ward± 5 days)

For peer review only - http://bmjopen.bmj.com/site/about/guidelines.xhtml

	• Timed Up & Go (TUG) (Time 2 only)<br><br>Suitable informant measures:<br>• Bristol Activities of Daily Living Scale (BADLS)<br>• Dementia Quality of Life Proxy (DEMQOL-Proxy)<br>• EQ-5D-5L Proxy<br>• EQ-5D-5L<br>• Client Service Receipt Inventory (CSRI)<br>• Number of days in institutional care – acute hospital; rehabilitation/intermediate care; hospital-at-home/early discharge services<br>• howRthey (Baseline and on patient's discharged from study ward ± 5 days) & Time 1, 2 & 3<br>• Patient's place of residence<br>• Clinical Dementia Rating (CDR) baseline and Time 3)<br>• Informant Questionnaire on Cognitive Decline in the Elderly (IQCODE) (baseline only)<br><br>Other clinical and resource-use measures;<br>• Length of stay in index hospital<br>• Discharge destination from index hospitalisation (T1, T2 & T3)<br>• Mortality;<br>• Hospital re-admissions at T3<br>• Hospital service use (inpatient, outpatient, day and Emergency Department) extracted from patient records<br>• 4AT (baseline and at patients' discharge from study ward ± 5 days)<br>• Charlson Co-morbidity Index (baseline only)

For peer review only

For peer review only - http://bmjopen.bmj.com/site/about/guidelines.xhtml

1.4 Roles and responsibilities

1.4.1 Protocol contributors

Name	Affiliation	Role
Simon Hammond	UEA	Leading and co-ordinating protocol development
Chris Fox	UEA	Protocol development
Lee Shepstone	UEA	Statistical considerations
Catherine Henderson	LSE	Health economic considerations
Erika Sims	NCTU	Operational considerations
Jane Cross	UEA	Operational and intervention considerations
Bridget Penhale	UEA	Ethics and social care considerations
Fiona Poland	UEA	Qualitative protocol considerations
Nigel Lambert	UEA	Protocol development
Anna Varley	UEA	Protocol development
Tamara Backhouse	UEA	Protocol development

1.4.2 Role of trial sponsor and funders

Name	Affiliation	Role
Yvonne Kirkham	UEA	Sponsor representative - legal responsibility for trial conduct
Vasilis Kontogiannis	NIHR	Programme Manager (NIHR)

1.4.3 Trial Team

Name	Affiliation	Role and responsibilities
Chris Fox	UEA	Chief investigator
Lee Shepstone	UEA	Trial statistician
Fiona Poland	UEA	Qualitative lead
Martin Knapp	LSE	Trial Health Economist
Jane Cross	UEA	Intervention Fidelity
Martin Pond	NCTU	Database Development and management
Antony Colles	NCTU	Database Programmer and maintenance
Simon Hammond	UEA	Programme Manager & Research Fellow
Nick Leavey	UEA	Trial Manager
Tamara Backhouse	UEA	Senior Research Qualitative Associate
Catherine Henderson	LSE	Trial Health Economist
Paul Shobrook	UEA	Trial Assistant
Erika Sims	NCTU	NCTU operations linkages
Ann Marie Swart	NCTU	NCTU strategic linkages

For peer review only - http://bmjopen.bmj.com/site/about/guidelines.xhtml

1.4.4 Trial Management Group

Name	Affiliation	Role and responsibilities
Chris Fox	UEA	Chief Investigator
Lee Shepstone	UEA	Statistical lead
Fiona Poland	UEA	Qualitative lead
Jane Cross	UEA	Intervention Fidelity
Bridget Penhale	UEA	Ethics and Social Care
Toby Smith	UEA	Intervention Fidelity
Simon Hammond	UEA	Programme Manager & Research Fellow
Nick Leavey	UEA	Trial Manager
Tamara Backhouse	UEA	Senior Research Associate (Qualitative)
Paul Shobrook	UEA	Trial Assistant
Erika Sims	NCTU	Operational oversight of trial delivery
Antony Colles	NCTU	Data management
Catherine Henderson	LSE	Health Economist
Martin Knapp	LSE	Trial Health Economist

1.4.5 Programme Steering Committee

Name	Affiliation	Role and responsibilities
Cameron Swift	KCL	Independent Chair
Chris Fox	UEA	Chief Investigator
John Young	Univ. Leeds	Site representative
Stephen Jackson	KCL	Site representative
Alasdair MacLullich	Univ. Edinburgh	Scotland lead
Lee Shepstone	UEA	Statistical lead
Fiona Poland	UEA	Qualitative and PPI lead
Jane Cross	UEA	Intervention fidelity
Simon Hammond	UEA	Programme Manager & Research Fellow
Nick Leavey	UEA	Trial Manager
Lynne Chambers	PPI Rep.	Independent member
Sylvia Wallach	PPI Rep.	Independent member
Angela Clayton-Turner	PPI Rep.	Independent member
Prof David Stott	Univ. Glasgow	Independent member
Elizabeth Sampson	UCL	Independent member
Carl May	Univ.Southampton	Independent qualitative
Nick Parsons	Univ. Warwick	Independent statistician

1.4.6 Data Monitoring and Ethics Committee (DMEC)

Name	Affiliation	Role and responsibilities

For peer review only - http://bmjopen.bmj.com/site/about/guidelines.xhtml

Claudia Cooper	University College London	Chair
Andrew Judge	Oxford University	Independent Statistician
George Tadros	Birmingham and Solihull Mental Health Foundation Trust	Independent Member
Marion Shoard	Alzheimer's Society	Public Patient Involvement Member

1.4.7 Other Trial Oversight Groups (Programme Advisory Group)

Name	Affiliation	Role and responsibilities
Cornelius Katona	UCL	Chair
Chris Fox	UEA	Chief Investigator
Lee Shepstone	UEA	Non-independent statistician
Fiona Poland	UEA	Non-independent qualitative
Jane Cross	UEA	Non-independent Intervention Fidelity
Simon Hammond	UEA	Programme Manager and Research Fellow
Sara Gregson	PPI Rep	External Advisor - PPI Representative
Marianne Vincent	PPI Rep	External Advisor - PPI Representative
Dominic Tye	PPI Rep	External Advisor - PPI Representative
Nadine Schofield	Let's Respect	External Advisor – Service Translation
Adrian Treloar	Oxleas NHS Foundation Trust.	External Advisor
Ian Maidment	Aston University	External Advisor
Malaz Boustani	Indiana University	External Advisor
Birgitta Olofsson	Umeå University	External Advisor
Yngve Gustafson	Umeå University	External Advisor

For peer review only - http://bmjopen.bmj.com/site/about/guidelines.xhtml

2 Trial Diagram

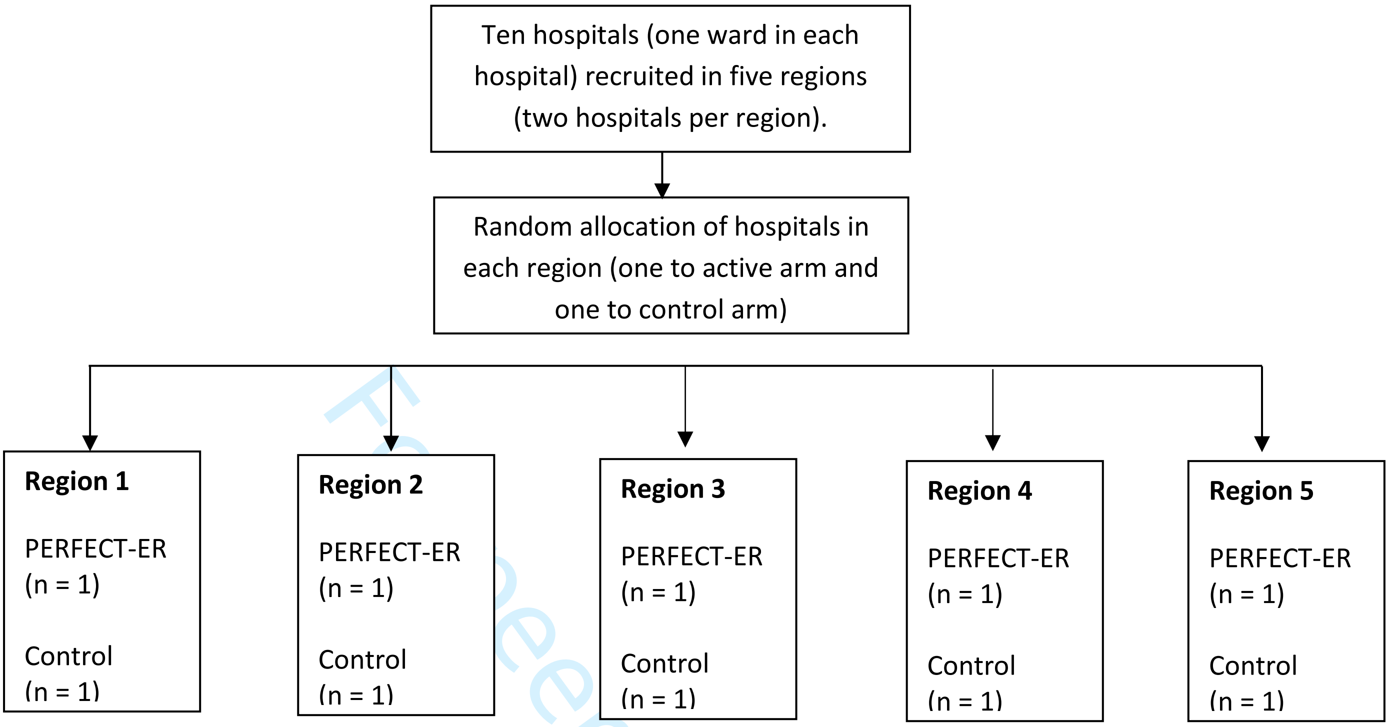

For peer review only

For peer review only - http://bmjopen.bmj.com/site/about/guidelines.xhtml

3 Abbreviations

AE	Adverse Event
AMTS	Abbreviated Mental Test Score
BPT	Best Practice Tariff
CI	Chief Investigator
CRCT	Cluster-Randomised Controlled Trial
CRF	Case Report Form
DMEC	Data Monitoring and Ethics Committee
ERP	Enhanced Recovery Pathway
GCP	Good Clinical Practice
HRA	Health Research Authority
Local PERFECTED Research Worker	Local research staff with honorary and clinical contracts who are a member of host trust's staff. This includes research nurses and facilitators.
NCTU	Norwich Clinical Trials Unit
NHFD	National Hip Fracture Database
NNUH	Norwich and Norfolk University Hospitals
PERFECT-ER	PERFECTED Enhanced Recovery (the intervention)
PI	Principal Investigator

PIN	Participant Identification Number
PIS	Participant Information Sheet
PPL	PERFECTED Process Lead
QA	Quality Assurance
QC	Quality Control
R&D	Research and Development
REC	Research Ethics Committee
SAE	Serious Adverse Event
SAP	Statistical Analysis Plan
SIL	Service Improvement Lead
SSA	Site Specific Approval
TC	Trauma Coordinator
TMF	Trial Master File
TMG	Trial Management Group
TMT	Trial Management Team
ToR	Terms of Reference
PSC	Programme Steering Committee
UEA	University of East Anglia

For peer review only

For peer review only - http://bmjopen.bmj.com/site/about/guidelines.xhtml

4 Introduction

This trial is part of the PERFECTED (Peri-operative Enhanced Recovery hip fracture Care of paTiEnts with Dementia) National Institute for Health Research (NIHR) funded research programme. PERFECTED's aim is to develop and pilot an evidence-based intervention to improve the hospital care of patients living with dementia who experience a hip fracture. This protocol relates to the feasibility study undertaken as part of Work Package 3 (WP3) of PERFECTED, only.

4.1 Background and Rationale

Hip fracture is a common orthopaedic injury amongst older adults. It is estimated that more than 1.2 million individuals suffer hip fracture annually worldwide (Gullberg et al, 1997) and this total is expected to surpass 6 million by the year 2050 (Cooper et al, 1992; Odén et al, 2013). Hip fracture has a significant impact on the health and independence of patients and their families, with an increased risk of functional decline, admission to long-term care institutions and high mortality rates within the first 12 months post-fracture (Seitz et al, 2011). Furthermore, the economic implications for the NHS are significant in both direct and indirect costs (Hernlund et al, 2013).

Older people and their families consistently place high value on hospital care which promotes personalised relationships between patients and staff. This is particularly pertinent for people with impaired cognition or communication difficulties (Alzheimer Society, 2009). People living with dementia have a four-fold increased risk of a hip fracture compared to matched cognitively intact cohorts (Gruneir et al, 2010). It is estimated that 25% of all beds in UK acute hospital wards are occupied by people living with dementia (Royal College of Psychiatrists, 2009). Patients living with dementia – primarily older people, are highly susceptible to insensitive care as complex needs in relation to their memory, thinking, orientation, comprehension, calculation, learning capability, language and judgment are often unrecognised and unaddressed (Alzheimer Society, 2009). Engagement and progress with post-operative hip fracture rehabilitation can also be compromised for patients living with dementia. Data suggest that clinical outcomes (incidence of delirium, mortality and post-operative complication rates) are poorer for people living with dementia following hip fracture when compared to cognitively intact individuals (Smith et al, 2013).

For peer review only - http://bmjopen.bmj.com/site/about/guidelines.xhtml

4.1.2 Enhanced Recovery Pathway (ERP)

The process of "Enhanced Recovery" has been used in the UK since the early 2000s (Department of Health 2013a). It is an integrated, multi-modal, evidence-based approach that enables acute hospital patients to recover from surgery, treatment or illness more effectively and leave hospital sooner. Enhanced Recovery Pathways (ERP) are becoming standard practice for many elective surgical operations. ERP for elective surgery are becoming more common as clinical indictors show promise. However, ERPs for *acute* hip fracture are much rarer.

4.1.3 The PERFECTED Research programme

Hip fracture and cognitive impairment (including but not exclusively, people living with dementia) are recognised as common comorbidities. This has led to calls for hip fracture patients experiencing dementia (suspected and confirmed) to have a specific treatment pathway, to acknowledge the differences in presentation and care needs of this patient group (McGilton et al, 2013).

Informed by clinical knowledge and learning from the previous stages of our research, we note that the PERFECTED Enhanced Recovery process intervention (henceforth, PERFECT-ER) is likely to improve care delivery to patients with presumed dementia and other cognitive impairments such as delirium. For such reasons, these terms are coalesced here under the term "*confusion*". For PERFECT-ER to make a positive impact on the care of older confused adults, it needs to take a broad approach. Hip fracture is the most common orthopaedic injury amongst older people and the majority of patients admitted with a hip fracture who are experiencing confusion do not arrive with a confirmed dementia diagnosis. We will include patients with known or presumed dementia of any severity, who incur a fractured hip, admitted from any setting. In this way we will employ the term confusion in order to reflect the real-world complexity of the acute hospital environment into which PERFECT-ER will be introduced.

4.3 Objectives

The ultimate objective of this feasibility trial is to inform a definitive evaluation of the PERFECT-ER intervention. To prepare for this definitive large scale trial the current study has the following research objectives:

For peer review only - http://bmjopen.bmj.com/site/about/guidelines.xhtml

- Recruit 10 acute hospital wards (one ward per hospital), across five regions: East Anglia, East Midlands, London, Yorkshire and Scotland, (two hospitals per region), assigning one intervention and one control hospital per region;
- Implement the PERFECT-ER intervention in five active sites, one per region, and ensure sites achieve the opening PERFECT-ER adherence target and continue to report adherence issues to inform the definitive trial bid;
- Recruit and collect data from 40 patients (and their relevant suitable informant(s)) per site via in-hospital assessments, accessing patient's medical records and follow-up assessments at 1, 3 and 6 months post-op;
- Recruit up to 5 patients and up to 5 suitable informants from each active site who have experienced the PERFECT-ER intervention to take part in interviews;
- Recruit a range of NHS staff (service managers and clinicians with experiences implementing and/or using the PERFECT-ER) to take part in qualitative focus groups or interviews where appropriate;
- Capture recorded variation between control wards and active wards by randomly sampling 10 sets of case notes per control ward (this will be done in active sites as part of the PERFECT-ER adherence monitoring);
- Generate and capture knowledge to inform a definitive trial, specifically:
  - Primary outcome selection;
  - Recruitment and retention rates;
  - Identification of intervention delivery and outcome data collection difficulties;
  - Provide an estimate of completeness of outcome data;
  - Potential intervention effectiveness and intra-class correlation coefficients (ICC) for efficacy endpoints;
  - Create evaluation of potential economic benefits of intervention and whether staff behaviours have changed over time in the active sites;
  - Produce a narrative account of the implementation experiences of the intervention via qualitative methods.

4.4 Trial Design

This multi-centre cluster randomised controlled trial is being undertaken in 10 acute hospital wards in 10 differing hospitals, one ward per hospital. The unit of randomisation is therefore the cluster, which in this case is the hospital site. The trial

For peer review only - http://bmjopen.bmj.com/site/about/guidelines.xhtml

has two arms. The active arm features five study hospitals in which the intervention (PERFECT-ER) is being implemented. In the control arm, hospitals will continue to deliver 'standard care'. The 10 hospitals are in five different regions: each region will have one hospital in the active and control arm. Masking of the patients, suitable informants and staff delivering treatments is not possible. Patient, suitable informants and NHS professionals may know to which arm they have been allocated, making this an open unblinded trial. Amongst the trial team, statistical analysis will be undertaken via a subgroup-blind approach (blinded to trial arm and cluster).

Because the PERFECT-ER intervention is a ward-level service delivery improvement initiative, including components such as staff training, consent to treatment is not required. It is important to highlight that all patients may be exposed to the 'effects' of the service delivery intervention. However, permission will be sought to enable the research team to access routinely collected clinical data and to generate specific research data in-hospital and at follow-up. In the case of patients with capacity (who meet all the inclusion and none of the exclusion criteria) and their suitable informants, we will seek explicit consent to participate. In the case of the patients lacking capacity to give informed consent, we will seek consent from a relevant legal representative (Scotland) and advice from an appropriate consultee (England). As numerous informant (or proxy) measures will be used, consent will be sought from those contributing this information about the patient. This population is referred to in this protocol as 'suitable informants'. These are individuals who have a minimum of once a week face-to-face contact with the patient and whom are able, and consent, to provide information on proxy measures (e.g. relative, unpaid or paid carer, care home manager).

For peer review only - http://bmjopen.bmj.com/site/about/guidelines.xhtml

5 Methods

5.1 Site Selection

The trial sponsor has overall responsibility for site and investigator selection and has delegated this role to the Chief Investigator and NCTU.

5.1.1 Study Setting

The study setting is acute trauma ward environments in hospitals across England and Scotland to which individuals suffering Neck of Femur (NOF) fractures are admitted. NHS hospitals in each locality have a variety of titles for the types of service delivered and teams responsible for service delivery, but all provide acute fracture care. Given the different names used in clinical practice, for the purpose of the protocol, wards will be referred to as 'acute trauma wards'.

5.1.2 Site Eligibility Criteria

Once a site has been assessed as being suitable to participate in the trial, the trial team will provide the site with a copy of this protocol and its relevant annexes. To participate in the trial, investigators and trial sites must fulfil a set of criteria that have been agreed by the Trial Management Group (TMG) prior to randomisation and that are defined below;

- Sites must not have participated in the previous 'PERFECTED WP2: Implementing optimised hospital care' research leading to the development and refinement of PERFECT-ER;
- Sites have an average monthly admission of at least 12 individuals suffering a proximal hip fracture requiring an operation who have a pre-op Abbreviated Mental Test Score (AMTS) of 8 or below (England) or a 4AT of 1 or above (Scotland)[1] in the last 12 available calendar months;
- Sites are able to provide PERFECTED trial team with contextual ward level data (comprising Best Practice Tariff scores, number of falls, pressure ulcers, deaths and safeguarding incidents) in the last 12 available calendar months;

[1] In our Scottish sites the 4AT is a more widely used equivalent. In this way patients with a 4AT on admission of 1 or above and a confirmed proximal hip fracture will be screened pre-operatively by a local PERFECTED research worker trained in administering the AMTS. Should this patient score 8 or below on the AMTS they will be approached about taking part in the study. Should they not they will not be approached.

For peer review only - http://bmjopen.bmj.com/site/about/guidelines.xhtml

- Sites are able to complete a ward profile sheet at opening, 50% and 100% recruitment;
- Sites have named PERFECTED research workers[2] that are available to be the main contact with patients and suitable informants in order to take informed consent, gain legal representative/consultee agreement and complete all trial CRFs including all follow-ups;
- Site personnel agree to undertake AMTS standardisation training on an as required basis;
- Sites have capacity to release Band 6 member of existing ward staff to operate as a Service Improvement Lead funded by the trial;
- Active sites achieve an acceptable opening PERFECT-ER adherence score (score to be informed by Work Package 2, circa Summer 2016) prior to opening recruitment;
- Active sites able to make relevant staff available for qualitative sub-study.

Trial sites meeting eligibility criteria and that are accepted by the TMG as being suitable to recruit to the trial will be issued with the Trial Master File (TMF) documentation to use when applying for Site-Specific Approval (SSA).

5.1.2.1 *Principal Investigator's (PI) Qualifications and Agreements*

The investigator(s) must be willing to sign a NCTU Clinical Trial Agreement or an Investigator Agreement to comply with the trial protocol (confirming their specific roles and responsibilities relating to the trial, and that their site is willing and able to comply with the requirements of the trial). This includes confirmation of appropriate qualifications, familiarity with the protocol, agreement to comply with the principles of GCP, to permit monitoring and audit as necessary at the site, and to maintain documented evidence of all relevant staff at the site that have been delegated significant trial related duties.

5.1.2.2 Resourcing at site

The investigator(s) should be able to demonstrate a potential for recruiting the required number of suitable subjects within the agreed recruitment period (i.e. the investigator(s) and site regularly treat(s) the target population). They should also have

[2] Local research staff with honorary and clinical contracts who are a member of host Trust's staff. This includes research nurses and facilitators.

For peer review only - http://bmjopen.bmj.com/site/about/guidelines.xhtml

an adequate number of qualified staff and facilities available for the foreseen duration of the trial to enable them to conduct the trial properly and safely.

Sites will be expected to complete a delegation of responsibilities log and provide staff contact details. The site should have sufficient data management resources to allow prompt data return to NCTU.

5.2 Site approval and activation

On receipt of the signed Clinical Trial Agreement or Investigator Agreement, approved delegation of responsibilities log and staff contact details, written confirmation will be sent to the site PI. The Trial Manager or delegate will notify the PI in writing of the plans for site initiation.

The site must conduct the trial in compliance with the protocol as agreed by the Sponsor and which was approved by the Research Ethics Committee (REC). The Site PI or delegate must document and explain any deviation from the approved protocol, and communicate this to the trial team at NCTU.

A list of activated sites may be obtained from the Trial Manager.

5.3 Participants

5.3.1 Eligibility Criteria

The inclusion and exclusion criteria below have been informed by qualitative (Work Package 1) and action research (Work Package 2) findings and have been comprehensively discussed amongst the academic and clinical multidisciplinary PERFECTED Trial Management Group.

5.3.2 Participant selection

There will be no exceptions (waivers) to eligibility requirements. Questions about eligibility criteria should be addressed prior to attempting to recruit participants.

The eligibility criteria for this trial have been carefully considered and are the standards used to ensure that only appropriate participants are entered. Participants not meeting the criteria should not be entered into the trial. This ensures that the trial results can be appropriately used to make future service provision decisions for other people with similar diseases or conditions. It is therefore vital that exceptions are not made to these eligibility criteria.

For peer review only - http://bmjopen.bmj.com/site/about/guidelines.xhtml

5.3.2.1 Patient participant inclusion and exclusion criteria (trial)

Patient participants will be considered eligible for enrolment in this trial if they fulfil all the inclusion criteria and none of the exclusion criteria as defined below.

Inclusion criteria:

1) Patient must have had a confirmed proximal hip fracture requiring an operation and be aged 60 or older at time of operation;

2) Patient has a pre-op AMTS of 8 or below (including those with 0 because of an inability to answer questions)[3];

3) Patient must have a 'suitable informant' (e.g. relative, unpaid or paid carer, care home manager) who has a minimum of once a week face-to-face contact with the patient and is able, and consents to, provide information on proxy measures;

4) Patient and a 'suitable informant' must be recruited into the trial within 5 days of the hip fracture operation;

5) Patient must spend a minimum of 5 days on the study ward.

Exclusion criteria:

1) Decision taken not to have hip surgery;

2) Patient not expected to survive beyond 4 weeks.

5.3.2.2 Suitable informant participant inclusion and exclusion criteria (trial)

Suitable informant participants will be considered eligible for enrolment in this trial if they fulfil all the inclusion criteria and none of the exclusion criteria as defined below.

Inclusion criteria:

1) Individual has a minimum of once a week face-to-face contact with the patient;
2) Individual is able, and consents to, provide information on proxy measures.

Exclusion criteria:

1) Individual not over 16 years of age.

[3] In our Scottish sites patients with a 4AT on admission of 1 or above and a confirmed proximal hip fracture will be screened pre-operatively by a local PERFECTED research worker trained in administering the AMTS. If patient scores 8 or below on the AMTS they are eligible to be recruited.

For peer review only - http://bmjopen.bmj.com/site/about/guidelines.xhtml

5.3.3 Consenting patient and suitable informant participants into the trial

Informed consent to enter into the trial must be obtained from patients. In cases where written informed consent cannot be obtained, verbal consent can be taken (for example a patient with extremely poor eyesight or wrist/arm fractures). However this must be witnessed and fully documented (Declaration of Helsinki, 2008; paragraph 22). Informed consent must also be obtained from a 'suitable informant' (someone who fits the suitable informant inclusion criteria and consents to provide data for the trial themselves) who will provide proxy information about the patient. Again for suitable informants in instances where written informed consent cannot be obtained, verbal consent can be taken but must be witnessed and fully documented (Declaration of Helsinki, 2008; paragraph 22). It is important to note that suitable informant participants can be replaced as the study progresses should they withdraw or their contact with the patient participant change meaning they are no longer eligible. In such instances, a combination of telephone and/or postal recruitment (initial contact may be made verbally with an agreement to send out relevant study information with this information returned by the interested potential suitable informant participant by post) may be necessary for this participant group. Regardless of recruitment mechanisms, eligible suitable informant participants will be given as long as they need to consider study information and have any questions answered fully.

Following an initial witnessed verbal consent in either participant group, written consent should be sought in most instances. For example when a suitable informant visits a patient participant on the Ward, or when a patient has recovered the use of her/his upper limb. The eliciting of informed consent will only occur after explanation of the aims, methods, benefits and potential hazards of the trial and BEFORE any trial-specific research procedures are performed.

The provision of informed consent to participate in the trial includes consenting to the initial trial measures, and trial follow-ups as described in the protocol and the relevant participant information sheet. Patients will be asked to consent to their medical records being accessed as part of the trial and to agree that they are happy to be approached regarding their possible involvement in the qualitative component of the trial. For those approached to take part in the qualitative component, additional consent will be gained

For peer review only - http://bmjopen.bmj.com/site/about/guidelines.xhtml

(see section 9 for more details). Suitable informants will be asked to consent to provide data about their perspectives on various aspects of the patient participant's memory and everyday life. Suitable informants will also agree to be approached about possible involvement in later qualitative components of the trial (see section 9). As with patients, if selected for qualitative components, suitable informants will be consented accordingly.

Patient and suitable informant information and consent forms have been designed following HRA guidance in both England and Scotland, each have had input from relevant Public Patient Involvement (PPI) representatives to ensure that they remain accessible and are fit for purpose. The main potential ethical issue in this study is that the patient's cognitive status may interfere with their ability to give informed consent.

Where possible, informed written consent will be obtained from patients entering the trial. However, some potentially suitable patients may lack the necessary mental capacity to give informed written consent. The aims of this trial are incompatible with only enrolling patients with mild or moderate confusion. It is important to ensure that the findings are broadly applicable and are therefore able to inform clinical practice and the future definitive trial. In this way, for a representative patient group to be recruited, the current trial must include participants who lack capacity to give informed consent. In this situation, the patient's agreement to participate will still be obtained to their best level of understanding (in line with legislative frameworks in England and Scotland). Recruitment will not proceed if the patient appears to object or show significant distress. Local PERFECTED research workers are trained in making an individualised capacity assessment of patients in line with local legislative frameworks (Mental Capacity Act 2005 (England) and Adults with Incapacity (Scotland) Act 2000).

5.3.3.1 Recruiting patients assessed not to have capacity (England)

In English trial sites, in line with Principle 1 of the Mental Capacity Act 2005 (England), a potential patient participant will be assessed as having capacity until it is established otherwise. When this is the case and all practical steps to help them to engage in the decision making process have been tried (Principle 2 of Mental Capacity Act 2005), the site trial team will seek a *personal consultee*. This person will be;

For peer review only - http://bmjopen.bmj.com/site/about/guidelines.xhtml

- Someone who is engaged in care for the participant (not professionally or for payment) or is interested in his/her welfare and is prepared to be consulted. This may be a family member, carer or close friend, or attorney acting under Lasting Power of Attorney. This person can also act as a suitable informant if they fulfil the inclusion criteria.

If a potential personal consultee is not available or declines to take part he/she may suggest another person to act as a personal consultee, alternatively a *nominated consultee* will be sought. This should be;

- A person independent of the research study and who is willing to be consulted about the participation of a person who lacks capacity where reasonable steps have been taken to identify a personal consultee. This may be someone who knows the patient in a professional capacity e.g. social worker, ward staff member, paid carer or GP, provided they have no connection to the research study.

If, at a later point during the trial or follow-up procedures, the patient participant is assessed by a local PERFECTED research worker to have regained capacity (a possibility in the case of some cognitive impairments such as delirium), he/she will be approached about continuing to participate in the study and asked to give informed consent. Should they chose to withdraw from the study at this point we will ask if we can keep the data collected thus far.

5.3.3.2 Involving patients who may lose capacity (England)

In the consenting process, patients with capacity will be asked to share their wishes about their ongoing participation even if they lose capacity at a later stage during the study. This is common in the case of some cognitive impairment such as dementia, where patients may develop delirium. Patient participant wishes will be discussed with the approached consultee (personal or nominal). Therefore patients who lose capacity after consenting will remain in the research and follow-up procedures continue unless;

- The participant appears to object;
- Continuation would be contrary to an advanced statement made by the participant (we acknowledge that this should be rare);

For peer review only - http://bmjopen.bmj.com/site/about/guidelines.xhtml

- We are unable to locate a consultee;
- The approached consultee (personal or nominated) advises they would like the participant to be withdrawn.

If any of the above applies, the patient participant will be removed from the study and data collection from their suitable informant would also cease. However, data collected up to this point, which the participant consented to us collecting, will be kept by the study as detailed in the relevant PIS, consent forms or consultee declaration form.

5.3.3.3 Recruiting patients assessed not to have capacity (Scotland)

In Scottish trial sites, in line with Adults with Incapacity (Scotland) Act 2000), where a potential patient participant is assessed not to have capacity, a welfare guardian, welfare attorney or nearest relative will be sought and asked to consent in relation to participation in research (this person will be henceforth known as a *legal representative* for the purposes of this protocol). This procedure will be undertaken once;

- An assessment of capacity has been made in relation to the specific decision regarding the research participation;
- Any barriers to participating in the consent process have been removed, for example a more accessible PIS;
- The local PERFECTED research worker feels the individual cannot retain information long enough to use it in order to arrive at a decision.

Legal representatives may be involved in conversations regarding the consenting process, however they will be asked to differentiate between expressions of their own views and reporting the known values and/or views of the potential patient participant. If the potential participant is unable to consent for themselves then consent will be sought on their behalf from a suitable legal representative.

If, at a later point during the trial or follow-up, the patient participant is assessed to have regained capacity, a possibility in the cases of some cognitive impairments such as delirium, the patient participant will be approached about their continued participation in the study and informed consent will be sought. Should they choose to withdraw we will ask if we can keep the data collected from them up until this point.

For peer review only - http://bmjopen.bmj.com/site/about/guidelines.xhtml

5.3.3.4 Recruiting patients who may lose capacity (Scotland)

In the consenting process, patients with capacity will be asked to share their wishes about their ongoing participation even if they lose capacity at a later stage during the study. This is common in the case of some cognitive impairment such as dementia, where patients may develop delirium. Patient participant wishes will be discussed with the approached legal representative. Therefore, patients who lose capacity after consenting will remain in the research and follow-up procedures will continue unless:

- The participant appears to object;
- Continuation would be contrary to an advance statement made by the participant (we acknowledge that this should be rare);
- We are unable to find a suitable legal representative;
- The approached legal representative (a guardian, welfare attorney or nearest relative) advises they would like the participant to be withdrawn.

If any of the above applies, the patient participant will be removed from the study and data collection from their suitable informant would also cease. However, data collected until this point in time, which the participant will have consented to us collecting, will be kept by the study as detailed in the relevant PIS, consent forms or legal representative form.

For peer review only - http://bmjopen.bmj.com/site/about/guidelines.xhtml

5.3.4 Patient and suitable informant recruitment diagram

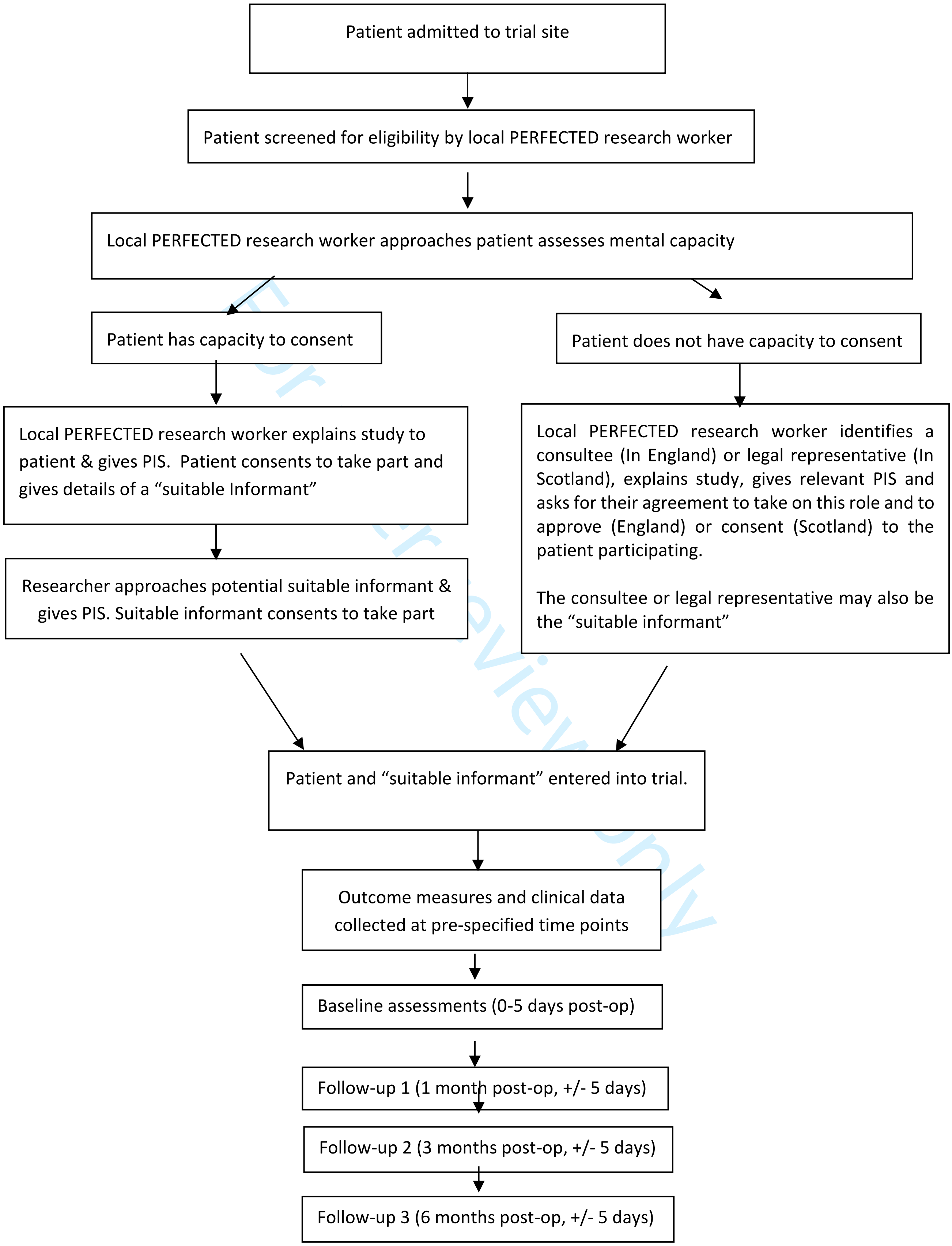

For peer review only - http://bmjopen.bmj.com/site/about/guidelines.xhtml

5.3.5 Withdrawal of patient participant and/or suitable informant

The right of a participant (patient or suitable informant) to discontinue participation without giving reasons will be respected. The patient and suitable informant will remain free to withdraw from the trial at any point without giving reasons and without prejudicing their further treatment. However we will ask for a reason to help inform the definitive trial bid. They will be provided with a contact point where either the patient or suitable informant may obtain further information about the trial.

In the event of a suitable informant withdrawing consent during the trial phase (for whatever reason), an alternative suitable informant will be sought. In instances where the patient participant is assessed to have capacity, the patient participant will continue in the study even if a suitable informant cannot be found. Although, efforts to locate a suitable informant should continue at each data collection point.

In instances where the patient participant is assessed to have lost capacity (either acutely or chronically), if a suitable replacement informant cannot be found in a timely manner (prior to the next data collection point) no further information will be collected from the patient participant and they will be informed that we need to withdraw them from the trial. In cases where the patient participant was assessed to have lost capacity and their enrolment/continued participation was assisted via the involvement of a consultee (England) or Legal Representative (Scotland), this person will also be informed of the need to withdraw the patient participant and the reasons for this. If a patient participant has been withdrawn (for whatever reason) the relevant suitable informant cannot continue to contribute data about that individual patient participant. However, in cases where a suitable informant is providing data about more than one participant (for example in the case of a care home manager), the suitable informant will continue to provide data about the remaining patient(s) participating in the trial.

Consent will be re-sought if new information becomes available that affects the patient participant's consent in anyway. A local PERFECTED research worker will provide this new information via a revised information sheet and the patient will be asked to sign an updated version of the relevant consent form. Consent forms and patient information sheets will be approved by the ethics committee prior to their use.

The Site Principal Investigator (PI) retains overall responsibility for the informed consent of participants at their site and will ensure that any person delegated

For peer review only - http://bmjopen.bmj.com/site/about/guidelines.xhtml

responsibility to participate in the informed consent process is duly authorised, trained, clinically appropriate and competent to participate according to the approved protocol, principles of Good Clinical Practice (GCP) and Declaration of Helsinki (2008). The PI also takes responsibility for ensuring that all participants are protected and participate voluntarily in an environment free from coercion and undue influence.

5.3.6 Early stopping of follow-up

If a patient participant chooses to discontinue their participation in the trial, this view will be respected, and the patient participant and relevant suitable informant withdrawn. NCTU should be informed of the withdrawal in writing using the appropriate trial documentation. Although not obliged to give a reason for discontinuing their involvement, a sensible effort should be made to establish this reason, whilst remaining fully respectful of the participant patient's rights. In cases where the participant has had the capacity to consent, data already collected will be kept and included in analyses. In cases where a patient participant has recovered capacity and choses to withdraw, we will seek to keep all data collected up until the point they withdraw.

If a suitable informant chooses to withdraw their participation in the trial, this will be respected. Although not obliged to give a reason for discontinuing their trial involvement, a reasonable effort should be made to establish this reason, whilst remaining fully respectful of the suitable informant participant's rights. All data collect from them until this point will be kept. In the case of suitable informant withdrawal, an alternative suitable informant shall be sought. Strategies to accomplish this will include asking the patient and/or withdrawing suitable informant to recommend an alternative suitable informant.

5.3.7 Patient dies during trial

In the unfortunate circumstances that the participant dies during their involvement in the trial, their suitable informant and/or their legal representative (Scotland) or an appropriate consultee (England), have been asked to inform their local PERFECTED research worker when possible to avoid undue distress. This is detailed on the relevant information sheets. Additionally, prior to contacting patient and suitable informant

For peer review only - http://bmjopen.bmj.com/site/about/guidelines.xhtml

participants, local PERFECTED research workers[4] will check the participating Trusts electronic records to attempt to reduce the possibility of contacting deceased patients and those close to them. Patient participants who stop trial follow-up early will not be replaced. However, where appropriate, replacement suitable informant participants will be sought.

5.3.8 Loss to Follow-up

Attempts will be made to follow up all patients participants. However if patients are lost to follow up there is no intention to replace them.

5.3.9 Participant moves

During the course of the patient participant involvement in the trial it is likely that they will experience a change of living address. Every effort should be made to facilitate patient participants' continued involvement in the trial. This will include local PERFECTED research workers liaising closely with institutional settings such as subacute/rehabilitation hospital settings and care homes to ensure appropriate permissions have been obtained to assist continue patient participant involvement. Clearances required are likely to differ but seek local PERFECTED research workers will organised Letters of Access/Letters of Permission if required organisations

5.3.9.1 Co-enrolment Guidance

Patients will not be permitted to be co-enrolled in any other hospital based intervention studies or any Clinical Trials of Investigational Medical Product. This will be assessed by questioning the patient, their formal/informal carers, suitable informant and as part of the screening exercise. PERFECTED 'patient' stickers' will be provided in consent packs. The relevant Participant Information Sheets (PIS) will also state that co-enrolment is not permitted. Patient stickers will be added to the notes of patients recruited to the trial (or a file note in accordance with local Trust regulations) to prevent the recruitment of the same patient twice into the study. The screening log will record details of rescreening in the event that PERFECTED screening failures occur.

5.3.9.2 Screening procedures and pre-consent

Because this is a Cluster-Randomised Controlled Trial (CRCT) investigating the mode of service delivery, patients admitted to hospitals in either active or control arm will still

[4] Local PERFECTED research workers are local research staff with honorary and/or clinical contracts who are a member of host trust's staff. This includes research nurses and facilitators.

For peer review only - http://bmjopen.bmj.com/site/about/guidelines.xhtml

be cared for according to the care delivery which is already in situ. In this way, regardless of consent status, patients in the active arm will still be cared for as per the contents of the PERFECT-ER and those in the control as per usual. As such, procedures that may be performed in advance of informed consent being obtained are those that will be performed as part of routine practice For example, a pre-operation (pre-op) AMTS is collected from all patients over 60 years of age admitted to acute hospitals across England in line with the National Hip Fracture Database (NHFD) Best Practice Tariff (BPT). As cognitive screening for acute patients aged 60 or over are common practice and relevant to the study's target population, this age has been selected as the lower age limit and inclusion threshold.

In our Scottish sites the 4AT is a more widely used equivalent to the AMTS. In this way patients with a 4AT on admission of 1 or above and a confirmed proximal hip fracture will be screened pre-operatively by a local PERFECTED research worker trained in administering the AMTS. Should this patient score 8 or below on the AMTS they will be approached about taking part in the study.

5.5 Interventions

The study setting is ten acute hospital trauma wards, eight in England and two in Scotland. There are two trial arms: (i) active (PERFECT-ER) and (ii) control (standard care).

5.5.1 Active Arm

The PERFECTED Enhanced Recovery Pathway (PERFECT-ER) is a multicomponent service improvement intervention with a systematic implementation process. PERFECT-ER integrates current best practice evidence of hospital-based dementia care delivery with current best practice evidence of the admission, pre-operative, post-operative, rehabilitation and discharge management of acute hip fractures. PERFECT-ER aims to overlay on existing Neck of Femur (NOF) pathways, whilst deploying initiatives to improve care delivery to those patients who are confused in a manner relevant to the sites' existing strengths and weaknesses.

In essence the intervention consists of a PERFECT-ER checklist and manual regarding how best to implement it in real life ward settings. Both have been refined by earlier PERFECTED work packages in partnership with a range of stakeholders. The checklist features organisational and patient level items. Patient level items (which

For peer review only - http://bmjopen.bmj.com/site/about/guidelines.xhtml

are the checklists main focus) are divided up into phases of the patient pathway with ‘care themes’ made up of items from across the pathway orientating around a particular element of providing care. In this way wards can highlight particular strengths and areas for improvement and create localised action plans to facilitate improved scores on the PERFECT-ER checklist. Sites in the active arm will be given the manualised PERFECT-ER service improvement intervention (checklist and manual) three months prior to the commencement of recruitment (circa November 2016) and resource support to implement it.

PERFECT-ER will be implemented using the NHS Service Improvement model (plan-do-study-act) via the recruitment of a ward-based ‘champion’ also known as a Service Improvement Lead (SIL). Each active site will have SIL resource given to it, paid for by the research grant. This member of staff will be recruited from a member of the study site’s existing staff team. Each active site will receive 50% full-time equivalent (FTE) costings for a NHS Band 6 member of staff for the first three months when the SIL will be championing the implementation and adherence to the PERFECT-ER intervention. Once the site has achieved the adherence on the PERFECT-ER intervention, the SIL resource will reduce to 20% FTE for the duration of the 10 month recruitment window. It is the SIL’s job to champion PERFECT-ER and to implement change. This role will ensure that the active sites can achieve acceptable PERFECT-ER adherence levels prior to the start of recruitment, and, due to the pilot nature of the current trial, monitor adherence to the PERFECT-ER tool during this recruitment window.

To assist the SIL role and to create buy-in at a clinical level, a PERFECTED Process Lead (PPL) will be assigned by the active sites from the existing clinical team. This equates to 0.01% FTE per annum. This will equate to an hour a week for the initial three months to assist the SILs implement the PERFECT-ER, reducing to around 1 hour per month during the 10-month recruitment period. These NHS employees will work closely with the SIL at their site to implement changes at ward-level and support the review of emerging issues.

Although implementation will be bespoke locally, the intervention is described below in accordance to headings used for describing complex interventions (Hoffman, 2014).

For peer review only - http://bmjopen.bmj.com/site/about/guidelines.xhtml

5.5.1.1 TIDieR Framework

Brief name	PERFECT-ER checklist and implementation manual
Why	Older patients who break their hip are recognised as exceptionally vulnerable to experiencing confusion (including but not exclusively, dementia and/or delirium) before, during or after their acute admission. This group are at a high risk of serious complications, linked to delayed recovery and higher mortality post-operatively. Specific treatment pathway which acknowledge the differences in presentation and care needs of this patient group (McGilton et al, 2013) are likely to improve clinical and process outcomes.
Materials	An enhanced recovery intervention “PERFECT-ER” (checklist and implementation manual) will be given by the research team to a ward based Service Improvement Lead (SIL) who will work with ward colleagues to implement changes to practice based on scores derived from the PERFECT-ER checklist. PERFECT-ER checklist comprises of items aimed at optimising care for acute hip fracture patients experiencing confusion from admission to discharge from the study ward. Developed as a result of previous PERFECTED work packages, the PERFECT-ER checklist and implementation manual are not available due to contamination.
Procedures	PERFECTED Service Improvement Leads (existing Band 6 member of ward staff) and PERFECTED Process Leads (member of clinical team) have 3 months to implement and achieve a checklist score which meets an acceptable PERFECT-ER adherence threshold (this will be informed by work completed in July 2016). Ward checklist scores will be derived from deploying the checklist against a selected number patient’s notes (this will be informed by work completed in July 2016).
Who	Each active site will get resource to appoint a Service Improvement Lead (SIL). This member of staff will be recruited from the study site’s existing staff team. Each active site will receive 50% full-time equivalent (FTE) costings for a NHS Band 6 member of staff for the first 3 months when the SIL will be championing the implementation and checking adherence to the PERFECT-ER intervention. The SIL resource will reduce to 20% FTE for duration of the 10 month recruitment window. It is SIL’s job to champion PERFECT-ER and to implement change. To assist the SIL role and to create buy-in at a clinical level, a PERFECTED Process Lead (PPL) will be assigned by the active sites from the existing clinical team. This is approximately 0.01% FTE per annum. This will equate to an hour a week for the initial three months to assist the SILs implement the PERFECT-ER, reducing to around 1 hour per month during the 10-month recruitment period.

For peer review only - http://bmjopen.bmj.com/site/about/guidelines.xhtml

How	Behaviour change will be facilitated by the SIL and PPL via the NHS Service Improvement model (plan-do-study-act). The SIL will use the checklist to create an initial ward score (baseline). As it overlays existing Neck of Femur (NoF) pathways, the checklist allows those on the ward to recognise areas of strength to consolidate whilst highlighting areas for improvement. The SIL and PPL will feedback these scores to the ward staff team. These will be discussed with action plans created based on these ward-specific discussions and insights. Action plans will then be implemented by the SIL, PPL and ward staff before the SIL uses the checklist again to review progress or problems, before again feeding back to the ward teams. This cycle will be repeated numerous times to reach the acceptable PERFECT-ER checklist adherence level. Adherence will be monitored across the recruitment period.
Where	The checklist and manual will be used on active study wards only. Any action plan points raised could take place in any area of the ward.
When	The implementation of PERFECT-ER will begin on each active ward up to 3 months prior to recruitment opening (estimated implementation start date August 2016 and recruitment opening November 2016) and continue through the 10 month data collection window (recruitment closing circa September 2017).

5.5.2 Control arm

Control is treatment as usual. All patients will be accessing care as normal for that setting.

5.5.3 Adherence monitoring

For the active sites to be considered 'active' they need to achieve an initial adherence score on the PERFECT-ER. This score is generated by the active arm SIL and monitored across the 10 month recruitment period.

5.6 Outcomes

This feasibility study will not have a primary outcome. Instead this study will provide information to inform the selection of the primary outcome for the definitive HTA bid which will follow the closure of this trial. All outcome assessment timeframes are taken from the point after a patient returns from surgery (i.e. during post-op)[5]. Measures will be collected at baseline[6] (0-5 days post-op) and follow-up: time 1 (1 month ± 5 days), time 2 (3 months ± 5 days), and time 3 (6 months ± 5 days). Follow-up measures will be obtained at the patients' place of residence by a local PERFECTED research worker.

[5] For details regarding qualitative procedures, please see Section 9.
[6] Where possible data will be collected in the first 24 hours post-op.

For peer review only - http://bmjopen.bmj.com/site/about/guidelines.xhtml

Patient-completed measures:

- Mini-Mental State Examination (MMSE) collected at baseline, 1 month, 3 months and 6 months (follow-ups all ± 5 days);
- Dementia Quality of Life Measure (DEMQOL) self-report version, collected at baseline, 1 month, 3 months and 6 months (follow-ups all ± 5 days);
- EuroQol (EQ-5D-5L) self-completed, collected at baseline, 1 month, 3 months and 6 months (follow-ups all ± 5 days);
- howRwe Questionnaire, collected at baseline and on patient's discharge from the study ward (discharge ± 5 days);
- Timed Up & Go (TUG)[7] collected at 3 months (follow-up ± 5 days).

Suitable Informant-completed measures:

- Bristol Activities of Daily Living Scale (BADLS), collected at baseline, 1 month, 3 months and 6 months (follow-ups all ± 5 days);
- Dementia Quality of Life Measure Proxy (DEMQOL-Proxy), collected at baseline, 1 month, 3 months and 6 months (follow-ups all ± 5 days);
- EuroQol (EQ-5D-5L) by proxy on behalf of patient versions, collected at baseline, 1 month, 3 months and 6 months (follow-ups all ± 5 days);
- EuroQol (EQ-5D-5L) (carer completed self-report), collected at baseline, 1 month, 3 months and 6 months (follow-ups all ± 5 days);
- Client Service Receipt Inventory (CSRI), collected at baseline, 1 month, 3 months and 6 months (follow-ups all ± 5 days);
- Number of days in temporary or permanent institutional care (residential care home, nursing home, continuing care/dementia care unit) at 1 month, 3 months and 6 months (follow-ups all ± 5 days);
- howRthey Questionnaire, collected at baseline, on patients discharge from the study ward (discharge ± 5 days) 1 month, 3 months and 6 months (follow-ups all ± 5 days);
- Patients place of residence at baseline, 1 month, 3 months and 6 months (follow-ups all ± 5 days);

[7] TUG test is performed twice (one untimed practice trial and one timed). The score in seconds is used as the outcome.

For peer review only - http://bmjopen.bmj.com/site/about/guidelines.xhtml

- Clinical Dementia Rating (CDR) baseline and 6 months (follow-up ± 5 days);
- Informant Questionnaire on Cognitive Decline in the Elderly (IQCODE) collected at baseline only.

We will also collect the following clinical measures and resource use data:

- Length of stay in each care setting within the participating NHS Trusts (acute hospital; residential rehabilitation/intermediate care facilities; hospital-at-home/early discharge services) at 1 month, 3 months and 6 months (follow-ups all ± 5 days);
- Discharge destination from index hospitalisation at 1 month, 3 months and 6 months (follow-ups all ± 5 days);
- Mortality at 1 month, 3 months and 6 months (follow-ups all ± 5 days);
- Hospital re-admissions in the participating Trust at 6 months (± 5 days);
- Hospital service use in the participating Trust (inpatient, outpatient, day case and emergency department) over 6 months prior to baseline assessment; 1 month prior to 1 month assessment, 3 months prior to 3 month and 6 month assessments (follow-ups all ± 5 days);
- 4AT at baseline and discharge from study ward (discharge ± 5 days);
- Charlson Co-morbidity Index at baseline only.

For qualitative data collection schedule for patient, suitable informant and NHS professional participants, see section 9.

For peer review only - http://bmjopen.bmj.com/site/about/guidelines.xhtml

5.7 Assessment Schedule

	Admission to study site	Enrol ment	Baseline	Post-op period		
TIMEPOINT	-T2	-T1	0	*T1 (1 month, ± 5 days)*	*T2 (3 months , ± 5 days)*	*T3 (6 months , ± 5 days)*
Eligibility screen	X					
Study information given	X					
Informed consent		X				
INTERVENTIONS:						
PERFECT-ER*	——	——	——	—— - - -	- - -	
Treatment as usual*	——	——	——	—— - - -	- - -	
ASSESSMENTS:						
MMSE (P)			X	X	X	X
DEMQOL (P)			X	X	X	X
EQ-5D-5L self-complete (P)			X	X	X	X
howRwe (P)			X	X**		
Timed Up & Go (P)					X	
BADLS (SI)			X	X	X	X
DEMQOL-Proxy (SI)			X	X	X	X
EQ-5D-5L Proxy (SI)			X	X	X	X
EQ-5D-5L Carer self-report (SI)			X	X	X	X
CSRI** (SI)			X	X	X	X
Number of days in institutional care (SI)				X	X	X
howRthey (SI)			X	X**	X	X

For peer review only

For peer review only - http://bmjopen.bmj.com/site/about/guidelines.xhtml

Patient's place of residence (SI)			X****	X	X	X
CDR (SI)			X			X
IQCODE (SI)			X			
Length of stay in index hospitalisation				X*****	X	X
Discharge destination from index hospitalisation				X	X	X
Mortality				X	X	X
Hospital re-admission rates						X
Hospital service use******			X	X	X	X
4AT			X	X**		
Charlson Co-morbidity Index			X			

*PERFECT-ER and treatment as usual continue up until discharge from study ward. Due to differences in length of stay in the study sites, T1 assessments may take place in the study site for some participants;

** Patients may be discharged from study ward before or after T1. Measure to be collected at whenever this point maybe ± 5 days;

***duration of retrospective period covered varies by assessment point;

****pre-baseline ordinary residence;

***** If patient is still in acute hospital at 30 days this will be recorded;

******from hospital patient records, of service use within site of index hospitalisation

See section 9 for details on when qualitative work will be undertaken.

5.8 Sample Size

An overall sample size of 400 (200 per arm) patient participants will be sought. This will be comprised of 40 participants in ten different centres. This sample size was not decided on the basis of efficacy analyses (i.e. this is a feasibility study) but was decided on pragmatic groups of what would appear to be achievable in the time

For peer review only - http://bmjopen.bmj.com/site/about/guidelines.xhtml

available. In addition, these data will be used to estimate the intra-class correlation coefficient for each outcome measure. Assuming a coefficient of no more than 0.1, 400 subjects from ten centres should provide a standard error of no more than 0.041.

5.9 Recruitment and Retention

5.9.1 Recruitment

Recruitment will be organised on a site specific basis. Each research site will have equivalent of a 2 day per week local PERFECTED research worker who will be responsible for obtaining informed consent and data collection. This resource will be used flexibly in each site to maximise the chances of achieving target recruitment. Where appropriate, staff from the Clinical Research Networks (CRN) will assist recruitment and other trial activities. It is anticipated that the 10 sites will each recruit 40 patients over a 10 month period (commencing circa 01.11.2016 and ending 31.08.2017, follow-up end date 28.02.2018). This equates to only needing to recruit one patient and suitable informant dyad per week. However, we reserve the right to over recruit in some sites if others are not meeting targets to ensure total and arm sample size are achieved.

The PERFECTED research workers will collaborate with relevant clinical staff (including but not exclusively the study ward Trauma Co-ordinators (TCs) and key Emergency Department colleagues) to identify all new admissions and screen for pre-recruitment eligibility. Participants (patient and where possible their potential suitable informant) will be approached and given information about the study as soon as clinically appropriate. Participants will be given up to 5 days after their operation to consider the information. Once the participant deems they have had enough time to review the information (before the 5 day post-op time period elapses) their consent decision will be sought. The process of recruitment will be tightly coordinated and monitored weekly to identify trends that might give rise to differential recruitment, and to enable corrective action.

5.9.2 Retention

Patient participants will be followed up unless they withdraw. Depending upon the circumstance, this may involve visits taking place in the patient participants' usual place of residence. Because of the nature of the patient population and the foreseen different relationships between patient participant and the suitable informants, the

For peer review only - http://bmjopen.bmj.com/site/about/guidelines.xhtml

PERFECTED research worker will aim to provide flexible ways for both parties to remain within the study. This may involve: at each visit agreeing the date of the next appointment, agreement for telephone calls to be made to either patient participant or suitable informant participant (dependent upon circumstances) prior to visits. Where face to face visits are not possible, telephone calls and/or postal mechanisms may be arranged with suitable informant (where appropriate) participants in order to collect data.

5.10 Assignment of Intervention

5.10.1 Allocation

As the trial is an open trial using a cluster-randomised controlled design, the unit of analysis is the cluster (hospital site). Across the five regions (East Anglia, East Midlands, London, Scotland and Yorkshire) the participation of 10 hospitals based on the aforementioned site eligibility criteria has been secured. Hospitals will be randomised to active or control arm within geographical region using a simple randomisation process. An ad hoc programme will be written in SAS to carry out this procedure.

5.10.2 Blinding

The nature of PERFECT-ER intervention means that it cannot be delivered blind. Patient and suitable informants may know which arm they are allocated to, as will the NHS professionals working in the hospital context. Analyses will be carried out by the trial statistician blinded to trial arm and cluster.

5.11 Data Collection, Management and Analysis

5.11.1 Data Collection Methods

Data will be collected at the time points indicated on the participant assessment schedule. The PERFECTED research workers will complete paper Case Report Forms (CRF) when collecting data from participants, suitable informants and patient's relevant medical records. They will enter data onto a central database via an online system once they have Internet access. PERFECTED research workers will receive training on data collection and use of the online system. Identification logs, screening logs and enrolment logs will be kept locally, either in paper or electronic form.

Source data worksheets will be drafted by NCTU data manager with the CI, trial statistician, programme manager, trial manager and relevant PIs. These will be piloted

For peer review only - http://bmjopen.bmj.com/site/about/guidelines.xhtml

and finalised. The database specification will be prepared by NCTU data manager and approved by the CI, trial statistician, programme manager and trial manager prior to the database being built. The database will be prepared by NCTU data programmer and tested by the trial statistician and study sites staff for user accessibility prior to the final system being launched.

Data collection, data entry and queries raised by a member of the PERFECTED trial team will be conducted in line with NCTU and trial specific data management working practices.

Clinical trial team members will receive protocol training. All data will be handled in accordance with the Data Protection Act 1998.

5.11.2 Data management

Within each trial site patient participants will be allocated a unique trial participant identification number (PIN). This will include a reference to the centre but not the trial arm. Suitable informants will be given a SI-PIN. This will enable the study to link the suitable informant's contribution to the patient participant(s) and for this to be uniquely coded.

Data will be entered under this PIN (or SI-PIN) onto the central database stored on the server based at UEA. The database will be password protected and only accessible to members of the PERFECTED trial team, the participating sites and external regulators. The server is in a secure room, which is protected by CCTV, where access is restricted to members of the UEA information systems team by security door access. The study database will be built using Microsoft SQL Server tools and direct access will be restricted to NCTU data management staff. Data entry will be via webpages created using Microsoft.NET technology. All Internet traffic will be encrypted using the standard secure sockets layer (SSL) methodology. The data entry system will validate data on entry to ensure it is of the expected type and range of values. Periodically, and at database lock the data will be further validated for errors and inconsistencies. The database is linked to an audit tool where all data additions, modifications and deletions are recorded with time/date and the details of the person making the change via individual user identification credentials. The database is designed to comply with the ICH guidance for Good Clinical Practice (GCP) within the working practices for data management in NCTU and, where appropriate, with UEA IT procedures.

For peer review only - http://bmjopen.bmj.com/site/about/guidelines.xhtml

The database and coding values have been developed by the NCTU data manager in conjunction with the CI, study statistician, programme manager, other NCTU members and the trial team. The database software provides a number of features to help maintain data quality, including: maintaining an audit trial, allowing custom validations on all data, allowing users to raise data query requests, and search facilities to identify validation failure/missing data. Further details can be found in the PERFECTED Trial Data Management Plan. Following completion of the trial, the database will be retained on the UEA servers for 10 years.

5.11.2.1 Physical storage of information

Information collected during the course of the research will be stored locally and, as appropriate, at the University of East Anglia in line with the Data Protection Act (1998) and GCP requirements.

The identification, screening and enrolment logs, linking participant identifiable data to the PIN and SI-PIN, will be held locally by the research sites and potentially at NCTU. This will either be held in written form in a locked filing cabinet or electronically in a password protected form on hospital computers. After completion of the trial the identification, screening and enrolment logs will be stored securely by the sites for a minimum of 10 years.

To ensure the study is running appropriately a selection of consent forms will be sent to the University of East Anglia from local sites via secure fax. These will be stored at University of East Anglia in a locked filing cabinet. Participant consent will be sought to enable this monitoring. These will be stored securely for a minimum of 10 years after the study has finished before being securely destroyed.

5.11.3 Non-Adherence and Non-Retention

The relevant consent forms will explain that if a participant wishes to withdraw from the study, data acquired prior to that point will be retained. Reason for withdrawal, if given, will be recorded. Patient participants will not be replaced; in the case where the suitable informant is no longer available to participate, replacement suitable informants will be sought where possible to maintain patient participant involvement. In cases during follow-up where a suitable informant replacement cannot be found, patient participant's involvement in the study will continue where possible.

For peer review only - http://bmjopen.bmj.com/site/about/guidelines.xhtml

5.11.4 Statistical Methods

As a feasibility, the main aim is to assess feasibility of procedures and provide information for a definitive trial. Nonetheless, multi-level modelling (employed to account for the hierarchical nature of the data) with a Normal error term will be used to provide initial estimates of between group differences (with 95% confidence intervals). These models will include prognostic baseline variables, stated in the statistical analysis plan prior to any data being analysed. We will provide estimates (with 95% confidence intervals) of the intra-class correlation coefficients for all efficacy endpoints to inform future sample size calculations. We will record recruitment and retention rates, report data completeness (and patterns of missing data, e.g. probability of being missing conditional on baseline characteristics) for all measures. The economic evaluators will carry out the analysis of the cost and outcomes data collected. Quantitative measures of fidelity will be analysed descriptively to indicate the degree to which the intervention has been adhered to across different sites.

5.11.4.1 Statistical Analysis Plan

A full statistical analysis plan (SAP) will be developed between the trial statistician and Chief Investigator and agreed with the trials governance committees. The final approved version of the SAP will be kept in the TMF and eTMF.

5.11.5 Economic evaluations

Hospital care data will be collected for 400 patients retrospectively from the hospital records of the participating NHS Trusts, and data on health and other service use by patients and suitable informants will be collected at each of the informant interviews, allowing comparison of both sources for some items, and assessment of feasibility of collecting data on carers inputs (e.g. hours of care provided). The CSRI will be adapted to fit the needs of this study. Unit costs will come from national reference costs and tariffs, the Personal Social Services Research Unit Compendium, or calculated anew if necessary; costs associated with the intervention will be collected in a consistent manner using local information from participating hospitals. We will investigate the feasibility of extracting both hospital activity and costs data electronically with individual sites; however we plan to include an extraction pro-forma in the CRF to allow data to be collected in a consistent fashion across sites if electronic extraction proves impractical. Hospital service use data collected from medical records of the participating NHS Trusts will be used to validate the information provided in the CSRI,

For peer review only - http://bmjopen.bmj.com/site/about/guidelines.xhtml

and if necessary to “fill in” missing hospital service use information. In the case of conflicts between the administrative data extracted from medical records and self-reported data, we will use the former. In addition we will explore methods of measuring the association of hospital costs with inputs, quality and needs. Informal care inputs will be valued using replacement wage and opportunity cost approaches, and comparing them in sensitivity analyses.

Cost-effectiveness analyses will be from health and social-care and societal perspectives, over 0-6 months, to generate preliminary findings. We will explore the following outcomes: (1) BADLS (2) QALY gain for patients, computed from DEMQOL-proxy completed by carers, with societal weights (new QALY-generating algorithm from Banerjee et al, 2012); QALY gain for patients computed from EQ-5D-proxy completed by suitable informants, with societal weights; QALY gain for carers computed from EQ-5D, with societal weights. In each case, incremental cost-effectiveness ratios will be computed as needed, and cost-effectiveness acceptability curves generated in the standard way. Net-benefit regressions will make it possible to control for centre, baseline outcome measures (where appropriate) and baseline costs. Multi-level analyses appropriate for the clustered-randomised trial design will allow comparison of instrument scores, comparison of societal, patient and carer weights for QALYs generated from DEMQOL-proxy, and comparison of different approaches to valuing informal care.

Findings will also be used to explore the feasibility of economic data collection. We will examine the data collected via the CSRI (1) for completeness/ missingness and (2) to compare the levels of agreement between CSRI hospital service use items with those collected from routine reporting sources. We will measure the researcher time required to extract routinely reported hospital data; we will also measure the researcher and participant time required to collect participant-reported data. We will interrogate the data to test for differences between participating hospitals to inform later generalizability. We will draw upon these results to make recommendations for cost-collection methods for the definitive trial, taking into account research costs, data completeness and reporting burden experienced by participants.

For peer review only - http://bmjopen.bmj.com/site/about/guidelines.xhtml

5.11.6 Additional Analyses – Subgroup

No subgroup analyses are planned. Should it be decided, in the light of new information, during the course of the trial that a subgroup analysis is appropriate, this will be recorded in the statistical analysis plan prior to any data analyses.

5.11.7 Additional Analyses – Adjusted

See above.

5.11.8 Analysis Population and Missing Data

Analyses will be on an Intention-to-Treat basis. There are no plans for imputing missing data and thus analyses will be on a complete-case basis only.

5.12 Data Monitoring

5.12.1 Data Monitoring and Ethics Committee (DMEC)

Further details of the roles and responsibilities of the Data Monitoring and Ethics Committee (DMEC), including membership, relationships with other committees, decision making processes, and the timing and frequency of interim analyses (and description of any stopping rules and/or guidelines where applicable) are described in detail in the PERFECTED DMEC Terms of Reference (ToR).

5.12.2 Interim analyses

There will be no formal interim analysis. Analysis of recruitment rates, patient participant (including mortality) and suitable informant withdraw rates will be reviewed monthly by the Trial Management Group. These monthly meetings will also be presented with difficulties identified with delivering the intervention and collecting outcome data.

5.12.3 Data monitoring for harm

The intervention in this trial comprises a multicomponent service improvement intervention PERFECT-ER implemented at ward level. No adverse events attributable to the intervention are anticipated, however, any untoward outcomes that are noted will be reported to the CI/Site PI immediately and where appropriate escalated to senior ward staff so that ward staff can comply with their own Trust complaints or clinical incident reporting system(s) if required. Adverse events may be identified through complaints to the research team or ward manager. Adverse events will be identified and managed using normal complaints and NHS incident reporting procedures for patients and wards respectively. Any reportable incident, complaint or

For peer review only - http://bmjopen.bmj.com/site/about/guidelines.xhtml

safeguarding reporting observed will be reported to the CI/Site PI immediately and where appropriate escalated to senior ward staff so that ward staff can comply with their own trust complaints or clinical incident reporting system(s) if required. Adverse incidents, complaint or safeguarding reporting will be captured in the study report but will not be attributed to a named Trust or ward with identifiable data removed.

For peer review only

For peer review only - http://bmjopen.bmj.com/site/about/guidelines.xhtml

6 Safety reporting procedures

6.1 PERFECTED Operationalised Definitions

6.1.1 Adverse events (non-reportable)

Non-serious adverse events will not be reported because the intervention is low risk, and at a ward level. The participant-facing research procedures are also extremely low risk to participants. However if it became apparent that a patent participant was potentially suffering from depression their General Practitioner (GP) would be informed via a letter sent by the local site. Upon consenting to participate in the trial patient participants agree to this eventuality.

6.1.2 Expected serious adverse events – standard reporting

The focus of this study is to assess a service improvement intervention delivered by ward staff with the aim of improving care delivery for patients with hip fracture and confusion. This patient population has a high of incidence of ill health and co-morbidity. Thus, in this patient population, acute illness resulting in hospitalisation, new medical problems and deterioration of existing medical problems are expected. In recognition of this, events fulfilling the definition of a serious adverse event will not be reportable in this study.

Deaths, falls, pressure ulcers, and safeguarding incidents in this patient population are expected. These rates will be collected at a ward level throughout the study period. These events will not be subject to expedited reporting to the Research Ethics Committee (REC) but will be reported annually to the REC (in the annual progress report) and will also be reviewed by the relevant trial oversight committees.

For peer review only - http://bmjopen.bmj.com/site/about/guidelines.xhtml

7 Quality assurance and control

7.1 Risk Assessment

The Quality Assurance (QA) and Quality Control (QC) considerations for the trial are based on the standard NCTU Quality Management Policy that includes a formal Risk Assessment, and that acknowledges the risks associated with the conduct of the trial and proposals of how to mitigate them through appropriate QA and QC processes. Risks are defined in terms of their impact on: the rights and safety of participants; project concept including trial design, reliability of results and institutional risk; project management; and other considerations.

QA is defined as all the planned and systematic actions established to ensure the trial is performed and data generated, documented and/or recorded and reported in compliance with the principles of GCP and applicable regulatory requirements. QC is defined as the operational techniques and activities performed within the QA system to verify that the requirements for quality of the trial related activities are fulfilled.

7.2 Central Monitoring at NCTU

NCTU staff will review data for errors and missing key data points. The trial database will also be programmed to generate reports on errors and error rates. Essential trial issues, events and outputs, including defined key data points, will be detailed in the trial Data Management Plan.

7.3 On-site Monitoring

The frequency, type and intensity of routine and triggered on-site monitoring will be detailed in the Quality Management and Monitoring Plan (QMMP). The QMMP will also detail the procedures for review and sign-off of monitoring reports.

7.3.1 Direct access to participant records

Participating investigators must agree to allow trial related monitoring, including audits, REC review and regulatory inspections, by providing access to source data and other trial related documentation as required. Participant consent for this must be obtained as part of the informed consent process for the trial.

7.4 Trial oversight

Trial oversight is intended to preserve the integrity of the trial by independently verifying a variety of processes and prompting corrective action where necessary. The

For peer review only - http://bmjopen.bmj.com/site/about/guidelines.xhtml

processes reviewed relate to participant enrolment, consent, eligibility, and allocation to trial groups; adherence to trial interventions and policies to protect participants, including reporting of harms; completeness, accuracy and timeliness of data collection; and will verify adherence to applicable policies detailed in the Compliance section of the protocol. Independent trial oversight complies with NCTU trial oversight policy.

In multi-centre trials this oversight is considered and described both overall and for each recruiting centre by exploring the trial dataset or performing site visits as described in the PERFECTED Quality Management and Monitoring Plan.

7.4.1 Trial Management Team

The Trial Management Team (TMT) will be set up to assist with developing the design, co-ordination and day to day operational issues in the management of the trial, including budget management. The membership, frequency of meetings, activity (including trial conduct and data review) and authority will be agreed with NCTU and Chief Investigator.

7.4.2 Trial Management Group

A Trial Management Group (TMG) has been set up to assist with developing the design, co-ordination and strategic management of the trial. The membership, frequency of meetings, activity (including trial conduct and data review) and authority will be covered in the TMG terms of reference.

7.4.3 Programme Steering Committee

The Independent Programme Steering Committee (PSC) is the independent group responsible for oversight of the trial in order to safeguard the interests of trial participants. The PSC provides advice to the CI, NCTU, the funder and sponsor on all aspects of the trial through its independent Chair. The membership, frequency of meetings, activity (including trial conduct and data review) and authority will be covered in the PSC terms of reference.

7.4.4 Independent Data Monitoring Committee

The independent Data Monitoring and Ethics Committee (DMEC) is responsible for safeguarding the interests of trial participants, monitoring the accumulating data and making recommendations to the PSC on whether the trial should continue as planned. The membership, frequency of meetings, activity (including review of trial conduct and

For peer review only - http://bmjopen.bmj.com/site/about/guidelines.xhtml

data) and authority will be covered in the DMEC terms of reference. The DMEC will consider data in accordance with the statistical analysis plan and will advise the PSC through its Chair.

7.4.5 Trial Sponsor

The role of the sponsor is to take responsibility for securing the arrangements to initiate, manage and finance the trial. University of East Anglia (UEA) is the trial sponsor and has delegated the duties as sponsor to Chief Investigator Professor Chris Fox and NCTU.

For peer review only

For peer review only - http://bmjopen.bmj.com/site/about/guidelines.xhtml

8. Ethics and Dissemination

8.1 Research Ethics Approval

Before initiation of the trial at any clinical site, the protocol, all informed consent forms and any material to be given to the prospective participant will be submitted to the relevant REC for approval. Any subsequent amendments to these documents will be submitted for further approval.

The rights of the participant to not participate in the trial without giving a reason must be respected. After the participant has entered the trial, they should remain within the trial for the purpose of follow up and data analysis according to the service delivery option to which they have been allocated. However, the participant remains free to change their mind at any time and withdraw their consent to take part in the research trial including follow-ups, without giving a reason and without prejudicing their further treatment.

8.2 Competent Authority Approvals

This is not a Clinical Trial of an Investigational Medicinal Product (IMP) as defined by the EU Directive 2001/20/EC, therefore, a Competent Authority Approval is not required.

8.3 Other Approvals

The protocol will be submitted by those delegated to do so to the HRA and relevant other departments for approval as required in each country. A copy of the Participant Information Sheets (PIS), consent forms and other relevant annexes will be entered onto local headed paper and must be forwarded to the co-ordinating centre before participants are recruited to the trial.

The protocol has received formal approval and methodological, statistical, clinical and operational input from the NCTU Protocol Review Committee. The document has also benefited from review by members of PERFECTED Data Monitoring and Ethics Committee, Programme Steering Committee, Programme Advisory Group and Service User Advisory Groups. Each of these groups has embedded Public Patient Involvement (PPI) members, with the latter group consisting solely of PPI members.

For peer review only - http://bmjopen.bmj.com/site/about/guidelines.xhtml

8.4. Protocol Amendments

Substantial protocol amendments (e.g. changes to eligibility criteria and outcomes), will be decided by the Chief Investigator, and guided by the Trial Management Group. Each site Principal Investigator will be informed of the potential changes. Amendments will be submitted to the HRA for approval. Once approved, protocol amendments will be circulated to trial personnel

8.5 Declaration of Interests

The investigators named on the protocol have no financial or other competing interests that impact on their responsibilities towards the scientific value or potential publishing activities associated with the trial.

8.6. Confidentiality

Any paper copies of personal trial data will be kept at participating sites in a secure location with restricted access. Only non-identifiable data will be kept at the PERFECTED office with authorised PERFECTED trial team and NCTU staff members having access. Only staff working on the trial will have password access to this information.

Confidentiality of patients' personal data is ensured by not collecting patient names on CRFs that will be sent to NCTU and storing the data in a pseudonymised fashion at NCTU. At enrolment both patient and suitable informant participants will be issued a Participant Identification Number (PIN) or Suitable Informant Participant Identification Number (SI-PIN) and this will be the primary identifier for the participants, with secondary identifiers of month and year of birth and initials.

The patients' and suitable informants' consent forms will carry their name and signature. These will be kept at the trial site, and a copy sent to NCTU for monitoring purposes. They will not be kept with any additional participant data.

8.7 Indemnity

UEA holds insurance to cover participants for injury caused by their participation in the clinical trial. Participants may be able to claim compensation if they can prove that UEA has been negligent. However, as this clinical trial is being carried out in a hospital, the hospital continues to have a duty of care to the participant in the clinical trial.

For peer review only - http://bmjopen.bmj.com/site/about/guidelines.xhtml

UEA does not accept liability for any breach in the hospital's duty of care, or any negligence on the part of hospital employees. This applies whether the hospital is an NHS Trust or not. This does not affect the participant's right to seek compensation via the non-negligence route.

Participants may also be able to claim compensation for injury caused by participation in this clinical trial without the need to prove negligence on the part of UEA or another party. Participants who sustain injury and wish to make a claim for compensation should do so in writing in the first instance to the Chief Investigator, who will pass the claim to UEA's insurers, via the Sponsor's office.

Hospitals selected to participate in this clinical trial shall provide clinical negligence insurance cover for harm caused by their employees and a copy of the relevant insurance policy or summary shall be provided to UEA, upon request.

8.8 Finance

The trial is fully funded by the National Institute for Health Research (NIHR). The trial represents a discrete yet integral part of the wider Peri-operative Enhanced Recovery hip FracturE Care of paTiEnts with Dementia "PERFECTED" Programme Grant for Applied Research, funder reference DTC-RP-PG-0311-12004.

8.9 Archiving

The investigators agree to archive and/or arrange for secure storage of PERFECTED trial materials and records for a minimum of 10 years after the close of the trial unless otherwise advised by the UEA.

8.10 Access to data and data sharing

Patients and Suitable Informants will consent to permit the sharing of their research data with researchers from external organisations. Requests for access to this data will be considered, and approved in writing, where appropriate, after formal application to the TMG and PSC. Considerations for approving access are documented in the TMG and PSC terms of reference. The CI and trial statistician at UEA will have access to the full trial dataset.

For peer review only - http://bmjopen.bmj.com/site/about/guidelines.xhtml

8.11 Publication policy

8.11.1 Trial results

The results of the trial will be disseminated. Ownership of the data arising from the study resides with the trial team. The publication policy will be in line with the rules of the International Committee of Medical Journal Editors. The TMG will decide on authorship with any difficulties being resolved by the PSC.

8.11.2 Authorship

The TMG will nominate a writing group, which will consist of members of the TMG and will be responsible for drafting the manuscript publication. These individuals will be named on the final publication.

8.11.3 Reproducible research

The trial protocol will be published and made available for public access throughout the trial period.

For peer review only

For peer review only - http://bmjopen.bmj.com/site/about/guidelines.xhtml

9. Ancillary Studies

An additional component of the trial is the qualitative exploration of stakeholder views on delivery of the PERFECT-ER intervention. This will be undertaken by PERFECTED study team members. Process evaluations are common-place in complex intervention trials such as this. In this context, stakeholders will be patients and suitable informants who will have experienced service delivery via PERFECT-ER and NHS professionals experiencing and implementing PERFECT-ER. This qualitative study will only take place at the five active sites implementing PERFECT-ER. This information will be essential for the development of the future definitive trial.

The views of frontline staff (NHS professionals) and hospital administrators/finance officers in each active site will be explored to illustrate how time is used to provide appropriate care to people who are confused (including but not specifically those with dementia) and have hip fracture, and to what extent the ward/centre's administrative system tracks time spent with these patients. This will allow us to investigate the feasibility of directly measuring the amount of staff time dedicated to care for individual trial participants. This work will be carried out in collaboration with the UEA qualitative research team. To be clear, as is common in qualitative research approaches, direct quotes from participants (patients, suitable informants and NHS Professionals) will be used in publications. To ensure confidentialities and anonymity identifying features such as names of people, hospitals and places will be replaced using pseudonyms. The transcription of these qualitative research encounters will take place internally.

9.1 Patient and suitable informant; recruitment, data collection, consent and withdrawal for qualitative study

Upon consenting to the clinical trial, patients and suitable informants will have agreed to the possibility of being approached to take part in a research interview (face to face with patients with telephone interviews offered as an additional option to suitable informants). Interviews will be undertaken by qualitatively trained researchers based at the UEA. This interview will take place once the patient has been discharged from the trial hospital. Information regarding the research interview study will be made available to the selected patient and suitable informant participants during the 30 day follow-up data collection activities. At this point in time a pragmatic assessment of the patient participant's capacity to contribute to research interview will be made. It is important to note that a patient participant who is assessed as lacking capacity to

For peer review only - http://bmjopen.bmj.com/site/about/guidelines.xhtml

consent may still be able to be supported to participant in a research interview. Patients who clearly lack capacity to engage in a research interview about recent acute care experiences, for example patients who are chronically confused will not be approached. These assessments will be made by the experienced research nurses and, where appropriate, in consultation with the patient's suitable informant and/or legal representative/consultee. This approach is informed by earlier PERFECTED Work Packages.

The interviews will ask for the patient and suitable informant's experiences of their hip fracture service delivery and their experience of the research study. Up to 5 patients and 5 suitable informants from each site implementing PERFECT-ER will be recruited. It is expected these interviews will highlight strengths, weaknesses and ways to adapt PERFECT-ER and the study mechanisms from a service-user perspective to inform the definitive trial bid.

For interviews (either face to face or telephone) with suitable informants, it is anticipated that the qualitatively trained researcher (based at the UEA) may be accompanied by a trained lay researcher (PPI member) who will assist in the interview, bringing their unique service-user perspective to the process. This method of PPI involvement was successfully integrated in previous component studies of the PERFECTED research programme.

In line with local legislative frameworks the mental capacity of patient participant interviewees will be continually assessed. Where and when appropriate, techniques will be deployed to facilitate involvement by the researcher undertaking the interview.

Relevant documents will remind patient and suitable informant participants of the right to withdraw.

9.1.1. Inclusion and exclusion criteria for patient participants in qualitative study

Inclusion criteria:

1) Patient must be part of the PERFECT-ER trial.

Exclusion criteria:

2) Patient assessed not to have capacity to be able take part in a research interview.

For peer review only - http://bmjopen.bmj.com/site/about/guidelines.xhtml

9.1.2 Inclusion and exclusion criteria for suitable informants in qualitative study

Inclusion criteria:

1) Suitable informant must be part of the PERFECT-ER trial.

Exclusion criteria:

1) Not applicable

9.2 Supporting Public Patient Involvement (PPI) members in research activities

PPI was not only a significant feature in the creation and development of the whole PERFECTED programme but also PPI is a central theme in all the study's research activities. Prof Poland (UEA) is a national expert on public involvement in health research and is the lead for all PPI activities within the study. Prof Clive Ballard is also a member of the PERFECTED team, he is Professor of Age-Related Diseases at Kings College London and also a recognised expert on PPI. In addition to assisting the PERFECTED team in creating ethical proposals, public/patient information sheets and dissemination activities. Using learning gained from earlier phases of PERFECTED, notably the observations undertaken by PPI members as part of WP1 phase 3 (MEC N 14/EM/1020), carer interviews undertaken as part of WP1 P4 (MEC N 15/EE/0007) and advisor roles as part of WP2 (MEC N 15/SC/0294) PPI members will be trained in interview and listening skills. The majority of these PPI members will have their training from previous PERFECTED sub-studies mentioned above 'refreshed'. Any new PPI members will undertake the bespoke training which will cover; *what are research ethics?; the ethics of a research interview; role boundaries and confidentiality; safeguarding; asking the right questions and active listening; reflections and analysis*.

To enable payment and ensure indemnity insurance, PPI members have temporary contracts with the University of East Anglia (UEA), including providing proof of right to work within the United Kingdom. Recruited PPI members will be receive approximately £11.03 per hour. PPI members are also able to claim all reasonable expenses for travel in connection with this role. Due to the nature of the role there will be the need to do some preparatory work outside these hours which the research team will support but not provide payment for. The paid nature and demands of this role are very unusual in PPI involvement in research. Advice has been sought from Profs Poland and

For peer review only - http://bmjopen.bmj.com/site/about/guidelines.xhtml

Ballard, Matt Murray (Research Engagement Manager, Alzheimer's Society) and members of UEA's Human Resources Department. There is clear potential for issues in relation to tax and benefits for PPI members. These will be handled sensitively in partnership with the UEA's Human Resources Department, the PPI member's parent PPI organisation, the PPI members themselves, the local site and Dr Simon P Hammond (Programme Manager and Research Fellow). As all of these members have not yet been recruited it is unfeasible to cover every possible aspect of any potential financial issue in this protocol.

Dr Tamara Backhouse will act as PPI liaison for the UEA team. The PERFECTED team have experience of facilitating such involvement within and beyond this particular research programme.

9.2.1Potential issues associated with PPI members undertaking research activities

Below are a number of issues in relation to supporting the involvement of PPI members in undertaking research activities and how these will be in mitigated by the project and processes embedded within it.

A) Confidentiality: PPI interviewers will be aware that disclosure of any data outside the parameters of the project is considered a serious breach of contract and will be subject to disciplinary and possibly legal proceedings. Confidentiality issues will be discussed with the PPI members by the UEA study team prior to their involvement. Across each of the previous four sub studies we have never had an issue with PPI members making serious disclosures.

B) Providing adequate support for PPI members: Comprehensive guidelines regarding public involvement in research by INVOLVE (www.invo.org.uk), inform the practices within this study. PPI members will receive suitable instruction from UEA researchers regarding their expected role in the study. The primary source of emotional support will come from within the core research team. Dr Backhouse is a care-home worker for 20 years and her doctorate was based in such an environment. Hence she has significant experience of dementia associated behaviours. Dr Hammond, who will support Dr Backhouse, is a Psychologist who has over 15 years' experience in working with vulnerable populations in deeply difficult situations.

For peer review only - http://bmjopen.bmj.com/site/about/guidelines.xhtml

C) Specific competencies of PPI members: As with all researchers, regardless of ‘lay’ or ‘expert’ labels, there is a need to ensure individuals are competent in relation to the undertaking of the specific tasks. The existing working relationships with PPI members generated from previous PERFECTED inputs mean that the UEA team are highly confident that the PPI members in question at all three sites are fully competent to carry out the duties required in this study.

D) Respect and sensitivity of PPI members: It will be made clear at various points within the study that the PPI advisors bring a unique perspective to the research environment, which is both valid and respected. This ethos transcends the whole of the PERFECTED research programme, and this study will be no different. Participating PPI members have the contact number of UEA researchers, should they feel the need in the subsequent days to talk over any issues.

For peer review only

For peer review only - http://bmjopen.bmj.com/site/about/guidelines.xhtml

9.3. Patient and Suitable Informant recruitment diagram

Patient and “suitable informant” entered as a dyad into the trial and consented to be approached about the qualitative study

During trial Time 1 (1 month post-op, ± 5 days) a local PERFECTED research worker (assess eligibility and if appropriate) provides relevant Participant Information Sheet for qualitative study, explaining purpose and what is required of them for the qualitative study.

Participants decide/declines to take part with decision recorded by Local PERFECTED research worker. For those assessed as not having mental capacity but having capacity to participate in a research interview, local PERFECTED research worker liaises with legal representative (Scotland) or personal/nominal consultee and enacts according to local legislative framework.

Participants’ contact details and preferences passed to UEA qualitative research team who contact potential participant or their gatekeeper (in case of patients not living at home) and arrange a convenient time to carry out the research interview.

UEA qualitative research team member makes contact, answers any questions, seeks consent and undertakes research interview.

9.4 NHS professionals: recruitment, data collection, consent and withdrawal

The views of those NHS professionals experiencing, implementing and using PERFECT-ER will also be sought via qualitative methods. Following implementing the PERFECT-ER intervention, the views of NHS professionals from the five active arm hospitals will be sought. A pragmatic multimethod approach, recruiting a combination of focus groups and interviews (including telephone interviews, as appropriate), will be used to elicit perspectives from a broad range of ward staff, including those championing, implementing and using the PERFECT-ER intervention. The focus groups, interviews and telephone interviews will be conducted by qualitatively trained

For peer review only - http://bmjopen.bmj.com/site/about/guidelines.xhtml

researchers (based at the UEA). NHS professionals will be given relevant study documents and as much time as they need to consent or decline. Relevant documents will remind NHS professional participants of their right to withdraw. Again these qualitative methods will seek to highlight strengths, weaknesses and ways to adapt PERFECT-ER and the study mechanisms from a stakeholder perspective to inform the definitive trial bid.

9.4.1 Inclusion and exclusion criteria for NHS professionals in qualitative study

Inclusion criteria:

1) Must be a NHS professional involved in implementing and/or using PERFECT-ER.

Exclusion criteria:

1) None.

9.4.2 NHS professional recruitment diagram

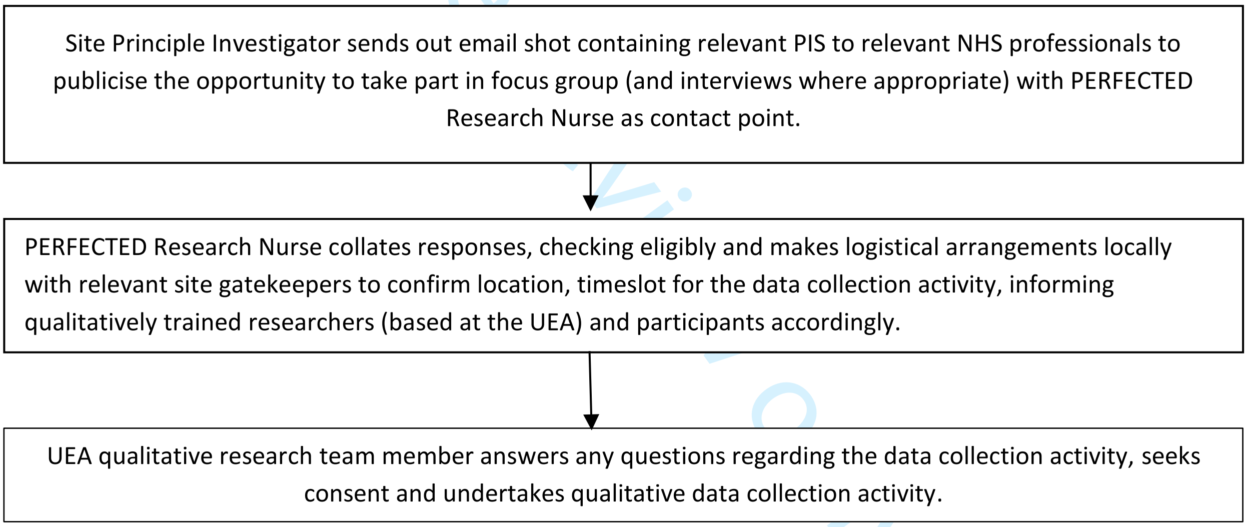

For peer review only - http://bmjopen.bmj.com/site/about/guidelines.xhtml

9.5 Qualitative procedure schedule

	Baseline	Post-op period				
TIMEPOINT	0	*T1 (1 month, ± 5 days)*	*T1Q (mutually convenient time prior to T2)*	*T2 (3 months, ± 5 days)*	*T3 (6 months, ± 5 days*	*T4 (post recruitment window closing circa Sept-Oct 2017)*
Study information given	Not applicable to qualitative study	**X**		Not applicable to qualitative study		
Informed consent			**X**			
ACTIVITY						
Patient interviews			**X**			
Suitable Informant interviews			**X**			
NHS Professional interviews or focus groups*						**X****

*A pragmatic multimethod approach combining focus groups and interviews will be used to elicit views from NHS professionals. NHS professionals will not take part in more than one qualitative data collection exercise.

**Relevant study documents will be given to NHS professionals and as much time as they need to consent or decline the invitation to take part in the qualitative study.

9. Qualitative data storage and transcription

Qualitative data will be collected by PERFECTED study team members based at the University of East Anglia (UEA). Upon completing the qualitative research encounter the audio file created will be securely physically transferred back to the University of East Anglia. On returning to UEA the qualitative researcher will transfer the audio file to the secure study database and delete the audio file from the Dictaphone. This audio file will be transcribed internally at UEA with identifying features removed as per usual procedures for qualitative research methods. This data will be kept for a minimum of 10 years after the study ends and then securely destroyed.

For peer review only

For peer review only - http://bmjopen.bmj.com/site/about/guidelines.xhtml

10. Protocol Amendments

For peer review only

For peer review only - http://bmjopen.bmj.com/site/about/guidelines.xhtml

11. References

Adults with Incapacity (Scotland) Act 2000 http://www.legislation.gov.uk/asp/2000/4/contents

Alzheimer's Society (2009) "counting the costs: caring for people with dementia on hospital wards" http://www.alzheimers.org.uk/site/scripts/download_info.php?downloadI

Chan AW, Tetzlaff JM, Altman DG et Al. SPIRIT 2013 Statement: Defining Protocol Items for Clinical Trials. *Ann Intern Med* 2013; 158:200-207.

Chan AW, Tetzlaff, Gotzsche et Al. SPIRIT 2013 explanation and elaboration: guidance for protocols of clinical trials. *BMJ* 2013; 346: e7586.

Cooper C, Campion G, and Melton LJ, (1992) Hip fractures in the elderly: a world-wide projection. Osteoporos Int; 2, 285.

Department of Health (2005). Mental Capacity Act (England & Wales). London, HMSO. http://www.legislation.gov.uk/ukpga/2005/9/contents

Department of Health (2013a) Enhanced recovery care pathway A better journey for patients seven days a week and better deal for the NHS -: http://www.nhsiq.nhs.uk/resource-search/publications/enhanced-recovery-care-pathway-review.aspx#sthash.EBWmvDCu.dpuf

Gruneir A, Bell CM, Bronskill SE et al. (2010) "Frequency and patterns of emergency department visits by long-term care residents-a population-based study". J Am Geriatri Soc; 58, 510-517.

Gullberg B, Johnell O, and Kanis JA. (1997) "Worldwide projections for hip fracture". Osteoporos Int; 7; 407-413.

For peer review only - http://bmjopen.bmj.com/site/about/guidelines.xhtml

Hernlund E, Svedbom A, Ivergård M, Compston J, Cooper C, Stenmark J, McCloskey EV, Jönsson B, and Kanis JA. (2013) "Osteoporosis in the European Union: medical management, epidemiology and economic burden" : A report prepared in collaboration with the International Osteoporosis Foundation (IOF) and the European Federation of Pharmaceutical Industry Associations (EFPIA). Arch Osteoporos. Dec;8(1-2):136.

McGilton, K.S, Davis, A.M., Nagile, G., Mahomed, N., Flannery, J., Jaglai, S. and Stewart, S. (2013) "Evaluation of patient-centred rehabilitation model targeting older persons with a hip fracture, including those with cognitive impairment". BMC Geriatrics, 13, 136-144.

Mulhern B, Rowen D, Brazier J, Smith S, Romeo R, Tait R, *et al.* Development of DEMQOL-U and DEMQOL-PROXY-U: generation of preference-based indices from DEMQOL and DEMQOL-PROXY for use in economic evaluation. *Health Technol Assess* 2013;17 (5)

National Institute of Health (2014) GCP Resources. http://www.crn.nihr.ac.uk/workforce_development/learning_and_development/gcp/gcp_resource/standards_in_practice/safety_reporting/non_ctimps

NHS Service Improvement model (Plan-Do-Study-Act) © Copyright Institute for Innovation NHS Institute for Innovation and Improvement 2006-2013 http://www.institute.nhs.uk/quality_and_service_improvement_tools/quality_and_service_improvement_tools/plan_do_study_act.html

Odén A, McCloskey EV, Johansson H. and Kanis JA. (2013) "Assessing the impact of osteoporosis on the burden of hip fractures". Calcif Tissue Int. Jan;92(1):42-9. doi: 10.1007/s00223-012-9666-6.

Royal College of Psychiatrists (2009) National Audit of Dementia Project www.rcpsych.ac.uk/clinicalservicestandards/centreforqualityimprovement/nationalauditofdementia.aspx

For peer review only - http://bmjopen.bmj.com/site/about/guidelines.xhtml

Seitz DP, Adunuri N, Gill SS, and Rochon PA. (2011) "Prevalance of dementia and cognitive impairment among older adults with hip fractures". J Am Med Dir Assoc; 12: 556-564.

Smith TO, Hameed Y, Cross J, Sahota O, and Fox C.(2013) "Assessment of people following hip fracture with cognitive impairment: a systematic review and meta-analysis". Archives of Gerontology and Geriatrics 57, 117–126

UK Government (1998) The Data Protection Act http://www.legislation.gov.uk/ukpga/1998/29/contents

For peer review only - http://bmjopen.bmj.com/site/about/guidelines.xhtml

BMJ Open

PERFECTED enhanced recovery pathway (PERFECT-ER) versus standard acute hospital care for people after hip fracture surgery who have cognitive impairment: a feasibility cluster randomised controlled trial

Journal:	*BMJ Open*
Manuscript ID	bmjopen-2021-055267.R1
Article Type:	Original research
Date Submitted by the Author:	12-Jan-2022
Complete List of Authors:	Cross, Jane; University of East Anglia, Faculty of Medicine and Health Sciences<br>Hammond, Simon; University of East Anglia, Norwich Medical School<br>Shepstone, Lee; University of East Anglia, Norwich Medical School<br>Poland, Fiona; University of East Anglia, School of Health Sciences<br>Henderson, Catherine; London School of Economics,<br>Backhouse, Tamara; University of East Anglia, School of Health Sciences<br>Penhale, Bridget; University of East Anglia, School of Health Sciences<br>Donell, Simon; Norfolk and Norwich University Hospital<br>Knapp, Martin; London School of Economics, Personal Social Services Research Unit<br>Lewins, Douglas; University of East Anglia, Norwich Medical School<br>MacLullich, Alasdair; University of Edinburgh Western General Hospital, Geriatric Medicine Unit<br>Patel, Martyn; Norfolk and Norwich University Hospital<br>Sahota, Opinder; Nottingham University Hospitals NHS Trust, HCOP<br>Smith, Toby; University of East Anglia, Faculty of Medicine and Health Sciences; University of Oxford, Nuffield Department of Orthopaedics, Rheumatology and Musculoskeletal Sciences<br>Waring, Justin ; University of Birmingham<br>Howard, Robert; King's College London<br>Ballard, Clive ; University of Exeter, College of Medicine and Health<br>Fox, Chris; Norwich Medical School, Department of Psychological Sciences; University of Exeter,
**Primary Subject Heading**:	Geriatric medicine
Secondary Subject Heading:	Health services research
Keywords:	GERIATRIC MEDICINE, Rehabilitation medicine < INTERNAL MEDICINE, Hip < ORTHOPAEDIC & TRAUMA SURGERY, Delirium & cognitive disorders < PSYCHIATRY, REHABILITATION MEDICINE

SCHOLARONE™
Manuscripts

I, the Submitting Author has the right to grant and does grant on behalf of all authors of the Work (as defined in the below author licence), an exclusive licence and/or a non-exclusive licence for contributions from authors who are: i) UK Crown employees; ii) where BMJ has agreed a CC-BY licence shall apply, and/or iii) in accordance with the terms applicable for US Federal Government officers or employees acting as part of their official duties; on a worldwide, perpetual, irrevocable, royalty-free basis to BMJ Publishing Group Ltd ("BMJ") its licensees and where the relevant Journal is co-owned by BMJ to the co-owners of the Journal, to publish the Work in this journal and any other BMJ products and to exploit all rights, as set out in our licence.

The Submitting Author accepts and understands that any supply made under these terms is made by BMJ to the Submitting Author unless you are acting as an employee on behalf of your employer or a postgraduate student of an affiliated institution which is paying any applicable article publishing charge ("APC") for Open Access articles. Where the Submitting Author wishes to make the Work available on an Open Access basis (and intends to pay the relevant APC), the terms of reuse of such Open Access shall be governed by a Creative Commons licence – details of these licences and which Creative Commons licence will apply to this Work are set out in our licence referred to above.

Other than as permitted in any relevant BMJ Author's Self Archiving Policies, I confirm this Work has not been accepted for publication elsewhere, is not being considered for publication elsewhere and does not duplicate material already published. I confirm all authors consent to publication of this Work and authorise the granting of this licence.

For peer review only

BMJ OPEN

TITLE PAGE

Title:

PERFECTED enhanced recovery pathway (PERFECT-ER) versus standard acute hospital care for people after hip fracture surgery who have cognitive impairment: a feasibility cluster randomised controlled trial

Authors and Affiliations:

Jane L Cross[1] Simon P Hammond[2] Lee Shepstone[3] Fiona Poland[1] Catherine Henderson[4] Tamara Backhouse[1] Bridget Penhale[1] Simon Donell[5] Martin Knapp[4] Douglas Lewins[6] Alasdair MacLullich[7] Martyn Patel[5] Opinder Sahota[8] Toby O Smith[1] Justin Waring[9] Robert Howard[10] Clive Ballard[11] Chris Fox,[11*]

1 School of Health Sciences, University of East Anglia, Norwich Research Park, Norwich, UK

2 School of Education & Lifelong Learning, University of East Anglia, Norwich Research Park, UK

3 Norwich Medical School, University of East Anglia, Norwich Research Park, Norwich, UK

4 Care Policy and Evaluation Centre (CPEC), London School of Economics, London, UK

5 Norfolk and Norwich University Hospitals NHS Foundation Trust, Norwich, UK

6 Thornfield House, Gosberton, Spalding, UK

For peer review only - http://bmjopen.bmj.com/site/about/guidelines.xhtml

7 Geriatric Medicine, University of Edinburgh, Edinburgh, UK

8 Nottingham University Hospitals NHS Trust, Queens Medical Centre Campus, Nottingham, UK

9 Health Services Management Centre, University of Birmingham, Birmingham, UK

10 King's College London, UK

11 College of Medicine and Health, University of Exeter, UK

Corresponding author contact details:

Dr Jane L Cross

Email: j.cross@uea.ac.uk

For peer review only

Key words: Cognitive impairment, Dementia, hip fracture, Enhanced Recovery Pathway, acute hospital, cluster randomised controlled trial

Manuscript 3801 words

Abstract 300 words

Tables 4

Figures 1

Supplementary tables 1-9

For peer review only - http://bmjopen.bmj.com/site/about/guidelines.xhtml

ABSTRACT

Objectives: Assess feasibility of a cluster RCT to measure clinical and cost-effectiveness of an Enhanced Recovery Pathway for people with hip fracture and Cognitive Impairment (CI).

Design: Feasibility trial undertaken between 2016-18

Setting: Eleven acute hospitals from three UK regions.

Participants: 284 participants (208 female:69 male). Inclusion criteria: aged >60 years, confirmed proximal hip fracture requiring surgical fixation and CI; pre-operative AMTS ≤8 in England or a 4AT score ≥1 in Scotland; minimum of five days on study ward; a "suitable informant" able to provide proxy measures, recruited within seven days of hip fracture surgery. Exclusion criteria: no hip surgery; not expected to survive beyond four weeks; already enrolled in a clinical trial.

Intervention: PERFECT-ER, an enhanced recovery pathway with 15 quality targets supported by a checklist and manual, a Service Improvement Lead (SIL) a Process Lead (PPL) and implemented using a Plan Do Act model.

Primary and Secondary outcome measures: Feasibility outcomes: recruitment and attrition, intervention acceptability, completion of participant reported outcome measures, preliminary estimates of potential effectiveness using mortality, EQ-5D-5L, economic and clinical outcome scores.

Results: 282 participants were consented and recruited (132, intervention) from a target of 400. Mean recruitment rates were the same in intervention and control sites, (range:1.2 and 2.7 participants/month). Retention was 230(86%) at one month and 54%(144) at six months. At three months a relatively small effect (one quarter of a standard deviation) was observed on health-related quality of life of the patient measured with EQ-5D-5L proxy in the intervention group.

For peer review only - http://bmjopen.bmj.com/site/about/guidelines.xhtml

Conclusion: This trial design was feasible with modifications to recruitment. Mechanisms for delivering consistency in the PERFECT-ER intervention and participant retention need to be addressed. However, a RCT may be a sub-optimal research design to evaluate this intervention due to the complexity of caring for people with cognitive impairment after hip fracture.

Trial registration: PERFECTED CRCT ISRCTN99336264

Strengths and Limitations

- This feasibility RCT provides valuable evidence that the intervention and trial design can be delivered but would require a substantially larger number of trial sites and larger sample size.
- As only a small proportion of people of non-white ethnicity were recruited (patients and suitable informants) it is unclear how successful recruitment and retention of participants from wider ethnic backgrounds would be.
- The duration and type of cognitive impairment i.e. established dementia versus temporary delirium, was not controlled for within the analysis..
- Health economic data collection should be simplified and data extracted from hospital records to reduce burden on suitable informants.

INTRODUCTION

Hip fracture is associated with advancing frailty and has substantial impact on the health, well-being and independence of older people and their families (1, 2). Acute hip fracture care costs an estimated £1.1 billion per annum in the UK (3). In the 12 months after fracture,

For peer review only - http://bmjopen.bmj.com/site/about/guidelines.xhtml

patients are at increased risk of cognitive and functional decline, admission to long-term care institutions and higher mortality (4). People with cognitive impairment (CI) are amongst the most vulnerable in acute hospital settings (5), with lower short-term survival and 15% mortality during admission (4). They are susceptible to suboptimal and inconsistent care standards that contribute to cognitive deterioration, increase risk of post-operative complications, prolong length of stay and cause loss of independence (6).

In older adults with hip fracture, approximately 19% have dementia and up to 42% some degree of CI that may not meet criteria for a dementia diagnosis (7). People with hip fracture and CI are frequently cared for in environments which deliver excellent hip fracture care but are less skilled managing people with CI (8, 9). Hospital care of patients with CI remains an ongoing area of concern (5) with systemic failures in the care of older people repeatedly identified (10). Hospital staff may lack the knowledge and skills necessary to identify and assess CI, leading to under-identification which negatively affects access to rehabilitation services, supported discharge planning, person-centred care plans and involvement of families and carers (11-14).

This study assessed the feasibility of a cluster design randomised controlled trial (RCT) to measure the clinical and cost-effectiveness of an Enhanced Recovery Pathway versus standard care in acute hospitals for people after hip fracture surgery who demonstrate CI. Feasibility objectives included recruitment, retention, outcome selection, sample size estimation and acceptability of intervention training and delivery in NHS services.

METHODS

For peer review only - http://bmjopen.bmj.com/site/about/guidelines.xhtml

This paper has been prepared in accordance with the CONSORT Extension for Pilot and Feasibility Studies (15) reporting guideline. The study methods are summarised below and previously reported in detail (16).

Public and Patient Involvement

Patients and the pubic were involved from the conception of this study, through the review and funding process, the study, analysis and writing the findings. They were part of the steering, oversight and data monitoring groups.

Design and setting

A multi-centre, feasibility, cluster RCT was undertaken between 2016 and 2018. In line with MRC guidance for complex interventions, an integrated process evaluation was conducted (17); this is currently under review.

Randomisation

Randomisation was stratified by geographical area, with one intervention and one control hospital in UK region. Ten NHS hospitals were randomised to deliver experimental (PERFECT-ER) or control interventions. An additional site was recruited as a control group in July 2017 when another control site failed to recruit, and recruitment was extended from 10 months to 15 due to difficulties recruiting suitable informants. Recruitment was between November 2016 to February 2018.

Participants

For peer review only - http://bmjopen.bmj.com/site/about/guidelines.xhtml

Inclusion criteria

Participants were included if:

- Confirmed proximal hip fracture requiring surgery.
- Aged 60 years or over at the time of surgery.
- Pre-operative AMTS ≤8 in England (including those with zero because of an inability to answer questions) or a 4AT score ≥1 in Scotland.
- Minimum of five days on the study ward.
- Patient had a "suitable informant" (e.g. relative, unpaid or paid carer, care home manager) with a minimum of once a month face-to-face or telephone contact with the patient and able to provide proxy measures where required.
- Both patient and suitable informant to be recruited into the trial within seven days of the hip fracture surgery.

Exclusion criteria

Participants were excluded if:

- Did not undergo hip surgery.
- Patient not expected to survive beyond four weeks.
- Patient already enrolled in a clinical trial of an investigational medicinal product.

Sample Size

The target sample was 400 patient participants (200 per arm) from 10 centres (40 patient participants per site), based on the degree of precision for the estimated intra-class correlation coefficients (ICC). This was expected to provide a standard error for the ICC of between 0.033 and 0.041, for a true ICC value of between 0.05 and 0.10 for any endpoint. A priori, it was expected that four participants would be recruited per site, per month, over 10 months recruitment period.

For peer review only - http://bmjopen.bmj.com/site/about/guidelines.xhtml

Participant recruitment and consent

A three-step recruitment process was implemented, guided by previous phases of the PERFECTED programme, previous studies (18, 19) and input from clinical and academic collaborators:

1. Research nurses identified all new hip fracture admissions and screened for pre-recruitment eligibility in collaboration with clinical staff.
2. Patients (and where possible their potential suitable informant) were approached by the research nurse who provided study information as soon as clinically appropriate. Mental capacity was assessed by the research nurse, according to the appropriate legislative frameworks. In those lacking capacity to consent, consultee agreement from a relative or professional caregiver was sought, following the requirements of UK capacity legislation (20, 21).
3. The research nurse approached the patient and suitable informant to obtain written informed consent.

Intervention

Experimental intervention: PERFECT-ER

The PERFECT-ER is a multi-component intervention, implemented using service improvement principles, comprising:

- The PERFECT-ER checklist and manual.

For peer review only - http://bmjopen.bmj.com/site/about/guidelines.xhtml

- A Service Improve Lead (SIL) and PERFECTED Process Lead (PPL).
- A model for change (Plan-Do-Study-Act) (22).

The checklist has 15 organisational items, and 68 individual patient items grouped into three stages (Admission and Pre-Operative; Post-Operative and Rehabilitation; and Discharge), reflecting the patient journey through acute care settings. It was designed to identify areas of strength, and potential for improvement in practice, and overarches current hip fracture guidance. A comprehensive handbook explaining how to implement and use the intervention (the PERFECT-ER manual) was provided.

In the three months prior to recruitment commencing, the intervention was implemented in intervention sites by the SIL working 0.50 FTE, following the handbook and adherence assessed. When sites commenced recruitment, SIL resource was reduced to 0.2 FTE for the study period. A senior clinician (PPL) assisted the SILs for an hour a week to implement PERFECT-ER then an hour per month during recruitment.

Comparator group

The control group received treatment as usual. What this consisted of was recorded to determine local practice which followed NICE guidance for hip fracture care (23)

<u>Outcomes</u>

Data were collected from medical records of participating hospitals, the National Hip Fracture Database (NHFD) (24) and participants and suitable informants (summarised in **Supplementary Table 1)**. Study feasibility outcome measures included: recruitment and

For peer review only - http://bmjopen.bmj.com/site/about/guidelines.xhtml

attrition, intervention acceptability and fidelity, completion of participant reported outcome measures. The delivery of the intervention was monitored by auditing the patients notes against the PERFECT-ER checklist. Five patients per site were audited at the beginning of each implementation cycle and at the end of the trial: at three months pre-trial, 1.5 months pre-trial, trial baseline, four months, seven months, 10 months, 13 months, and 15 months. Clinical outcomes: mortality rate at 30 and 120 days; Bristol Activities of Daily Living Scale (BADLS); hospital admissions (number, length of stay and time to first admission); falls and mortality during previous six months; and the number of medications. Economic measures: quality-adjusted life years (QALY) of the participant (1) computed from DEMQOL-U and DEMQOL-PROXY-U) and (2) computed from EQ-5D-5L completed by participants and again by proxy, QALY of the suitable informant (unpaid carer), use of health, social and unpaid care collected via the Client Services Receipt Inventory (CSRI) (25) and hospital service use abstracted from hospital records. Costs of the intervention were assembled from time inputs of personnel providing PERFECT-ER, including time spent championing the ERP in study-set-up (**Supplementary Table 2**). Costs of inputs per site were calculated by dividing the costs of each role by the number of potentially affected patients on each study ward over the intervention period. Unit costs for other services were from published sources (26-29).

Statistical Analysis

Clinical outcome analysis

The data analyses summarise study process information including recruitment, participant 'flow' and retention, sample characteristics and completeness of baseline and follow-up outcome measures. To assess fidelity of the intervention the mean 'PERFECT-ER' score of enacted checklist items was determined.

For peer review only - http://bmjopen.bmj.com/site/about/guidelines.xhtml

For each outcome measure, at each follow-up point, an intra-class correlation coefficient (ICC) was calculated together with a 95% confidence intervals. These were calculated to assist the choice of primary outcome measure and inform potential sample size calculations for a definitive trial.

A precise estimate of intervention efficacy was not a primary objective of the data analyses. However, all efficacy outcome measures were modelled using a general linear model including the baseline value of the outcome (where available) and the treatment arm. Generalised Estimating Equations were used to account for 'clustering' created by the hospital level randomisation, thus accounting for the lack of independence of patient-level data within individual hospitals. The estimates of between arm difference are provided with 95% confidence intervals. The relationship between the individual 'PERFECT-ER' score and outcomes was considered and a Pearson correlation coefficient calculated to assess the strength of the linear relationship. The difference in mean 'PERFECT-ER' score between those known to have died during the study and those known to have survived was also calculated.

<u>Economic analysis</u>

The economic evaluation took an NHS and Personal Social Services (social care) perspective and a societal perspective, incorporating costs of unpaid care and out-of-pocket expenses (for equipment, adaptations, travel to healthcare appointments).

We computed utilities (to subsequently calculate QALYs) using societal weights (DEMQOL-U from the DEMQOL; DEMQOL-Proxy-U from the DEMQOL-Proxy; and EQ-5D-5L (30, 31). QALYs over the intervention period were derived using the trapezoid method to

For peer review only - http://bmjopen.bmj.com/site/about/guidelines.xhtml

approximate the area under the quality of life curve, with linear interpolation between time-points.

We examined the ICC of quality-adjusted life year (QALY) and total costs at six-month follow-up, with Searle's confidence intervals (using the arithmetic mean cluster size for unbalanced data) derived from one-way analysis of variance (32).

We examined the extent to which hospital services use extracted from hospital records gave the same estimates as data collected by suitable-informant-report. We examined the level of agreement on frequency of service use (counts) and total hospital costs between the two sources as estimated by Lin's concordance correlation coefficient (33). We also examined agreement between sources using the 95% limits of agreement approach (34), which calculates means and standard deviations of paired differences and the confidence interval for the difference, conditional on those differences being normally distributed and independent of the measures' magnitudes (35). Research nurses recorded the time taken to complete sections of the PERFECT-ER case report forms, covering multiple instruments/questions. To calculate a time-per-question estimate, the time taken to complete the CSRI, hospital use and medications review questions was divided by the number of items in the respective sections. Time taken to complete the measures was calculated by multiplying the total number of questions by the time-per-question.

Indicative cost-effectiveness analyses were conducted but are not reported here; details are available from the corresponding author.

RESULTS

Participant recruitment and retention

For peer review only - http://bmjopen.bmj.com/site/about/guidelines.xhtml

Figure 1 illustrates patient flow. Recruitment rate by centre is presented in **Table 1**. Hospital characteristics at baseline are described in **Supplementary Table 3** which shows sites in both intervention and control groups are broadly similar. 282 participants, 132 from intervention sites and 150 from control, were recruited. There were 151 months of site recruitment, 70 in intervention and 81 in control sites. Average recruitment rates did not differ between intervention and control sites, ranging from 1.2 to 2.7 participants/month. Mean recruitment rate was 1.87 per site/month. This contrasts with the expected four per site/month. The demographic characteristics of the 282 study participants and suitable informant characteristics are shown in **Table 2.**

Figure 1 here

Table 1: Recruitment Rates by Centre

Group	Site	Start Date	Months	Recruited	Rate / Month
**Intervention**			70	132	1.9
	01	December 2016	14	26	1.9
	03	November 2016	15	34	2.3
	06	November 2016	15	30	2.0
	07	February 2017	12	19	1.6
	10	December 2016	14	23	1.6
**Control**			81	150	1.9
	02	November 2016	15	24	1.6
	04	November 2016	15	18	1.2
	05	November 2016	15	23	1.5
	08	November 2016	15	35	2.3
	09	November 2016	15	40	2.7
	50	July 2017	6	10	1.7
**Total**			151	282	1.87

Table 2: Participant and suitable-informant baseline characteristics

Participant Characteristic	Intervention (N = 132)	Control (N = 150)	Total (N = 282)
Consent:			

For peer review only - http://bmjopen.bmj.com/site/about/guidelines.xhtml

Providing Own Consent	23 (17.6%)	38 (25.9%)	61 (21.9%)
Consultee / Legal Rep Consent	109 (82.4%)	112 (74.1%)	221 (78.1%)
Age (Mean (SD))	85.5 (7.4)	86.4 (7.9)	86.0 (7.6)
Missing	2	3	5
Gender:			
Male	37 (28.0%)	32 (22.1%)	69 (24.9%)
Female	95 (72.0%)	113 (77.9%)	208 (75.1%)
Missing	0	5	5
Ethnicity:			
Asian	1 (0.8%)	5 (3.4%)	6 (2.2%)
Black	1 (0.8%)	0	1 (0.4%)
White	106 (80.9%)	118 (80.8%)	224 (80.9%)
Unable to Respond	23 (17.6%)	23 (15.8%)	46 (16.6%)
Missing	1	4	5
Status:			
Married / Partner	40 (30.5%)	48 (32.7%)	88 (31.7%)
Divorced	7 (5.3%)	8 (5.4%)	15 (5.4%)
Single	6 (4.6%)	4 (2.7%)	10 (3.6%)
Widowed	54 (41.2%)	60 (40.8%)	114 (41.0%)
Unable to respond	24 (18.3%)	27 (18.4%)	51 (18.3%)
Missing	1	3	4
Employment Status:			
Employed	3 (2.3%)	3 (2.1%)	6 (2.2%)
Unemployed	3 (2.3%)	3 (2.1%)	6 (2.2%)
Retired	98 (74.8%)	107 (73.3%)	205 (74.0%)
Unable to respond	27 (20.6%)	33 (22.6%)	60 (21.7%)
Missing	1	4	5

**Suitable Informant Characteristic**	**Intervention (N = 132)**	**Control (N = 150)**	**Total (N = 282)**
Contact:			
Face-to-face	121 (91.7%)	129 (90.8%)	250 (91.2%)
Phone call	8 (6.1%)	11 (7.7%)	19 (6.9%)
Postal	3 (2.3%)	2 (1.4%)	5 (1.8%)
Missing	0	8	8
Relationship:			
Spouse	26 (19.8%)	26 (18.3%)	52 (19.0%)
Other Family Member	98 (74.8%)	110 (77.5%)	208 (76.2%)
Non-Family Member	4 (3.1%)	4 (2.8%)	8 (2.9%)
Paid Carer	3 (2.3%)	2 (1.4%)	5 (1.8%)
Missing	1	8	9
Age (Mean (SD))	60.7 (13.1)	62.2 (12.6)	61.5 (12.9)
Missing	4	10	14
Gender:			
Male	46 (34.8%)	63 (44.4%)	109 (39.8%)
Female	86 (65.2%)	79 (55.6%)	165 (60.2%)
Missing	0	8	8

For peer review only - http://bmjopen.bmj.com/site/about/guidelines.xhtml

Ethnicity:			
Asian	1 (0.8%)	7 (4.9%)	8 (2.9%)
Black	2 (1.5%)	0	2 (0.7%)
White	129 (97.7%)	135 (95.1%)	264 (96.4%)
Missing	0	8	8
Status:			
Married / Partner	98 (77.2%)	109 (77.3%)	207 (77.2%)
Divorced	11 (8.7%)	8 (5.7%)	19 (7.1%)
Single	15 (11.8%)	16 (11.3%)	31 (11.6%)
Widowed	3 (2.4%)	8 (5.7%)	11 (4.1%)
Missing	5	9	14
Employment Status:			
Employed	63 (48.1%)	54 (38.0%)	117 (42.9%)
Unemployed	11 (8.4%)	21 (14.8%)	32 (11.7%)
	57 (43.5%)	67 (47.2%)	124 (45.4%)
Retired	1	8	9
Missing			

For peer review only

Overall, the attrition rate was 50.7% (143/282). For the PERFECT-ER intervention attrition was 48.5% (64/132) and for control 52.7% (79/150).

Intervention Delivery

Although implementation was standardised across sites overall compliance with the intervention fluctuated over time and between sites. This is explored fully in the process evaluation (under review).

Missing Data

The degree of missing data varied across measures and across time-points. For example, baseline data collection consistently demonstrated high missingness for all outcomes (**Supplementary Table 4**). In contract, at discharge onwards, there were low missingness with the exception of the HowRwe at discharge EQ-5D-5L. Patient at one, three and six months,

For peer review only - http://bmjopen.bmj.com/site/about/guidelines.xhtml

and the Timed Up and Go at three months. The EQ-5D-5L for the suitable informant and proxy both demonstrated high missingness at six months in the intervention group (**Supplementary Table 4**).

Economic Outcomes

For economic data collection, there was relatively low occurrence of missing data for all health utilisation variables in primary care (6% to 8%) and hospital care, including both suitable informant-reported and hospital records-extracted use of emergency department, inpatient and outpatient services (4%-13%). Of a maximum of 23 medications reported, three to four costs were missing per case across the time points. More data were missing for suitable informant-reported unpaid care and lost working time. This was primarily because research nurses did not indicate whether the suitable informant was an unpaid or paid carer in 25% of cases at baseline and 17%, 15% and 13% of cases at one, three- and six-months follow-up respectively. Where the suitable informant was identified as an unpaid carer, rates of missingness in the unpaid carer questions were between 2% and 8% at the first three time points and 2% to 11% at six-month follow-up.

Clinical Outcome Feasibility

The baseline characteristics and outcomes are presented in **Tables 3** and **4**.

Table 3: Estimates of outcome

Time point & outcome measure	Intervention (N = 132) Mean (SD)	Control (N = 150) Mean (SD)	Adjusted difference [a]	95% Confidence Interval	p-value
**Baseline**					

For peer review only - http://bmjopen.bmj.com/site/about/guidelines.xhtml

HowRThey	4.96 (2.87)	4.55 (3.20)			
HowRwe	8.76 (2.38)	9.11 (2.23)			
EQ-5D – Patient	0.24 (0.37)	0.32 (0.36)			
**EQ-5D – SI**	0.80 (0.24)	0.85 (0.23)			
**EQ-5D – Proxy**	-0.01 (0.23)	0.15 (0.33)			
MMSE	12.2 (8.0)	10.8 (8.8)			
BADLS	24.3 (14.0)	21.0 (14.7)			
4AT	4.02 (3.33)	4.80 (4.02)			
CDR	1.63 (0.98)	1.41 (0.95)			
**Discharge**					
4AT	3.1 (2.7)	3.9 (3.4)	-0.45	(-1.23, 0.33)	0.255
HowRThey	3.3 (2.8)	2.5 (2.8)	0.52	(-0.65, 1.69)	0.387
HowRwe	8.9 (2.5)	9.1 (2.4)	-0.35	(-1.15, 0.44)	0.387
Length of stay	18.8 (10.2)	16.6 (12.0)	2.15	(-0.70, 5.01)	0.139
PERFECTER	0.75 (0.11)	0.74 (0.17)	0.059	(-0.10, 0.21)	0.450
**1 Month**					
BADLS	25.0 (12.5)	24.8 (13.6)	-1.50	(-4.56, 1.57)	0.338
EQ-5D SI	0.8 (0.2)	0.9 (0.2)	-0.029	(-0.066, 0.007)	0.113
EQ-5D by Proxy	0.2 (0.3)	0.3 (0.3)	0.028	(-0.042, 0.099)	0.434
EQ-5D Patient	0.6 (0.3)	0.5 (0.4)	0.074	(-0.078, 0.225)	0.341
HowRThey	4.8 (2.6)	4.0 (2.8)	0.601	(-0.040, 1.241)	0.066
MMSE	13.9 (8.0)	13.0 (7.9)	0.29	(-1.04, 1.62)	0.669
**3 Months**					
BADLS	24.6 (13.6)	22.4 (13.4)	-0.46	(-4.35, 3.42)	0.815
EQ-5D SI	0.8 (0.2)	0.9 (0.2)	-0.017	(-0.073, 0.039)	0.556
EQ-5D Proxy	0.3 (0.3)	0.3 (0.3)	0.071	(0.018, 0.124)	0.009
EQ-5D Patient	0.6 (0.3)	0.6 (0.4)	0.024	(-0.052, 0.101)	0.533
HowRThey	4.3 (2.5)	3.4 (2.9)	0.47	(-0.53, 1.47)	0.359
MMSE	13.6 (8.6)	12.5 (8.9)	0.75	(-0.77, 2.27)	0.333
Timed Up & Go	47.3 (33.3)	48.7 (28.1)	-1.54	(-15.38, 12.30)	0.827
**6 Months**					
BADLS	26.4 (14.2)	21.6 (12.0)	1.97	(-1.31, 5.25)	0.239
CDR Score (SI)	1.9 (1.1)	1.7 (1.0)	-0.015	(-0.160, 0.131)	0.845
EQ-5D SI	0.8 (0.2)	0.9 (0.2)	-0.016	(-0.096, 0.063)	0.688
EQ-5D by Proxy	0.4 (0.3)	0.3 (0.4)	0.099	(0.001, 0.198)	0.047
EQ-5D Patient	0.7 (0.3)	0.7 (0.3)	0.057	(-0.104, 0.218)	0.489
HowRThey	4.1 (2.7)	3.3 (2.7)	0.38	(-0.49, 1.25)	0.394
MMSE	13.1 (9.3)	12.2 (8.9)	0.69	(-1.14, 2.53)	0.457

Table 4: Mortality and discharge destination outcomes

**Mortality**	**Intervention (N = 132)(%)**	**Control (N = 150)(%)**	**Total (N = 282)(%)**
Death in hospital [a]	4 (4.0)	7 (5.7)	11 (4.9)
Death within 30 days of surgery [b]	8 (6.1)	9 (6.1)	17 (6.1)
Death within 6 months of surgery [b]	28 (21.4)	24 (16.2)	52 (18.4)
Total Deaths	30 (22.7)	27 (18.0)	57 (20.2)

**NHFD Discharge Destination [c]**	**Intervention**	**Control**	**Total**

For peer review only

For peer review only - http://bmjopen.bmj.com/site/about/guidelines.xhtml

	(N = 132)(%)	(N = 150)(%)	(N = 282)(%)
Died	4 (4.0)	7 (5.7)	11 (4.9)
Nursing Care	19 (19.0)	16 (13.0)	35 (15.7)
Other	3 (3.0)	1 (0.8)	4 (1.8)
Own Home/Sheltered Housing	36 (36.0)	58 (47.2)	94 (42.2)
Rehabilitation Unit (NHS funded care home bed)	0	8 (6.5)	8 (3.6)
Rehabilitation Unit (Hospital bed in another trust)	12 (12.0)	8 (6.5)	20 (9.0)
Residential Care	21 (21.0)	25 (20.3)	46 (20.6)
Unknown	5 (5.0)	0	5 (2.2)
Missing	32 (24.2)	27 (18.0)	59 (20.9)

a: From NHFD data, not available for 59 Scottish participants, 32 intervention and 27 control.
b: 3 patients (1 Intervention, 2 Control) included in 'total deaths' had missing surgery dates. These have not been included in the 'Death within 30 days of surgery' or the 'Death within 6 months of surgery' totals.
C: From NHFD data, not available for 59 Scottish participants, 32 intervention and 27 control.

For peer review only

Mortality

Over the duration of the trial, 57 participants (20%) died. A slightly higher rate was observed in the intervention group than in the control group, (23% versus 18%). Death in hospital was determined from National Hip Fracture Database (NHFD) data and only available for participants in England, thus excluding 59 Scottish participants. Eleven participants (5% of those with NHFD data) died in hospital with more in the control group (6% versus 4%). There were 17 (6%) patients who died within 30 days of surgery and 52 (18.4%) within six months.

Discharge destination

Place of discharge from hospital was identified from the NHFD data, thus unavailable for 59 Scottish participants. The largest proportion of participants returned to their own home or moved into sheltered housing (42%). This destination was more likely in the control group (47%) than the intervention group (36%).

For peer review only - http://bmjopen.bmj.com/site/about/guidelines.xhtml

Quality of life

No differences were seen in health-related quality of life (HRQOL) between the control group and intervention group at discharge or one-month follow-up. At three months, a potential beneficial effect of the intervention over control was evidenced for patient HRQOL based upon the EQ-5D-5L by proxy: those in the intervention group had a mean EQ-5D utility score 0.071 higher than control (95% confidence intervals: (0.018 to 0.124), p=0.009), a relatively small effect of around one quarter of a standard deviation. A difference of 0.099, in favour of the intervention group, was also seen at the six months follow-up (95% confidence intervals: (0.001 to 0.198), p=0.047).

Economic Outcome Feasibility

Intervention costs across the five study wards ranged from £131 to £485 per patient over the study period. (**Supplementary Table 5**). There were no significant differences in total costs between groups at any time point except in total health and social care (HSC) costs (including intervention costs) at 3 months using suitable informant reported data (£4004, 95% confidence intervals: £30 to £7979, p=0.049). Total costs (including intervention costs) at each time-point are summarised in **Supplementary Table 6**.

Total costs over the intervention period (**Supplementary Table 7)** differed depending on the perspective and the source of data on hospital utilisation. HSC costs based on suitable-informant-reported data, including or excluding intervention costs, were significantly higher in the intervention than control group. However groups did not differ on total societal costs, including or excluding intervention costs, regardless of source. Suitable informant data differed from the hospital records-extracted data in that it could include hospital stays from trusts other

For peer review only - http://bmjopen.bmj.com/site/about/guidelines.xhtml

thans those providing the hospital records, which may partly explain discrepancies between costs from different sources.

Group ICCs for 6-months costs and QALY are given in **Supplementary Table 8** . In the costs data, a pattern of negative ICC estimates indicated little clustering in the intervention group but some degree of clustering in the control group data. ICC for QALY ranged from 0.004 to 0.268 in the intervention and from -0.04 to 0.263 in the control group.

Concordance between hospital records-extracted and suitable-informant reported sources on frequency of hospital service use and costs was generally weak, although Lin coefficients ranged between ρ_c= 0.099 and ρ_c= 0.813 for service use across time points (**Supplementary Table 9**). Concordance on hospital costs was high at the baseline (ρ_c=0.660) but was ρ_c=0.379 at one-month and ρ_c<0.3 at three and six months. Limits of agreement showed that the two measures yielded estimates within £3,400 of each other at baseline, £7,000 at one-month and similar at six-months, but at three months the limits of agreement were much wider (-£8020 to £10,693).

Sample Size Calculation

ICCs were estimated, with 95% confidence intervals to inform a sample size calculation. The highest value was estimated for the PERFECT-ER score, 0.748, indicating a substantial degree of between-hospital variation compared to variation between-individuals within hospitals. This is not surprising given the intervention aimed to standardise practice within intervention hospitals thereby inflating the ICC. At follow-up time points, the ICCs typically ranged between 0.05 and 0.1. At six months, estimates for the MMSE and EQ-5D-5L by proxy were

For peer review only - http://bmjopen.bmj.com/site/about/guidelines.xhtml

negative and, since a negative value is theoretically not possible and results from estimation error, these were interpreted as being a 'small', positive value, near to zero.

DISCUSSION

The findings indicate that modifications are necessary to the trial design for a viable definitive trial. Whilst this study successfully demonstrated the ability to recruit from a variety of different UK sites, the rate was lower than anticipated. There was a lot of missing data for some measures, therefore steps to improve retention of participants at follow-up time-points is warranted, and a sufficiently large inflation of the sample size is required to compensate for missingness. Mortality has been suggested as an appropriate primary outcome. Economic data collection proved burdensome to suitable informants. A definitive trial should reduce this burden e.g. by extracting hospital services use data from hospital records.

We hypothesise short-term mortality (30-days) may be reduced by the PERFECT-ER intervention due to the cumulative effect of increased good practices across the range of care domains. This builds on previous work (10, 36-38) which recognises complex associations between hospitalisation, pre-admission cognitive impairment, post-admission cognitive impairment, functional decline and mortality. Through this, we would recommend mortality be a proposed primary outcome if a future definitive trial is undertaken.

Complex interventions that focus on staff quality improvement and associated implementation methods such as Plan Do Study Act methods (22) present challenges for investigation using RCTs (39). The management and care of people with dementia and CI with hip fracture is complex. This is an example of a 'wicked problem', defined as complex, messy and stubborn

For peer review only - http://bmjopen.bmj.com/site/about/guidelines.xhtml

challenges which continually evolve and has, at its core, many reasons for being, with no single solution which can be applied in all circumstances. Ultimately 'wicked problems' are those which cannot be reduced to a set of fixable problems and are often impossible to 'solve' because of incomplete, competing and changing requirements and where the solutions needed are "better or worse" rather than "right or wrong" (40-42). Whilst pragmatic RCTs, which offer tailoring and flexibility in experimental interventions, are one approach to testing management strategies for such healthcare challenges, other research methodologies may provide important insights. Further consideration of a range of methodological approaches may be more appropriate to answer this research question before automatically embarking on a clinical trial pathway.

CONCLUSION

This study has demonstrated that PERFECT-ER can be implemented and widely accepted across a number of different health services in the UK's NHS. We have shown it is feasible, with modifications, to undertake a definitive trial and economic evaluation using the developed and refined recruitment and consenting practices. However, care of people with CI and hip fracture poses a 'wicked problem' and further definitive research using a RCT approach should be deliberated against other methods of evaluation.

DECLARATIONS

Ethical Approval: Ethical approval for the trial was given by Camden and Kings Research Ethics Committee (reference number: 16/LO/0621) and Scotland Research Ethics Committee A (reference number: 16/SS/0086).

For peer review only - http://bmjopen.bmj.com/site/about/guidelines.xhtml

Trial registration number: ISRCTN 99336264.

Funding: This work was supported by NIHR Programme Grants for Applied Research (PGfAR) Programme grant number [ref: DTC-RP-PG-0311-12004].

For peer review only

For peer review only - http://bmjopen.bmj.com/site/about/guidelines.xhtml

FIGURE AND TABLE LEGENDS

Figure 1: Patient Flow Diagram

Table 1: Recruitment rates by Centre

Table 2: Participant and suitable informant baseline characteristics

Table 3: Estimates of outcome

Table 4: Mortality and discharge destination outcomes

For peer review only

For peer review only - http://bmjopen.bmj.com/site/about/guidelines.xhtml

Supplementary Table 1: Data collection schedule

Supplementary Table 2: Per-site cost of 3 months start-up and 15 months of input from PERFECT-ER SIL AND PIL**Supplementary Table 2:** Hospital baseline characteristics**Supplementary Table 3:** Available data for analysis

Supplementary Table 5: Per-site costs over the study period (1/11/2016 – 31/1/2018)

Supplementary Table 4: Mean costs (standard errors): Health & social care services for participant, unpaid carer (SI) costs, out-of-pocket costs, total health & social care and societal costs over prior three months, at baseline and one-, three-, and six-month follow-ups (£, 2016-17).

Supplementary Table 7: Mean six-month costs (excluding or including intervention costs) over the study period (£, 2016-16 prices). Sample: cases where total costs were available across follow-up assessments

Supplementary Table 8: Intra-class correlations of 6-month total health and social care and societal costs (£,2016-17) and QALY over 6 months. Sample: cases where costs or outcomes data were available at all study period time points

Supplementary Table 9: Agreement between hospital records-extracted and self-report hospital service use and costs.

For peer review only

For peer review only

For peer review only - http://bmjopen.bmj.com/site/about/guidelines.xhtml

Contributorship statement

JLC, SPH, LS, FP, CH, TB, BP, SD, DL, MK, AM, MP, OS, TS, JW, RH, CB, and CF made substantial contributions to the conception or design of the work;

DL provided PPI input thoroughout the study

· SPH, TB led on the acquisition of the data

·, LS, CF, SPH and JC led the statistical analysis and interpretation of data for the work;

· JC led the drafting of the paper. All authors were involved in revising it critically for important intellectual content;

· All authors reviewed the paper and gave their final approval of the version to be published;

· All authors give their agreement to be accountable for all aspects of the work in ensuring that questions related to the accuracy or integrity of any part of the work are appropriately investigated and resolved.

There are no competing interests for any author

Data availability statement

Data are available on reasonable request to the corresponding author

For peer review only

References

1. Ravindrarajah R, Hazra NC, Charlton J, Jackson SHD, Dregan A, Gulliford MC. Incidence and mortality of fractures by frailty level over 80 years of age: cohort study using UK electronic health records. BMJ Open. 2018;8(1):e018836.
2. Griffin XL, Parsons N, Achten J, Fernandez M, Costa ML. Recovery of health-related quality of life in a United Kingdom hip fracture population. The Warwick Hip Trauma Evaluation--a prospective cohort study. Bone Joint J. 2015;97-B(3):372-82.
3. Leal J, Gray AM, Prieto-Alhambra D, Arden NK, Cooper C, Javaid MK, et al. Impact of hip fracture on hospital care costs: a population-based study. Osteoporos Int. 2016;27(2):549-58.
4. Sampson EL, Leurent B, Blanchard MR, Jones L, King M. Survival of people with dementia after unplanned acute hospital admission: a prospective cohort study. Int J Geriatr Psychiatry. 2013;28(10):1015-22.

For peer review only - http://bmjopen.bmj.com/site/about/guidelines.xhtml

5. Boaden A. Fix Dementia Care Hospitals: Alzheimer's Society 2016 [Available from: https://www.alzheimers.org.uk/sites/default/files/migrate/downloads/fix_dementia_care_-_hospitals.pdf.
6. Fogg C, Meredith P, Bridges J, Gould GP, Griffiths P. The relationship between cognitive impairment, mortality and discharge characteristics in a large cohort of older adults with unscheduled admissions to an acute hospital: a retrospective observational study. Age Ageing. 2017;46(5):794-801.
7. Seitz DP, Adunuri N, Gill SS, Rochon PA. Prevalence of dementia and cognitive impairment among older adults with hip fractures. J Am Med Dir Assoc. 2011;12(8):556-64.
8. Myers JS, Grigsby J, Teel CS, Kramer AM. Nurses' assessment of rehabilitation potential and prediction of functional status at discharge from inpatient rehabilitation. Int J Rehabil Res. 2009;32(3):264-6.
9. Featherstone K, Northcott A, Bridges J, Harrison Denning K, Harden J, Bale S, et al. Summary Results: An evidence based investigation examining the care people living with dementia receive following an acute hospital admission.: London: Stories of Dementia; 2018 [Available from: http://www.storiesofdementia.com/2018/04/research-report.html.
10. Royal College of Psychiatrists, National Audit of Dementia (Care in General Hospitals) Spotlight Audit: Assessment of delirium in hospital for people with dementia 2018 [Available from: https://www.hqip.org.uk/wp-content/uploads/2018/08/NAD-Delirium-Spotlight-report-Aug-2018.pdf.
11. Gupta A, Havelock W. A new future for hip fracture care--orthogeriatrician lead in an 'Acute' Hip Unit. Clin Med (Lond). 2014;14(6):591-6.
12. Riemen AH, Hutchison JD. The multidisciplinary management of hip fractures in older patients. Orthop Trauma. 2016;30(2):117-22.
13. National Collaborating Centre for Mental Health (UK), Dementia: A NICE-SCIE Guideline on Supporting People With Dementia and Their Carers in Health and Social Care. British Psychological Society. 2007 [Available from: https://www.scie.org.uk/publications/misc/dementia/dementia-fullguideline.pdf?res=true.
14. Gill N, Hammond S, Cross J, Smith T, Lambert N, Fox C. Optimising care for patients with cognitive impairment and dementia following hip fracture. Z Gerontol Geriatr. 2017;50(Suppl 2):39-43.
15. Schulz KF, Altman DG, Moher D. CONSORT 2010 statement: updated guidelines for reporting parallel group randomized trials. Ann Intern Med. 2010;152(11):726-32.
16. Hammond SP, Cross JL, Shepstone L, Backhouse T, Henderson C, Poland F, et al. PERFECTED enhanced recovery (PERFECT-ER) care versus standard acute care for patients admitted to acute settings with hip fracture identified as experiencing confusion: study protocol for a feasibility cluster randomized controlled trial. Trials. 2017;18(1):583-.
17. Moore GF, Audrey S, Barker M, Bond L, Bonell C, Hardeman W, et al. Process evaluation of complex interventions: Medical Research Council guidance. BMJ. 2015;350:h1258.
18. Harwood RH, Goldberg SE, Whittamore KH, Russell C, Gladman JR, Jones RG, et al. Evaluation of a Medical and Mental Health Unit compared with standard care for older people whose emergency admission to an acute general hospital is complicated by concurrent 'confusion': a controlled clinical trial. Acronym: TEAM: Trial of an Elderly Acute care Medical and mental health unit. Trials. 2011;12:123.
19. Young J, Cheater F, Collinson M, Fletcher M, Forster A, Godfrey M, et al. Prevention of delirium (POD) for older people in hospital: study protocol for a randomised controlled feasibility trial. Trials. 2015;16:340.
20. Mental Capacity Act 2005. London: HMSO; 2005.
21. Adults with Incapacity (Scotland) Act 2000. In: Parliment S, editor.: HMSO; 2000.

For peer review only - http://bmjopen.bmj.com/site/about/guidelines.xhtml

22. NHS Improvement. *Plan, do, study, act (PDSA) cycles and the model for improvement* 2018 [Available from: https://improvement.nhs.uk/documents/2142/plan-do-study-act.pdf
23. NICE. Hip fracture: management Clinical guideline [CG124] London: NICE; 2011 [updated 2017. Available from: https://www.nice.org.uk/Guidance/CG124.
24. https://www.nhfd.co.uk/ [Internet]. [cited 30 6 2021].
25. Beecham JK, Knapp MRJ. Costing psychiatric interventions. In: Thornicroft G, Brewin C, Wing JK, editors. Measuring Mental Health Needs. 2nd ed. London: Gaskell; 2001. p. 220-4.
26. Curtis L, Burns A. Unit Costs of Health and Social Care 2017. Canterbury: Personal Social Services Research Unit, University of Kent; 2017.
27. NHS Improvement NHS trusts and NHS foundation trusts. [Internet]. 2017. Available from: https://improvement.nhs.uk/resources/reference-costs/.
28. https://www.england.nhs.uk/pay-syst/national-tariff/NHS Improvement and NHS England. National Tariff: currencies and prices. [Internet]. 2017/18 [cited 1 7 2021]. Available from: https://improvement.nhs.uk/documents/1044/2017-18_and_2018-19_National_Tariff_Payment_System.pdf.
29. NHS Digital. Prescription cost analysis England 2017 [Internet]. 2018. Available from: https://digital.nhs.uk/data-and-information/publications/statistical/prescription-cost-analysis/prescription-cost-analysis-england-2017.
30. Rowen D, Mulhern B, Banerjee S, Hout B, Young TA, Knapp M, et al. Estimating preference-based single index measures for dementia using DEMQOL and DEMQOL-Proxy. Value Health. 2012;15(2):346-56.
31. van Hout B, Janssen MF, Feng YS, Kohlmann T, Busschbach J, Golicki D, et al. Interim Scoring for the EQ-5D-5L: Mapping the EQ-5D-5L to EQ-5D-3L Value Sets. Value in Health. 2012;15(5):708-15.
32. Ukoumunne OC. A comparison of confidence interval methods for the intraclass correlation coefficient in cluster randomized trials. Statistics in Medicine. 2002;21(24):3757-74.
33. Lin LI. A concordance correlation coefficient to evaluate reproducibility. Biometrics. 1989;45(1):255-68.
34. Bland JM, Altman DG. Statistical Methods for Assessing Agreement between Two Methods of Clinical Measurement. Lancet. 1986;1(8476):307-10.
35. Steichen TJ, Cox NJ. sg84: Concordance correlation coefficient. Stata Technical Bulletin Reprints. 1998;8:137-43.
36. Dementia Action Alliance 2018 [Available from: https://www.dementiaaction.org.uk/dementiafriendlyhospitalscharter.
37. Health Foundation Spotlight on Dementia Care 2011 [Available from: https://www.health.org.uk/sites/default/files/SpotlightOnDementiaCare_fullversion.pdf.
38. Department of Health Living well with dementia: A National Dementia Strategy 2009 [Available from: https://assets.publishing.service.gov.uk/government/uploads/system/uploads/attachment_data/file/168220/dh_094051.pdf.
39. Paparini S, Green J, Papoutsi C, Murdoch J, Petticrew M, Greenhalgh T, et al. Case study research for better evaluations of complex interventions: rationale and challenges. BMC Medicine. 2020;18(1):301.
40. Buchanan R. Wicked Problems in Design Thinking. Design Issues. 1992;8(2):5-21.
41. Horst WJR, Webber MM. Dilemmas in a General Theory of Planning. Policy Sciences. 1973;4(2):155-69.

For peer review only - http://bmjopen.bmj.com/site/about/guidelines.xhtml

42. Krippendorff K. The Semantic Turn: A New Foundation for Design (1st ed.). . Boca Raton: CRC Press; 2005. 368 p.

For peer review only - http://bmjopen.bmj.com/site/about/guidelines.xhtml

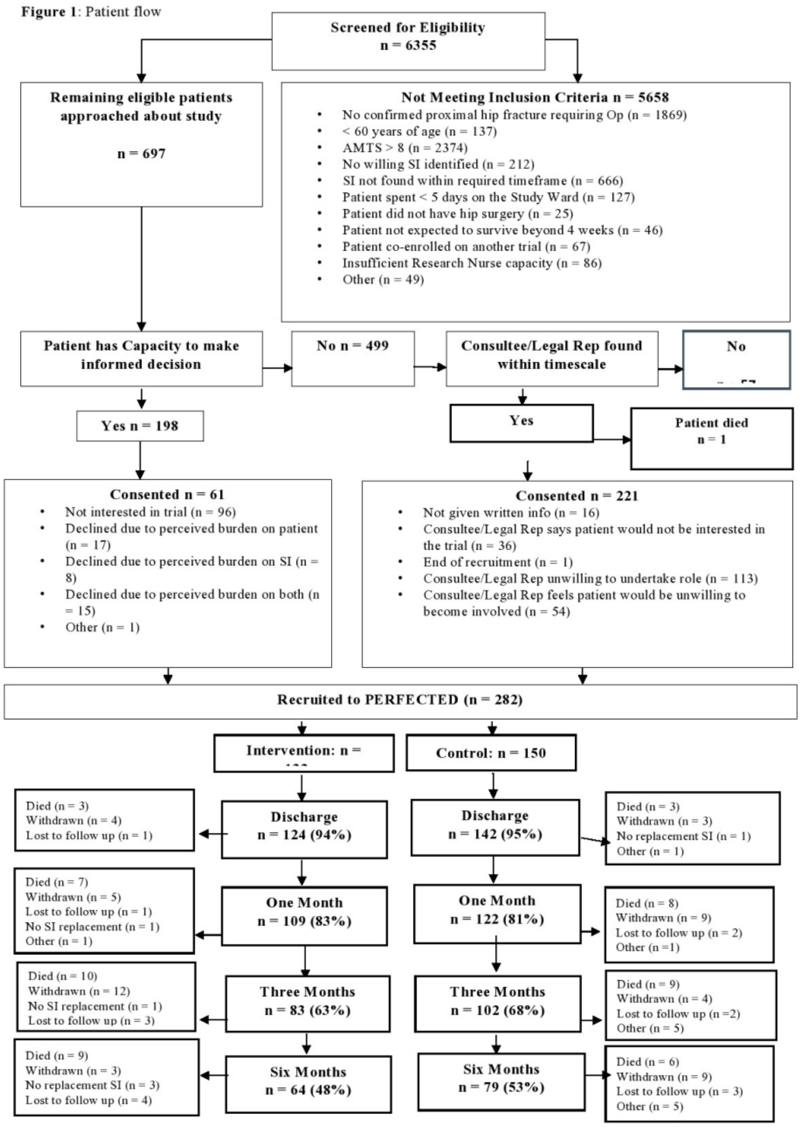

Figure 1 Patient Flow Diagram Statement of authorship: Figure created by the authors

104x148mm (300 x 300 DPI)

Supplementary Table 1: Data collection schedule:

Statement of authorship: Table created by the authors

	Admission	Enrolment	Baseline	Post-operative period			
**TIMEPOINT**	**-T2**	**-T1**	**0**	**D**	**T1[a]**	**T2**	**T3**
PRE-INTERVENTION:							
Eligibility screen							
Study information provided							
Informed consent given							
ASSESSMENTS:							
MMSE-2: SV (Patient)							
DEMQOL (Patient)							
EQ-5D-5L self-complete (Patient)							
howRwe (Patient)				b	b		
CDR (Patient)							
Patient care profile (Patient)				b	b		
Timed Up & Go (Patient)							
BADLS (Suitable Informant)							
DEMQOL-Proxy (Suitable Informant)							
EQ-5D-5L Proxy (Suitable Informant)							
EQ-5D-5L Carer self-report (Suitable Informant)							
CSRI[c] (Suitable Informant)							
Number of days in institutional care (Suitable Informant)							
howRthey (Suitable Informant)				b	b		
Patient's place of residence (Suitable Informant)			d				
CDR (Suitable Informant)							
IQCODE (Suitable Informant)							
Length of stay in index hospitalisation				e	e		
Discharge destination from index hospitalisation							
Mortality							
Hospital re-admission rates							
Hospital service use[f]							
4AT				b	b		
Charlson Co-morbidity Index (CCI)							
NHFD (England only)							g

[a] PERFECT-ER and treatment as usual continue up until discharge from study ward. Due to differences in length of stay in the study sites, T1 assessments may take place in the study site for some participants;
[b] Patients may be discharged from study ward before or after T1. Measure to be collected at whenever this point maybe ± five days;
[c] duration of retrospective period covered varies by assessment point;
[d] pre-baseline ordinary residence;
[e] If patient is still in acute hospital at thirty days this will be recorded;
[f] from hospital patient records, of service use within site of index hospitalisation
[g] extracted from NHFD post recruitment window closing

For peer review only

Supplementary Table 2. Per-site cost of 3 months start-up and 15 months of input from PERFECT-ER SIL AND PIL

**Per site**			
**SIL**	**% of year**	**Period FTE**	**Annual FTE**
Champion ERP 1st August to 31st October 2016	0.25	0.5	0.125
First year: 1/11/2016 - 31/7/2017	0.75	0.2	0.15
Second year: 1/8/2017 - 31/1/2018	0.5	0.2	0.1
Total FTE @£70,017 per annum (2016-17 prices)[a]	**£26,594**		
**PPL**	**Hours**		
First year: 1 hour/week for 3 months	13		
First year: 1 hour/month for 9 months	9		
Second year: 1 hour/month for 6 months	6		
Total hours PPL input	28		
Total hours @£106 per hour (2016-17 prices)[b]	**£2,968**		

[a]source: Schema 14: Hospital Nurses, AfC band 6[25]
[b]source: Schema 15. Hospital-based doctors, Medical Consultant[25]

For peer review only

Supplementary Table 3: Hospital baseline characteristics

	Intervention			Control [a,b]		
	**Median**	**Max**	**Min**	**Median**	**Max**	**Min**
Number of Beds on Ward	27.0	41.0	15.0	28.0	38.0	25.0
Number of Bed Days on Ward in last 12 months	9855.0	14965.0	5475.0	10220.0	13870.0	9038.0
Occupied Bed Rate (%) in last 12 months	93.0	99.0	90.0	96.0	100	93.0
Number of Falls on Ward in last 12 months	42.0	82.0	25.0	60.0	111.0	32.0
Number of Deaths on Ward in last 12 months	30.0	66.0	7.0	34.0	68.0	13.0
Registered/Qualified Nurses	22.0	27.5	16.2	19.8	26.8	12.0
Geriatricians	1.0	2.6	0.5	1.0	1.0	0.8
Orthopaedic Surgeons	0.3	1.0	0.0	1.5	12.0	0.0
Other Consultants	0.0	0.4	0.0	0.0	4.7	0.0
Other Registrars	0.5	1.0	0.0	1.0	5.6	0.4
Other Junior Doctors	1.5	2.5	0.0	3.0	3.0	1.0

a One hospital (Control) missing all data
b One hospital (Control) missing data for *Number of Falls on the Ward* in last 12 months.

For peer review only

For peer review only

Supplementary Table 4: Available data for analysis

Time point & outcome measure	Intervention (N = 132)	Control (N = 150)
**Baseline**		
HowRThey	5 (3.8)	13 (8.7)
HowRwe	39 (29.5)	56 (37.3)
EQ-5D – Patient	40 (30.3)	63 (42.0)
EQ-5D – SI	7 (5.3)	11 (7.3)
EQ-5D – Proxy	6 (4.5)	14 (9.3)
MMSE	4 (3.0)	13 (8.7)
BADLS	5 (3.8)	9 (6.0)
4AT	5 (3.8)	18 (12.0)
CDR	5 (3.8)	13 (8.7)
**Discharge**	**Expected = 123**	**Expected = 143**
HowRthey	116 (94.3)	116 (81.1)
HowRwe	84 (68.3)	72 (50.3)
4AT	116 (94.3)	103 (72.0)
Length of Stay	121 (98.4)	142 (99.3)
PERFECTER Score	122 (99.2)	141 (98.6)
**1 Month**	**Expected = 108**	**Expected = 122**
MMSE	106 (98.1)	111 (91.0)
BADLS	104 (96.3)	112 (91.8)
EQ-5D Patient	84 (77.8)	78 (63.9)
EQ-5D SI	106 (98.1)	110 (90.2)
EQ-5D Proxy	105 (97.2)	112 (91.8)
HowRthey	102 (94.4)	110 (90.2)
**3 Months**	**Expected = 83**	**Expected = 102**
MMSE	81 (97.6)	97 (95.1)
Timed Up & Go	44 (53.0)	50 (49.0)
BADLS	81 (97.6)	96 (94.1)
HowRthey	82 (98.8)	94 (92.2)
EQ-5D Patient	61 (73.5)	69 (67.6)
EQ-5D SI	81 (97.6)	97 (95.1)
EQ-5D Proxy	82 (98.8)	98 (96.1)
**6 Months**	**Expected = 64**	**Expected = 80**
MMSE	63 (98.4)	72 (90.0)
BADLS	61 (95.3)	77 (96.3)
HowRthey	64 (100)	76 (95.0)
EQ-5D Patient	36 (56.3)	43 (53.8)
EQ-5D SI	48 (75.0)	65 (81.3)
EQ-5D Proxy	44 (68.8)	65 (81.3)
Global CDR	64 (100)	66 (82.5)

a: Estimated as negative

For peer review only

For peer review only

Supplementary Table 5. Per-site costs over the study period (1/11/2016 – 31/1/2018)

Site	Estimated total numbers of potentially affected patients[a]	SIL cost per case on study ward	PPL cost per case on study ward	Total costs per potentially affected patient
**01**	190	£140	£16	£156
**03**	205	£130	£14	£144
**06**	76	£350	£39	£389
**07**	61	£436	£49	£485
**10**	225	£118	£13	£131

[a]Patients on study wards, 60≥, with confusion (AMTS≤8/4AT≥), hip fracture, surgery for hip fracture, ward stay of≥ 5 days.

For peer review only

Supplementary Table 6: Mean costs (standard errors): Health & social care services for participant, unpaid carer (SI) costs, out-of-pocket costs, total health & social care and societal costs over prior three months, at baseline and one-, three-, and six-month follow-ups (£, 2016-17)

Cost	Intervention (n=132)			Control (n=150)			Intervention-control	
	n	Mean	SE	n	Mean	SE	Mean difference	95% CI
**Baseline**								
Health & social care (HRE)	125	3740	709	135	3196	691	544	-1697, 2784
Health & social care (SIR)	123	3458	653	130	3148	642	310	-1761, 2381
Health & social care (SIR+)	125	3544	663	135	3094	645	450	-1642, 2543
Societal (HRE)[f]	95	9661	949	100	9783	932	-122	-3131, 2886
Societal (SIR)[f]	93	9249	946	97	9823	934	-574	-3581, 2433
Societal (SIR+)[f]	95	9299	886	100	9635	867	-336	-3140, 2469
**1 month**								
Intervn.+Health & social care (HRE)	89	12859	531	99	11636	509	1223	-441, 2886
Intervn.+Health & social care (SIR)	89	13890	980	95	11489	974	2401	-726, 5527
Intervn.+Health & social care (SIR+)	89	13894	945	99	11574	922	2320	-667, 5306
Intervn.+Societal (HRE)[f]	75	14191	526	80	13988	511	203	-1456, 1862
Intervn.+Societal (SIR)[f]	75	15032	1023	76	14123	1023	908	-2364, 4180
Intervn.+Societal (SIR+)[f]	75	15036	1023	80	14141	1000	895	-2341, 4131
**3 months**								
Intervn.+Health & social care (HRE)	75	9193	1721	88	5946	1684	3247	-2200, 8695
Intervn.+Health & social care (SIR)	75	8315	1258	87	4310	1226	4004*	30, 7979
Intervn.+Health & social care (SIR+)	75	8325	1274	88	4621	1236	3704	-311, 7719
Intervn.+Societal (HRE)[f]	64	12794	1909	71	10748	1846	2047	-3961, 8054
Intervn.+Societal (SIR)[f]	64	11983	1341	70	8923	1297	3060	-1161, 7281
Intervn.+Societal (SIR+)[f]	64	11995	1293	71	9243	1243	2752	-1305, 6808
**6 months**								
Intervn.+Health & social care (HRE)	57	6807	1402	64	5146	1413	1661	-2842, 6164
Intervn.+Health & social care (SIR)	57	6827	999	64	4308	965	2519	-624, 5661
Intervn.+Health & social care (SIR+)	57	6839	1004	64	4308	971	2531	-629, 5692
Intervn.+Societal (HRE)[f]	52	11511	1462	54	12478	1476	-967	-5666, 3733
Intervn.+Societal (SIR)[f]	52	11514	1506	54	11483	1536	31	-4836, 4897
Intervn.+Societal (SIR+)[f]	52	11528	1511	54	11483	1541	44	-4839, 4928

Note: NHS CC=NHS continuing care; HRE=health records extraction; SIR=Suitable Informant-reported; SIR+= corresponding hospital costs data from HRE used when costs were missing from the SIR dataset; Intervn.=Intervention costs

a Funded by NHS or Social Services

b Provided by NHS or Social Services

c expenditure by self or family on equipment purchases

d expenditure by self or family on travel to appointments

e unpaid carers' time in care and support to participant

f societal costs include: participant's health and social care costs; unpaid carers' time in care and support to participant; expenditure by self or family on travel to appointments, equipment purchases

For peer review only

For peer review only

Supplementary Table 7. Mean six-month costs (excluding or including intervention costs) over the study period (£, 2016-16 prices). Sample: cases where total costs were available across follow-up assessments

**Costs**	**Intervention**			**Control**			**Intervention - Control**	
	**n**	**Mean**	**SE**	**n**	**Mean**	**SE**	**Mean difference**	**95% CI**
Health & social care (HRE)	47	25 414	2 227	56	21 164	2 142	4 250	-2 739, 11 239
Health & social care (SIR)	47	26 304	1 741	53	18 930	1 639	7 373*	1 964, 12 782
Health & social care (SIR+)	47	26 342	1 731	56	19 231	1 586	7 111*	1 800, 12 422
Societal (HRE)[a]	39	35 837	3 118	38	38 067	3 227	-2 230	-12 578, 8 118
Societal (SIR)[a]	39	36 478	3 104	36	35 104	3 325	1 374	-9 115, 118 63
Societal (SIR+)[a]	39	36 524	3 235	38	35 067	3 358	1 456	-9 295, 12 208
Intervn.+Health & social care (HRE)	47	25 677	2 251	56	21 164	2 172	4 513	-2 563, 11 588
Intervn.+Health & social care (SIR)	47	26 567	1 744	53	18 930	1 642	7 636*	2 217, 13 055
Intervn.+Health & social care (SIR+)	47	26 605	1 734	56	19 231	1 589	7 374*	2 053, 12 695
Intervn.+Societal (HRE)[a]	39	36 080	3 142	38	38 067	3 253	-1 987	-12 416, 8 442
Intervn.+Societal (SIR)[a]	39	36 721	3 127	36	35 104	3 350	1 618	-8 951, 12 186
Intervn.+Societal (SIR+)[a]	39	36 767	3 256	38	35 067	3 381	1 700	-9 124, 12 523

Note: NHS CC=NHS continuing care; HRE=health records extraction; SIR=Suitable Informant-reported; SIR+= corresponding hospital costs data from HRE used when costs were missing from the SIR dataset; Intervn.=Intervention costs

* $p<0.05$

a. societal costs include: participant's health and social care costs; unpaid carers' time in care and support to participant; expenditure by self or family on travel to appointments, equipment purchases

For peer review only

Supplementary Table 8. Intra-class correlations of 6-month total health and social care and societal costs (£,2016-17) and QALY over 6 months. Sample: cases where costs or outcomes data were available at all study period time points

	Intervention (n=132) (N=5)				Control (n=150) (N=6)			
	**n**	**N**	**Mean**	**95% CI**	**n**	**N**	**Mean**	**95% CI**
Costs								
Health & social care (HRE)[d]	47	5	-0.045	-0.148 to 0.057	56	6	0.117	-0.152 to 0.386
Health & social care (SIR)[d]	47	5	-0.051	-0.147 to 0.045	53	6	0.034	-0.165 to 0.232
Health & social care (SIR+)[d]	47	5	-0.050	-0.147 to 0.048	56	6	0.028	-0.154 to 0.210
Societal (HRE)[g]	39	5	-0.041	-0.194 to 0.112	38	5	0.190	-0.189 to 0.569
Societal (SIR)[g]	39	5	-0.057	-0.194 to 0.079	36	5	0.214	-0.201 to 0.628
Societal (SIR+)[g]	39	5	-0.055	-0.194 to 0.084	38	5	0.240	-0.169 to 0.649
Intervention + Health & social care (HRE)[d]	47	5	-0.039	-0.149 to 0.071	56	6	0.117	-0.152 to 0.386
Intervention + Health & social care (SIR)[d]	47	5	-0.044	-0.148 to 0.059	53	6	0.033	-0.165 to 0.232
Intervention + Health & social care (SIR+)[d]	47	5	-0.043	-0.148 to 0.061	56	6	0.028	-0.154 to 0.210
Intervention +Societal (HRE)[g]	39	5	-0.033	-0.195 to 0.128	38	5	0.190	-0.189 to 0.569
Intervention +Societal (SIR)[g]	39	5	-0.049	-0.194 to 0.096	36	5	0.214	-0.201 to 0.628
Intervention +Societal (SIR+)[g]	39	5	-0.047	-0.194 to 0.101	38	5	0.240	-0.169 to 0.649
QALY								
Participant 6-month QALY (EQ-5D-5L)	30	5	0.268	-0.173 to 0.710	31	4	0.263	-0.236 to 0.762
Participant 6-month QALY (EQ-5D-5L-Proxy)	42	5	0.068	-0.181 to 0.316	62	6	0.110	-0.136 to 0.355
Participant 6-month QALY (DEMQOL-U)	34	5	0.236	-0.190 to 0.662	34	5	-0.001	-0.255 to 0.253
Participant 6-month QALY (DEMQOL-PROXY)	60	5	0.004	-0.121 to 0.129	67	6	0.037	-0.125 to 0.198
SI 6-month QALY (EQ-5D-5L)	48	5	0.255	-0.109 to 0.619	63	6	-0.040	-0.135 to 0.055

Note: HRE=health records extraction; SIR=Suitable Informant-reported; SIR+= hospital costs data from HRE used when these costs were missing from SIR dataset; SI=suitable informant; n=number of observations; N=number of clusters

For peer review only

Supplementary Table 9. Agreement between hospital records-extracted and self-report hospital service use and costs.

Item	Period	Mean Difference (SD) (HRE – SIR)	ρ_c (95% CI)	95% limits of agreement	Exact (none) % (N)	Exact (some) % (N)	Under % (N)	Over % (N)
**A&E visits**	Time 0	-0.339 (2.945)	0.099 (0.061, 0.136)	-6.110, 5.433	77 (198)	9 (23)	4 (10)	10 (26)
	Time 1	-0.015 (0.304)	0.452 (0.343, 0.561)	-0.611, 0.581	90 (186)	3 (7)	2 (5)	4 (8)
	Time 2	-0.124 (0.908)	0.308 (0.218, 0.397)	-1.903, 1.655	78 (132)	8 (14)	5 (8)	9 (15)
	Time 3	-0.143 (0.817)	0.367 (0.249, 0.485)	-1.744, 1.458	75 (95)	15 (19)	2 (2)	8 (10)
**Admissions**	Time 0	0.100 (0.630)	0.620 (0.462, 0.777)	-1.134, 1.334	38 (23)	27 (16)	22 (13)	13 (8)
	Time 1	0.108 (0.350)	0.454 (0.350, 0.557)	-0.577, 0.794	-	90 (75)	10 (8)	-
	Time 2	0.061 (0.493)	0.617 (0.523, 0.711)	-0.905, 1.028	69 (112)	9 (14)	14 (23)	9 (14)
	Time 3	0.033 (0.284)	0.813 (0.753, 0.873)	-0.525, 0.590	83 (100)	8 (10)	6 (7)	3 (3)
**Inpatient days**	Time 0	0.508 (5.513)	0.449 (0.359, 0.540)	-10.298, 11.313	84 (103)	8 (10)	6 (7)	2 (3)
	Time 1	0.000 (8.028)	0.544 (0.445, 0.643)	-15.735, 15.735	-	41 (81)	15 (29)	44 (86)
	Time 2	1.093 (11.281)	0.460 (0.342, 0.579)	-21.017, 23.203	66 (107)	2 (3)	15 (24)	17 (27)
	Time 3	1.293 (9.211)	0.197 (0.082, 0.311)	-16.759, 19.346	87 (100)	1 (1)	9 (10)	3 (4)
**Day hospital**	Time 0	0.031 (0.902)	0.037 (-0.075, 0.149)	-1.736, 1.799	94 (238)	-	5 (12)	2 (4)
	Time 1	0.025 (0.221)	-	-0.408, 0.457	99 (161)	-	1 (2)	-
	Time 2	0.006 (0.132)	0.724 (0.670, 0.777)	-0.254, 0.265	98 (169)	-	1 (2)	1 (1)
	Time 3	0.056 (0.319)	0.428 (0.369, 0.487)	-0.569, 0.681	97 (121)	-	3 (4)	-
**Outpatient**	Time 0	0.008 (1.069)	0.537 (0.448 to 0.625)	-2.087 to 2.103	67 (164)	11 (28)	11 (26)	11 (28)
	Time 1	-0.015 (0.272)	0.417 (0.303 to 0.530)	-0.548 to 0.519	93 (188)	3 (6)	1 (3)	3 (6)
	Time 2	-0.047 (0.554)	0.529 (0.420 to 0.637)	-1.134 to 1.039	77 (130)	11 (18)	4 (6)	9 (15)
	Time 3	0.016 (0.589)	0.764 (0.691 to 0.836)	-1.138 to 1.171	72 (88)	10 (12)	8 (10)	10 (12)
**Hospital costs**	Time 0	177.437 (1654.363)	0.660 (0.597 to 0.723)	-3 065 to 3 420	50 (130)	5 (12)	24 (62)	21 (55)
	Time 1	-420.340 (3 355.633)	0.379 (0.262 to 0.496)	-6 997 to 6 157	-	27 (55)	17 (34)	56 (112)
	Time 2	1 336.827 (4 773.868)	0.295 (0.182 to 0.409)	-8 020 to 10 693	45 (78)	2 (3)	33 (57)	21 (36)
	Time 3	342.110 (3 151.993)	0.261 (0.136 to 0.385)	-5 836 to 6 520	52 (66)	3 (4)	24 (31)	21 (27)

Notes: HRE=extraction from hospital records; SIR=Suitable Informant report; Time 0=3 months prior to baseline assessment; Time 1=1 month post-fracture; Time 2=2 months prior to 3 months post-fracture; Time 3=3 months prior to 6 months post-fracture; ρ_c=Lin's concordance correlation coefficient; Exact(none)=zero use/costs in both sources; Exact (some)=the same frequency or cost in both sources; Under=under-reporting (lower frequency/cost in SIR than HRE); Over=over-reporting (higher frequency/use in SIR than HRE).

For peer review only

CONSORT 2010 checklist of information to include when reporting a pilot or feasibility trial*

Section/Topic	Item No	Checklist item	Reported on page No
**Title and abstract**			
	1a	Identification as a pilot or feasibility randomised trial in the title	1
	1b	Structured summary of pilot trial design, methods, results, and conclusions (for specific guidance see CONSORT abstract extension for pilot trials)	3-4
**Introduction**			
Background and objectives	2a	Scientific background and explanation of rationale for future definitive trial, and reasons for randomised pilot trial	4-5
	2b	Specific objectives or research questions for pilot trial	6
**Methods**			
Trial design	3a	Description of pilot trial design (such as parallel, factorial) including allocation ratio	7
	3b	Important changes to methods after pilot trial commencement (such as eligibility criteria), with reasons	NA
Participants	4a	Eligibility criteria for participants	7-8
	4b	Settings and locations where the data were collected	7
	4c	How participants were identified and consented	8-9
Interventions	5	The interventions for each group with sufficient details to allow replication, including how and when they were actually administered	9-10
Outcomes	6a	Completely defined prespecified assessments or measurements to address each pilot trial objective specified in 2b, including how and when they were assessed	10-11
	6b	Any changes to pilot trial assessments or measurements after the pilot trial commenced, with reasons	NA
	6c	If applicable, prespecified criteria used to judge whether, or how, to proceed with future definitive trial	NA
Sample size	7a	Rationale for numbers in the pilot trial	8
	7b	When applicable, explanation of any interim analyses and stopping guidelines	NA
Randomisation:			
Sequence generation	8a	Method used to generate the random allocation sequence	7
	8b	Type of randomisation(s); details of any restriction (such as blocking and block size)	7
Allocation concealment mechanism	9	Mechanism used to implement the random allocation sequence (such as sequentially numbered containers), describing any steps taken to conceal the sequence until interventions were assigned	7

For peer review only

Implementation	10	Who generated the random allocation sequence, who enrolled participants, and who assigned participants to interventions	6-7
Blinding	11a	If done, who was blinded after assignment to interventions (for example, participants, care providers, those assessing outcomes) and how	NA
	11b	If relevant, description of the similarity of interventions	
Statistical methods	12	Methods used to address each pilot trial objective whether qualitative or quantitative	11-12
**Results**			
Participant flow (a diagram is strongly recommended)	13a	For each group, the numbers of participants who were approached and/or assessed for eligibility, randomly assigned, received intended treatment, and were assessed for each objective	13-14,
	13b	For each group, losses and exclusions after randomisation, together with reasons	13
Recruitment	14a	Dates defining the periods of recruitment and follow-up	7, 14, 17,18
	14b	Why the pilot trial ended or was stopped	
Baseline data	15	A table showing baseline demographic and clinical characteristics for each group	14/15
Numbers analysed	16	For each objective, number of participants (denominator) included in each analysis. If relevant, these numbers should be by randomised group	17/18
Outcomes and estimation	17	For each objective, results including expressions of uncertainty (such as 95% confidence interval) for any estimates. If relevant, these results should be by randomised group	17/18
Ancillary analyses	18	Results of any other analyses performed that could be used to inform the future definitive trial	17-20
Harms	19	All important harms or unintended effects in each group (for specific guidance see CONSORT for harms)	NA
	19a	If relevant, other important unintended consequences	NA
**Discussion**			
Limitations	20	Pilot trial limitations, addressing sources of potential bias and remaining uncertainty about feasibility	21
Generalisability	21	Generalisability (applicability) of pilot trial methods and findings to future definitive trial and other studies	21
Interpretation	22	Interpretation consistent with pilot trial objectives and findings, balancing potential benefits and harms, and considering other relevant evidence	23
	22a	Implications for progression from pilot to future definitive trial, including any proposed amendments	23
**Other information**			
Registration	23	Registration number for pilot trial and name of trial registry	23
Protocol	24	Where the pilot trial protocol can be accessed, if available	**Trial registration:** ISRCTN, 99336264 . Registered on 5 September 2016.

For peer review only

			PERFECTED enhanced recovery (PERFECT-ER) care versus standard acute care for patients admitted to acute settings with hip fracture identified as experiencing confusion: study protocol for a feasibility cluster randomized controlled trial | Trials | Full Text (biomedcentral.com)
Funding	25	Sources of funding and other support (such as supply of drugs), role of funders	24
	26	Ethical approval or approval by research review committee, confirmed with reference number	23

Citation: Eldridge SM, Chan CL, Campbell MJ, Bond CM, Hopewell S, Thabane L, et al. CONSORT 2010 statement: extension to randomised pilot and feasibility trials. BMJ. 2016;355.
*We strongly recommend reading this statement in conjunction with the CONSORT 2010, extension to randomised pilot and feasibility trials, Explanation and Elaboration for important clarifications on all the items. If relevant, we also recommend reading CONSORT extensions for cluster randomised trials, non-inferiority and equivalence trials, non-pharmacological treatments, herbal interventions, and pragmatic trials. Additional extensions are forthcoming: for those and for up to date references relevant to this checklist, see www.consort-statement.org.

For peer review only
